# A genus-level classification of the ant subfamily Ponerinae (Hymenoptera, Formicidae)

**DOI:** 10.3897/zookeys.1264.173399

**Published:** 2025-12-19

**Authors:** Brian L. Fisher, Michael G. Branstetter, Bonnie B. Blaimer, Marek L. Borowiec, Gabriela P. Camacho, Maël Doré, Philip S. Ward, John T. Longino

**Affiliations:** 1 Entomology, California Academy of Sciences, San Francisco, CA 94118, USA Entomology, California Academy of Sciences San Francisco United States of America; 2 U.S. Department of Agriculture, Agricultural Research Service, Pollinating Insects Research Unit, Utah State University, Logan, UT, 84322, USA Utah State University Logan United States of America; 3 Museum für Naturkunde, Leibniz-Institute for Evolution and Biodiversity Science, Center for Integrative Biodiversity Discovery, Invalidenstr. 43, 10115 Berlin, Germany Leibniz-Institute for Evolution and Biodiversity Science, Center for Integrative Biodiversity Discovery Berlin Germany; 4 Department of Agricultural Biology and C. P. Gillette Museum, Colorado State University, Fort Collins, CO 80523, USA Colorado State University Fort Collins United States of America; 5 Museu de Zoologia, Universidade de São Paulo, São Paulo, SP 04263-000, Brazil Universidade de São Paulo São Paulo Brazil; 6 Department of Entomology and Nematology, University of California, Davis, CA 95616, USA University of California Davis United States of America; 7 School of Biology, University of Utah, Salt Lake City, Utah, 84112, USA University of Utah Salt Lake City United States of America

**Keywords:** Ants, identification key, morphology, phylogenetics, Ponerinae, systematics, taxonomy, ultraconserved elements

## Abstract

The genus-level classification of the ant subfamily Ponerinae (Hymenoptera: Formicidae) is revised based on a comprehensive phylogenomic analysis of more than 2,300 ultraconserved element (UCE) loci across 1,170 sampled specimens representing 1,020 taxa (600 valid species and 420 morphospecies) and all described ponerine genera known from workers. While most previously defined genus groups are recovered as monophyletic, several genera are shown to be polyphyletic or paraphyletic. To resolve these inconsistencies, four new genera are described: *Boltonopone***gen. nov.**, *Makebapone***gen. nov.**, *Subiridopone***gen. nov.**, and *Sritoponera***gen. nov.***Xiphopelta***stat. rev.** is revalidated and *Euponera* is restricted by expanding *Fisheropone* to absorb a paraphyletic assemblage. *Mesoponera* is split into four lineages, resulting in transfers to *Makebapone*, *Subiridopone*, and *Xiphopelta*. *Iroponera***syn. nov.** is synonymized under *Cryptopone* and additional new synonymies at both the generic and species levels are established. Morphological diagnoses are revised for each affected genus, and updated species lists and new combinations are provided. The updated classification recognizes 54 valid genera within Ponerinae and acknowledges an additional lineage that will be formally described in a subsequent publication. To support identification and comparative studies, revised keys to all extant Ponerinae genera are provided, presented by biogeographic region (African and Malagasy, Palearctic–Indomalaya–Australasia, and New World). This classification is intended to provide a stable, phylogenetically informed framework for future research on ponerine ants.

## Introduction

The ant subfamily Ponerinae is among the most diverse and ecologically significant clades of the Formicidae, currently comprising approximately 50 valid genera and more than 1,200 described species (AntCat 2025). Historically, ponerine classification has relied heavily on external morphology, including diagnostic features often associated with predation, such as specialized mandibles and mesosoma shape (Bolton 2003; Schmidt and Shattuck 2014). Early frameworks subdivided Ponerinae into a collection of genera and informal species groups, though many of these were later shown to be paraphyletic or artificial groupings. A landmark reclassification by Schmidt and Shattuck (2014) formalized a set of genus groups based on detailed morphological comparisons supported by limited molecular evidence, offering a major advance over earlier, more fragmentary schemes. Nonetheless, several problematic or ambiguous taxa remained unresolved, with some genera only tentatively placed and others suspected of being non-monophyletic.

In particular, genera such as *Bothroponera*, *Mesoponera*, *Euponera*, and *Cryptopone* have long been suspected to contain multiple divergent lineages insufficiently delimited by morphology alone. Additional uncertainty surrounded several rare or poorly known taxa whose placement has historically been tentative, e.g., *Fisheropone*, *Parvaponera*, *Wadeura*, *Iroponera*, and *Belonopelta*, as well as highly diverse genera such as *Leptogenys*, *Brachyponera*, and *Neoponera*, which have exhibited inconsistent delimitation across regional faunas and prior treatments. These cases, together with the presence of previously unsampled genera, highlighted the persistent need for a comprehensive phylogenomic test of generic boundaries and a stable, monophyly-based classification of Ponerinae.

The past decade has witnessed a profound methodological shift in ant systematics, with molecular phylogenetics and phylogenomics transforming the resolution of relationships across the Formicidae. In particular, the application of ultraconserved element (UCE) loci has dramatically improved the ability to recover robust and well-supported phylogenies at both deep and shallow taxonomic levels (Faircloth 2016; Branstetter et al. 2017). This revolution has enabled researchers to revisit long-standing taxonomic challenges with far greater confidence, including the relationships within the poneromorph clade and the Ponerinae more specifically (Branstetter et al. 2017). Several studies have begun to integrate genome-scale datasets with extensive taxon sampling, setting a new standard for understanding subfamily-level and genus-level patterns of ant evolution (Blaimer et al. 2015; Ward et al. 2016; Borowiec 2019; Borowiec et al. 2025).

Building on these advances, recent comprehensive phylogenomic work has confirmed the monophyly of Ponerinae and the validity of its two major tribes, Platythyreini and Ponerini, while clarifying deep relationships among the main genus groups (Doré et al. 2025). That analysis demonstrated the stability of most genus groups as proposed by Schmidt (2013) and Schmidt and Shattuck (2014), while simultaneously revealing the need for changes in the aforementioned problematic taxa.

A stable, monophyly-based genus-level classification is urgently needed to support future taxonomic and biodiversity research on Ponerinae. Resolving the paraphyly and polyphyly of current genera will not only align taxonomy with evolutionary history but also provide a vital backbone for renewed species-level inventories and revisions within the subfamily. Such a classification will facilitate consistent identification, ecological studies, and comparative research, serving as a replicable model for other ant subfamilies where similar challenges remain. Establishing a robust and phylogenetically coherent framework is an essential prerequisite before resources are invested in large-scale species inventories and conservation assessments, ensuring that future efforts are built on a sound and stable taxonomic foundation.

The primary goal of this paper is to establish a classification in which all ponerine genera are monophyletic. We rely on the molecular results to provide the evidence for monophyly. We resolve all cases of polyphyletic and paraphyletic genera, either by synonymy or description of new genera. We attempt to define each genus morphologically, often aided by geographical separation of clades, but do not require that the morphological features defining each genus are unequivocal synapomorphies. We provide revised keys to genera, separately for the same three biogeographic regions used by Schmidt and Shattuck (2014): African and Malagasy, Palearctic–Indomalaya–Australasia (“Eurasian and Australian” in Schmidt and Shattuck), and the New World. All new combinations of species and species lists for reorganized genera are presented in Suppl. material 1.

## Molecular methods

### Molecular sampling

Our molecular dataset comprised 1,170 samples, including 1,167 ingroup terminals representing 1,020 unique taxa of Ponerinae (600 valid species and 420 morphospecies), and three outgroup taxa: *Paraponera clavata* (Fabricius), *Amblyopone australis* Erichson, and *Proceratium google* Fisher (see Suppl. material 2). To maximize both taxonomic and geographic representation, we included multiple species per genus, and for approximately 150 species or morphospecies we analyzed more than one specimen to capture geographic or intraspecific variation. *Igaponera* Troya et al., 2022, known from a single queen, is the only genus that was not included in the molecular analysis. Additionally, we analyzed a reduced dataset of 261 samples to enable focused phylogenetic resolution, while maintaining balanced taxon sampling across major lineages and regions. Among these samples, 208 were newly generated for this study (NCBI BioProject# PRJNA1291805) while 962 were sequenced previously (Branstetter et al. 2017; Branstetter and Longino 2019, 2022; Longino and Branstetter 2020; Camacho et al. 2025; Doré et al. 2025) with corresponding sequence data available in the NCBI Sequence Read Archive (BioProject#s PRJNA360290, PRJNA513200, PRJNA563172, PRJNA778536, PRJNA1196817 and PRJNA1125038).

### DNA sequencing and library preparation

To recover a comprehensive set of loci across taxa, we employed a phylogenomic approach targeting ultraconserved elements (UCEs) via sequence capture and high-throughput sequencing, following the protocol of Branstetter et al. (2017). Library preparation, enrichment, and sequencing were conducted either in-house or through external facilities (RAPiD Genomics, Gainesville, FL; University of Utah Genomics Core Facility, Salt Lake City, UT). Standardized protocols included genomic DNA extraction, construction of dual-indexed libraries, UCE hybrid enrichment using the hym-v2 ant-specific probe set, equimolar pooling, and paired-end sequencing on Illumina HiSeq 2500 (PE125), HiSeq X (PE150), or NovaSeq (PE150) platforms. The hym-v2 ant-specific bait set targets 2,524 conserved loci across Hymenoptera and has been widely validated in ant phylogenetics for resolving both shallow and deep evolutionary splits (Branstetter et al. 2017; Branstetter and Longino 2019; Camacho et al. 2021, 2022, 2025).

### Assembly and UCE matrix generation

Raw sequencing reads were processed using the Phyluce v. 1.7 package (Faircloth 2016), largely adhering to procedures outlined by Branstetter et al. (2017). Quality trimming was performed using Illumiprocessor (Faircloth 2013), which integrates Trimmomatic (Bolger et al. 2014). Cleaned reads were assembled de novo with SPAdes v. 3.14.1 (Prjibelski et al. 2020), and UCE loci were identified by aligning contigs to the hym-v2 ant-specific bait set using LASTZ v. 1.02 (Harris 2007), maintaining default Phyluce settings. Assembly and UCE recovery statistics, including the number of loci recovered per specimen and mean locus length, were computed using Phyluce utilities. Multiple sequence alignments were conducted with MAFFT v. 7.130b (Katoh and Standley 2013) run with Phyluce using default settings. We removed poorly aligned regions with TrimAl v. 1.4.rev15 (Capella-Gutiérrez et al. 2009). We retained only loci present in at least 75% of taxa for both datasets. Final summary statistics for the filtered and trimmed set of alignments were generated with Phyluce utilities.

### Phylogenetic analyses

Phylogenetic relationships were inferred using Maximum Likelihood (ML) in IQ-TREE v. 2.4.0 (Minh et al. 2020). Unpartitioned analyses of both datasets used the GTR+G4 model, yielding consensus tree log-likelihoods of –50,041,566.128 (1170t dataset) and –32,967,652.130 (261t dataset). For the reduced 261t dataset, we also tested a partitioned model using the Sliding Window Site Characteristics–Entropy (SWSC-EN) approach with merging (Tagliacollo and Lanfear 2018), which accounts for variation in sequence conservation across UCE loci. Optimal partitioning was determined with ModelFinder2 (Kalyaanamoorthy et al. 2017), using the GTR+F+G4 model and the rclusterf algorithm under AICc. This scheme identified 7,037 partitions and showed improved model fit over the unpartitioned analysis (log-likelihood: –32,198,946.918). Following the merging of SWSC-EN partitions, full model selection was performed on the final partitioning scheme using the “-m MFP” command. Branch support for each analysis was assessed using 1,000 ultrafast bootstrap replicates (UFBS; Hoang et al. 2018) and SH-like approximate likelihood ratio tests (SH-aLRT; Guindon et al. 2010).

## Results

### Molecular analyses

For the 208 newly sequenced samples, we obtained a mean of 2,061 UCE loci (range: 249–2,430) and an average UCE contig length of 902 bp (range: 257–1,505 bp). After alignment, trimming, and filtering, the resulting 1,170t UCE matrix comprised 2,364 UCE loci, 1,042,101 aligned base pairs (625,897 informative); and the 261t matrix yielded 2,379 UCE loci, 1,326,010 aligned base pairs (697,824 informative). The mean per-locus trimmed alignment length was 440.8 bp (range: 58–3,926 bp) for the 1,170t matrix, and 557.4 bp (range: 65–1,939 bp) for the 261t matrix.

**Figure 1.**
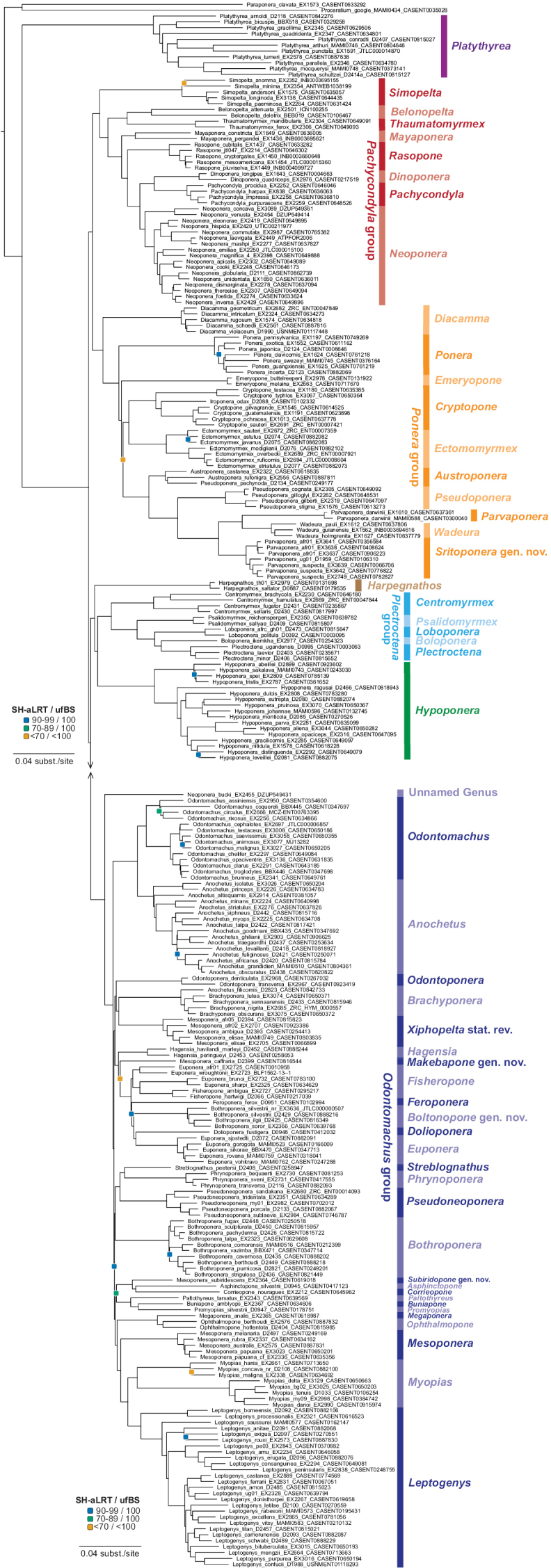
Phylogeny of the ant subfamily Ponerinae. Maximum likelihood phylogeny generated in IQ-TREE v. 2.4.0 from the reduced 261-taxon dataset employing 7,037 data partitions and full model testing. All branches are 100% supported by ultrafast bootstraps (ufBS) and SH-like approximate likelihood tests (SH-aLRT), except where indicated by the colored squares on the subtended node. Dark blue squares: SH-aLRT = 90–99 and ufBS = 100; teal squares: SH-aLRT = 70–89 and ufBS = 100; orange squares: SH-aLRT < 70 and ufBS < 100. The new genus-level classification is indicated with the vertical colored bars; genus groups are indicated for the five groups containing more than one genus.

The seven genus groups of Schmidt and Shattuck (2014) were largely supported as clades in our phylogeny (Fig. 1, Suppl. material 3), and we retain them in our synopsis. Only two genera differed in placement: *Feroponera* and *Dolioponera*. While Schmidt and Shattuck placed these in the *Plectroctena* genus group, our phylogeny recovers them within the *Odontomachus* genus group. Schmidt and Shattuck also anticipated that several problematic genera might prove to be non-monophyletic, and in most cases, our results confirmed their suspicions of paraphyly or polyphyly. *Bothroponera* comprised two separate clades, requiring the description of a new genus. *Mesoponera* contained four distinct clades, leading us to describe two new genera and resurrect one genus (*Makebapone* gen. nov., *Subiridopone* gen. nov., and *Xiphopelta* stat. rev.). Although the backbone node subtending *Makebapone* and its sister taxa received low support in the 261-sample tree (Fig. 1), its placement was identical in the 1170 sample tree (Suppl. material 3), where we included five samples, and this result received nearly maximum support (99.9/100). The genus is also strongly supported by a coherent set of morphological features distinguishing it from *Mesoponera*. *Parvaponera* was found to be paraphyletic with respect to *Wadeura*, necessitating a new genus (*Sritoponera* gen. nov.) to restore monophyly. *Euponera* was split into two clades, *Euponera* (s.s.) and a paraphyletic assemblage relative to *Fisheropone*; by expanding the definition of *Fisheropone* (and transferring the relevant *Euponera* species to it), we make *Euponera* monophyletic. We also discovered that the rare Australian genus *Iroponera* is a highly derived *Cryptopone*, requiring its synonymy under *Cryptopone*.

*Neoponera bucki* is a distinctive Neotropical species that has been placed in five different genera since its description in 1927. Our phylogeny places it as an isolated lineage within the *Odontomachus* genus group, sister to all other members of the group (Fig. 1, Suppl. material 3, unnamed genus). This species will require the description of a new genus, but we refrain from naming it here, deferring to a separate project in which it will be described. In addition, the phylogenetic placement of the several Afrotropical *Loboponera* species (Suppl. material 3) is inconsistent with the current morphological circumscription of the genus, suggesting that further taxon sampling and integrative revision will be necessary to fully resolve generic limits within *Loboponera*.

### Reclassification of Ponerinae

Genus group classification unchanged from Schmidt and Shattuck (2014).

#### *Harpegnathos* genus group

*Harpegnathos* Jerdon, 1851

= *Drepanognathus* Smith, 1858

#### *Hypoponera* genus group

*Hypoponera* Santschi, 1938

#### *Odontomachus* genus group

*Anochetus* Mayr, 1861

= *Stenomyrmex* Mayr, 1862

*Asphinctopone* Santschi, 1914b

= *Lepidopone* Bernard, 1953

*Boltonopone* gen. nov.

*Bothroponera* Mayr, 1862

*Brachyponera* Emery, 1900a

= *Myrmapatetes* Wheeler, 1929a, syn. nov.

*Buniapone* Schmidt & Shattuck, 2014

*Corrieopone* Esteves & Fisher, 2021

*Dolioponera* Brown, 1974

*Euponera* Forel, 1891

*Feroponera* Bolton & Fisher, 2008

*Fisheropone* Schmidt & Shattuck, 2014

*Hagensia* Forel, 1901c

*Leptogenys* Roger, 1861

= *Dorylozelus* Forel, 1915

= *Lobopelta* Mayr, 1862

= *Machaerogenys* Emery, 1911

= *Microbolbos* Donisthorpe, 1948a

= *Odontopelta* Emery, 1911

= *Prionogenys* Emery, 1895a

*Makebapone* gen. nov.

*Megaponera* Mayr, 1862

*Mesoponera* Emery, 1900b

*Myopias* Roger, 1861

= *Bradyponera* Mayr, 1886

= *Trapeziopelta* Mayr, 1862

*Neoponera bucki* (Borgmeier, 1927) [see text explanation]

*Odontomachus* Latreille, 1804

= *Champsomyrmex* Emery, 1892

= *Myrtoteras* Matsumura, 1912

= *Pedetes* Bernstein, 1861

*Odontoponera* Mayr, 1862

*Ophthalmopone* Forel, 1890

*Paltothyreus* Mayr, 1862

*Phrynoponera* Wheeler, 1920

*Promyopias* Santschi, 1914b

*Pseudoneoponera* Donisthorpe, 1943b

*Streblognathus* Mayr, 1862

*Subiridopone* gen. nov.

*Xiphopelta* Forel, 1913a, stat. rev.

#### *Pachycondyla* genus group

*Belonopelta* Mayr, 1870

= *Leiopelta* Baroni Urbani, 1975

*Dinoponera* Roger, 1861

*Igaponera* Troya et al., 2022

*Mayaponera* Schmidt & Shattuck, 2014

*Neoponera* Emery, 1901

= *Eumecopone* Forel, 1901b

= *Syntermitopone* Wheeler, 1936

= *Termitopone* Wheeler, 1936

*Pachycondyla* Smith, 1858

*Rasopone* Schmidt & Shattuck, 2014

*Simopelta* Mann, 1922

*Thaumatomyrmex* Mayr, 1887

#### *Platythyrea* genus group

*Platythyrea* Roger, 1863

= *Eubothroponera* Clark, 1930

#### *Plectroctena* genus group

*Boloponera* Fisher, 2006

*Centromyrmex* Mayr, 1866

= *Glyphopone* Forel, 1913b

= *Leptopone* Arnold, 1916

= *Spalacomyrmex* Emery, 1889

= *Typhloteras* Karavaiev, 1925

*Loboponera* Bolton & Brown, 2002

*Plectroctena* Smith, 1858

= *Cacopone* Santschi, 1914b

*Psalidomyrmex* André, 1890

#### *Ponera* genus group

*Austroponera* Schmidt & Shattuck, 2014

*Cryptopone* Emery, 1893a

= *Iroponera* Schmidt & Shattuck, 2014, syn. nov.

*Diacamma* Mayr, 1862

*Ectomomyrmex* Mayr, 1867a

*Emeryopone* Forel, 1912

*Parvaponera* Schmidt & Shattuck, 2014

*Ponera* Latreille, 1804

= *Pseudocryptopone* Wheeler, 1933

= *Pteroponera* Bernard, 1950

= *Selenopone* Wheeler, 1933

*Pseudoponera* Emery, 1900a

= *Trachymesopus* Emery, 1911

*Sritoponera* gen. nov.

*Wadeura* Weber, 1939

### Keys to Ponerine genera

Following Schmidt and Shattuck (2014), we provide separate worker keys for three broad faunal regions: Afrotropical and Malagasy; Palearctic–Indomalaya–Australasia (Schmidt and Shattuck’s “Eurasian and Australian” region); and New World. These divisions are intended solely for practical identification and reflect conventional faunal usage rather than discrete biogeographic or evolutionary units. Recent phylogenetic and biogeographic analyses (e.g., Doré et al. 2025) demonstrate that ponerine lineages often cross these regional boundaries. Accordingly, our regional keys are structured to facilitate accurate morphological identification, not to imply strict evolutionary separations. Annotations in keys are as follows: (part) — indicates that only a subset of species of the genus key out at that couplet, typically due to morphological heterogeneity; (species name) — indicates that the couplet applies to a single, named species; (region) — geographic qualifiers (e.g., Seychelles) are provided only when they aid in distinguishing morphologically similar taxa.

#### Key to Afrotropical and Malagasy ponerine ant genera (workers) modified from Fisher and Bolton (2016)

**Table:** 

1	Mandible long and linear, in full-face view inserted in the middle of the anterior margin of the head; mandible bases closely approximated (A)	**2**
–	Mandible linear to triangular, in full-face view inserted at the anterolateral corners of the head; mandible bases conspicuously separated (AA)	**3**
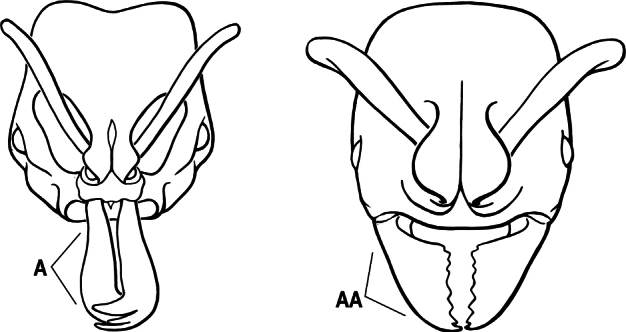		
2(1)	Nuchal carina converging in a V-shape at midline (A), receiving dark posterior apophyseal lines converging to form the median-dorsal groove (B); dorsalmost tooth of apical mandibular series truncated (C), rarely acute.	*** Odontomachus ***
–	Nuchal carina forming a broad, uninterrupted curve across posterodorsal head (AA); lacking paired dark apophyseal lines; median groove on vertex absent or shallow and ill-defined (BB); dorsalmost tooth of apical mandibular series acute (CC)	*** Anochetus ***
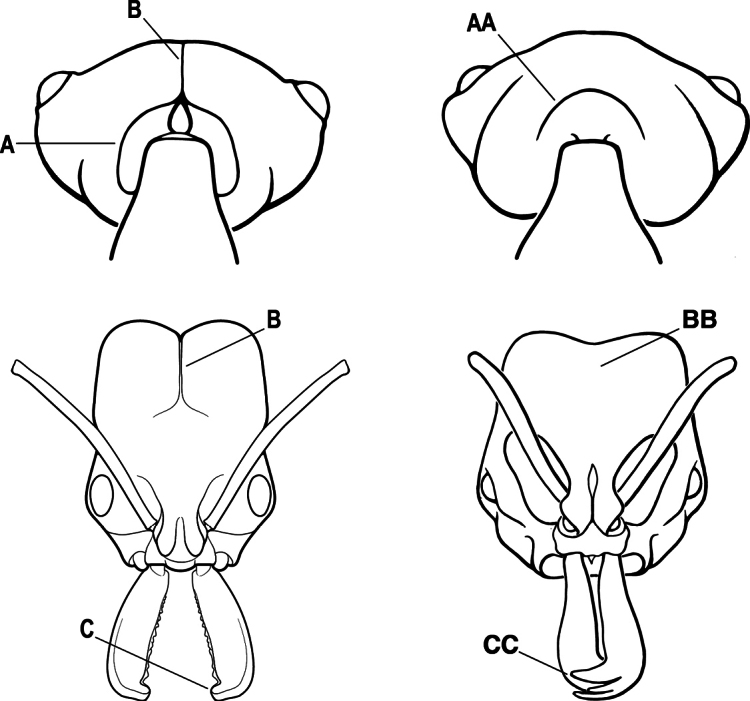		
3(1)	Dorsal (outer) surface of metabasitarsus usually with simple setae, and also equipped with strong, thick spiniform or peg-like traction setae (A); similar traction setae also always present on mesobasitarsus and mesotibia, together with simple setae	**4**
–	Dorsal (outer) surface of metabasitarsus with simple setae but without thick spiniform or peg-like traction setae (AA); traction setae may very rarely occur on either mesobasitarsus or mesotibia, together with simple setae, but if traction setae are present on either, then they are absent from metabasitarsus	**6**
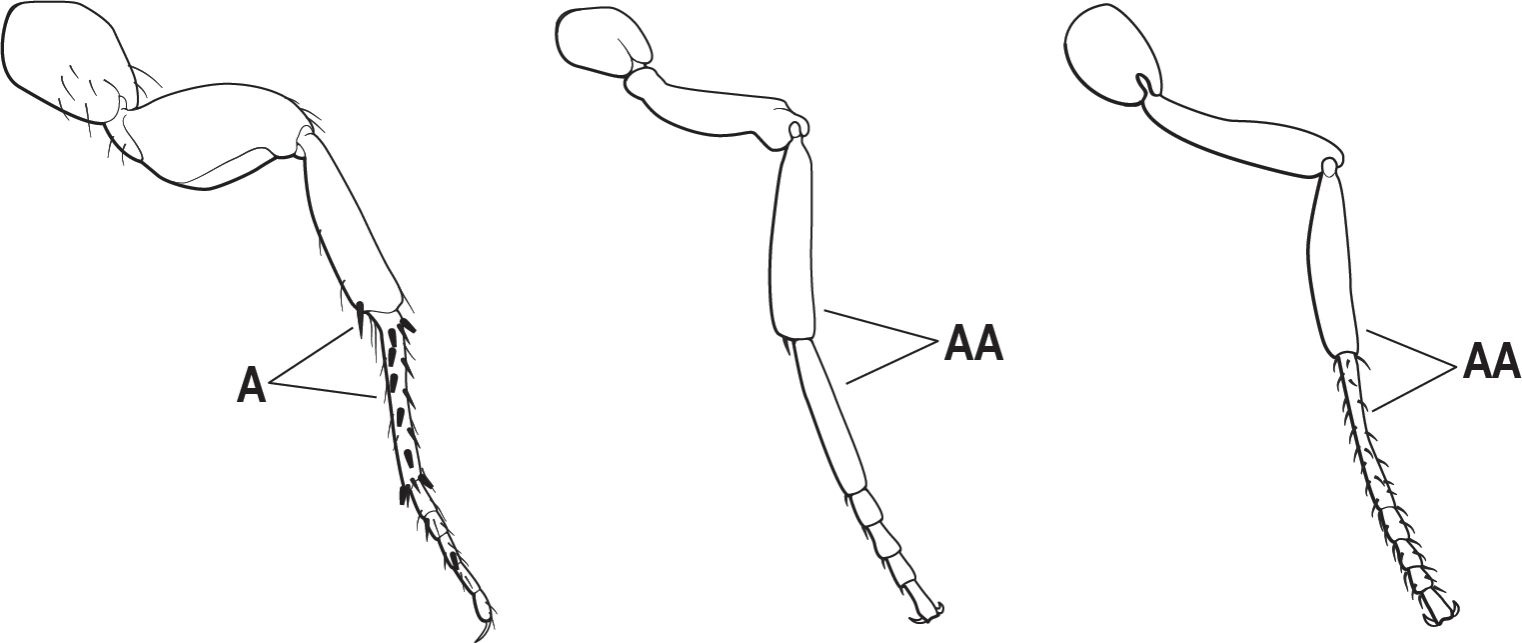		
4(3)	Mandible elongate and narrow, subfalcate (A); apex bearing a short vertical series of 3 or 4 small teeth. Inner margin of apical 1/2 concave; basal margin elongated and shallowly convex (B); prora a thick, blunt, longitudinal crest, extending from just below the helcium nearly to the apex of the first gastral sternite (A3); not projecting forward in profile (C); labial palp with 4 segments	*** Promyopias ***
–	Mandible triangular to elongate-triangular (AA); apex without a short vertical series of 3 or 4 small teeth. Masticatory and basal margins both straight or nearly so (BB); prora a transverse plate or a pair of weak, short longitudinal ridges located on the anterior face of the first gastral sternite (A3); projecting forward and visible in profile (CC); labial palp with 3 segments	**5**
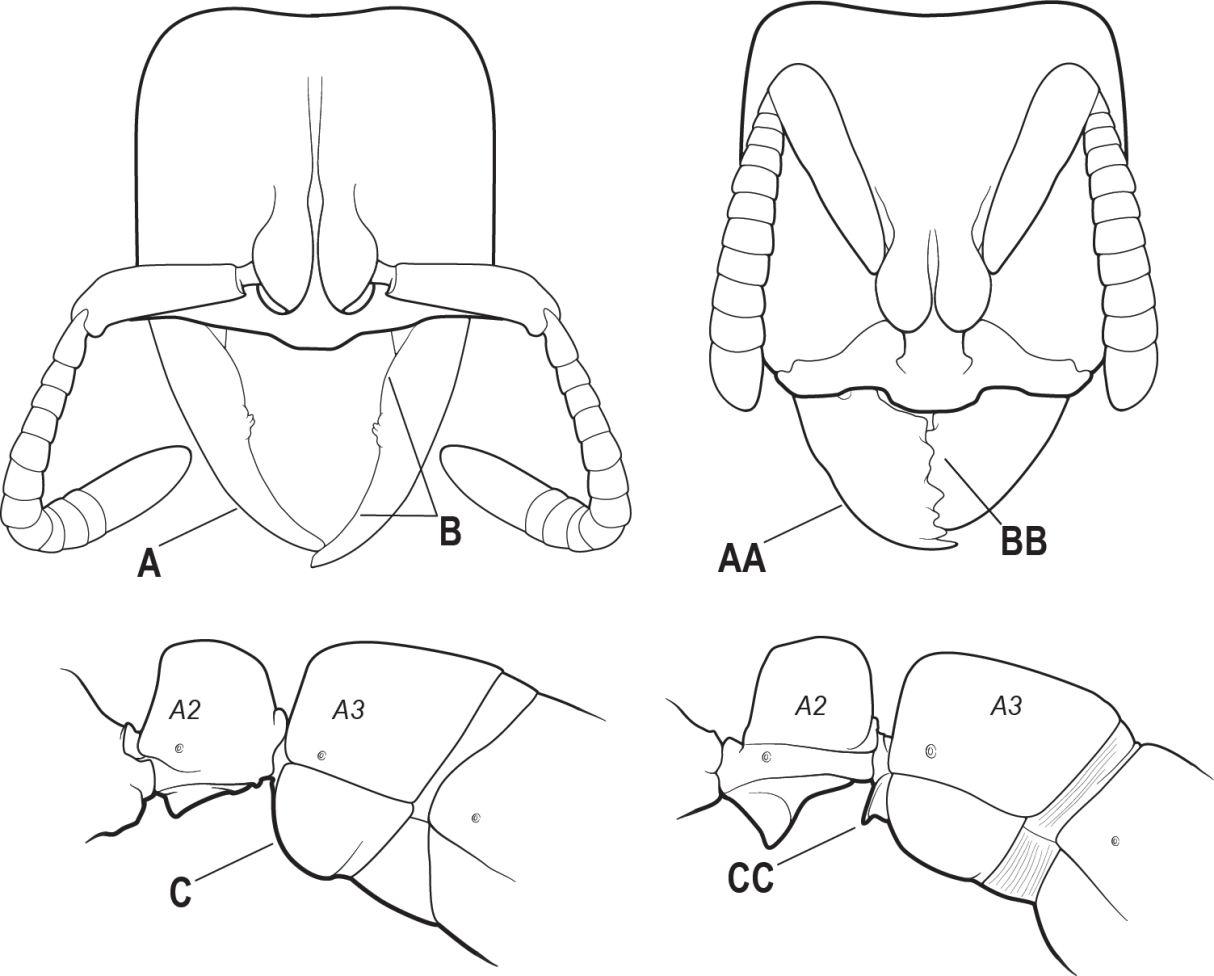		
5(4)	Antennal funiculus gradually incrassate but without a distinct apical club (A); anterior clypeal margin without teeth that overhang the base of the mandible (B); maxillary palp with 4 segments. Orifice of metapleural gland lateral (C); posterior surface of metatibia without a depressed glandular area of pale cuticle above the spur	*** Centromyrmex ***
–	Antennal funiculus with a distinct 4-segmented apical club (AA); anterior clypeal margin with a strong triangular tooth on each side that projects over the base of the mandible (BB); maxillary palp with 2 segments. Orifice of metapleural gland posteroventral (CC); posterior surface of metatibia with a depressed glandular area of pale cuticle above the spur (DD)	*** Feroponera ***
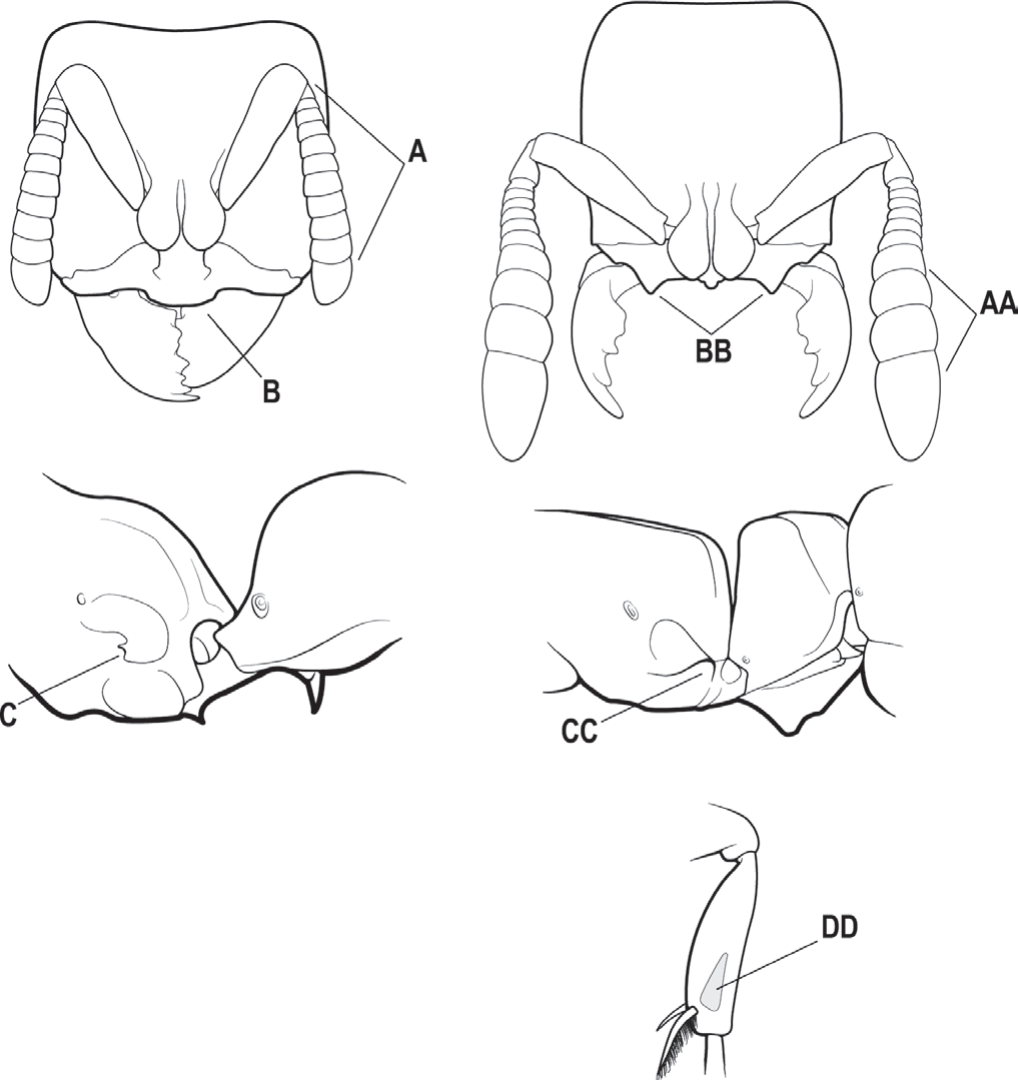		
6(3)	Ventral apex of metatibia, when viewed from in front with the metafemur at right-angles to the body, with a single large pectinate spur; without a second smaller spur in front of the pectinate main spur in the direction of observation (A)	**7**
–	Ventral apex of metatibia, when viewed from in front with the metafemur at right-angles to the body, with two spurs, consisting of a large pectinate spur and a second smaller spur which is in front of the pectinate main spur in the direction of observation (AA)	**16**
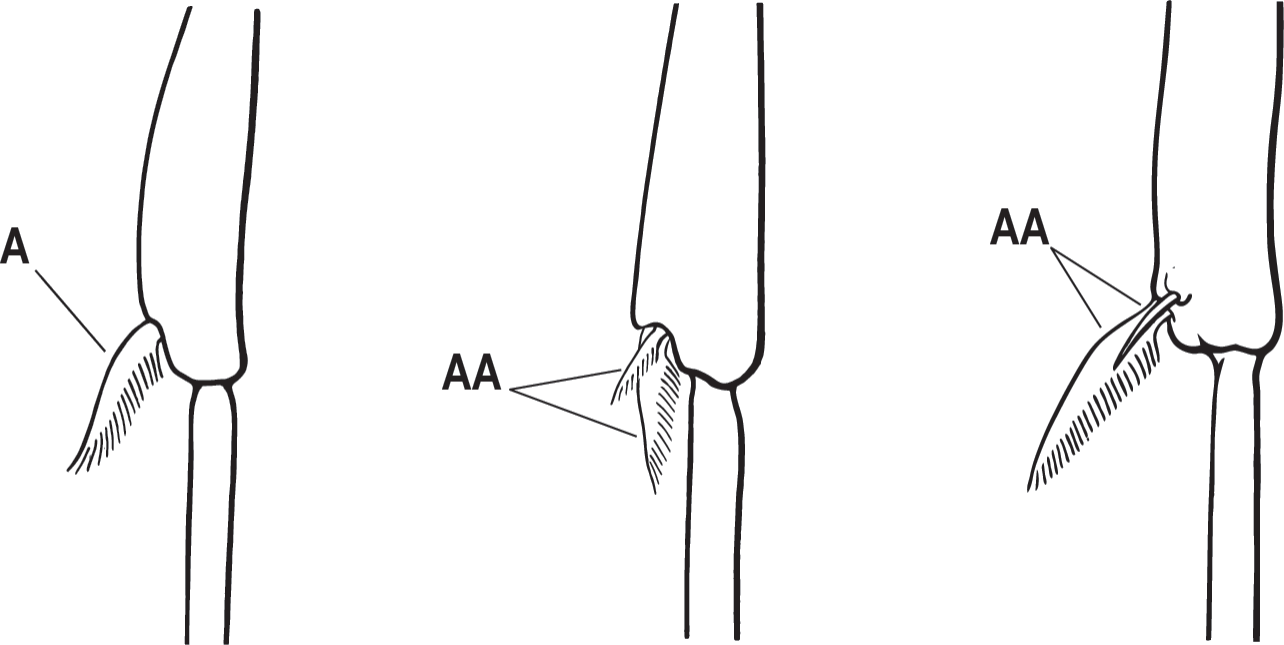		
7(6)	Mandible elongate, linear and weakly curved, the inner margin with 0–2 blunt teeth (A)	**8**
–	Mandible triangular to elongate-triangular, the masticatory margin sometimes edentate but usually with several to many teeth (AA)	**9**
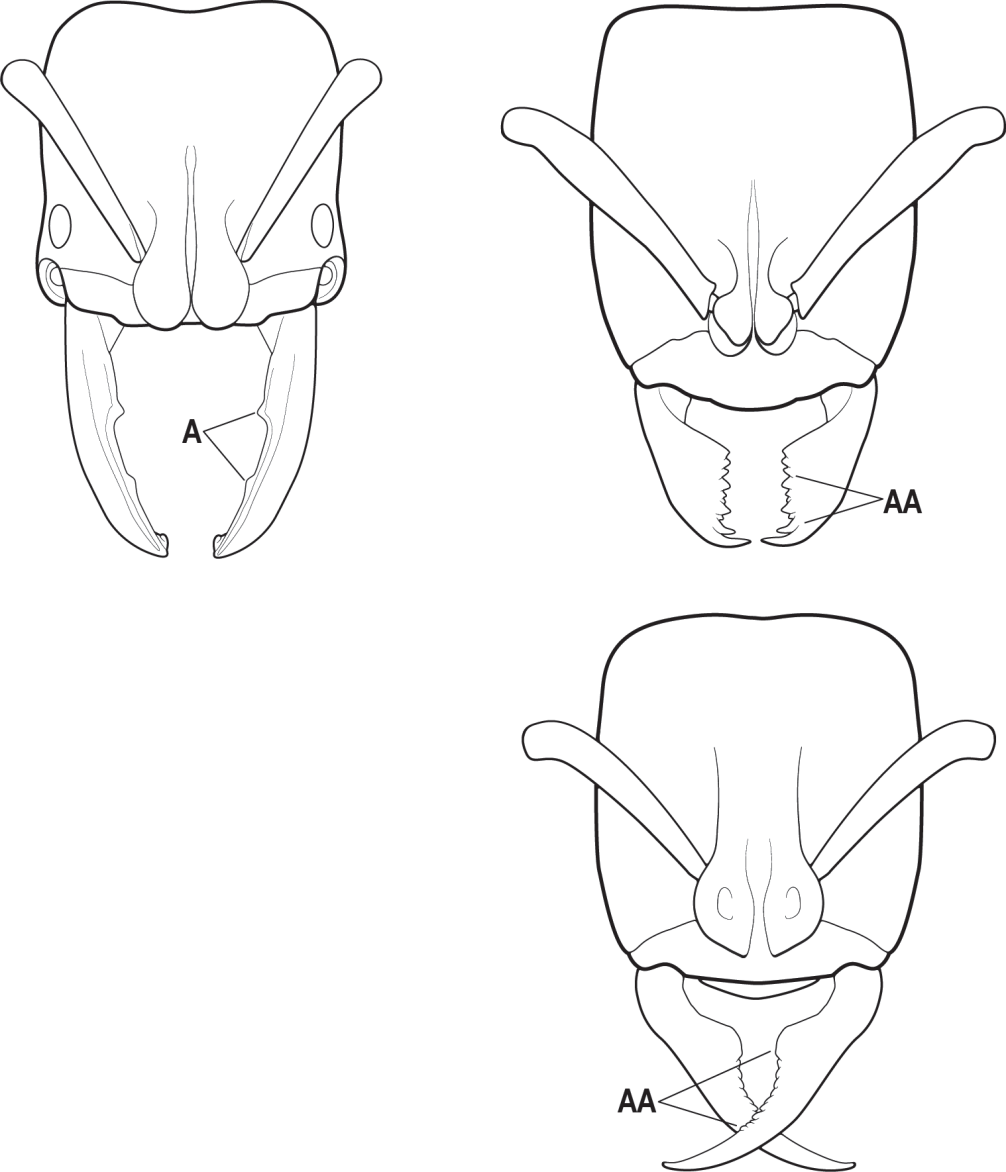		
8(7)	Mandibular articulation associated with a marked semicircular excavation of the dorsolateral anterior margin of the head (A); mandible with a longitudinal groove on the inner 1/2 of the dorsal surface (B)	*** Plectroctena ***
–	Mandibular articulation not associated with a semicircular excavation of the dorsolateral anterior margin of the head (AA); mandible without a longitudinal groove on the inner 1/2 of the dorsal surface (BB)	*** Boloponera ***
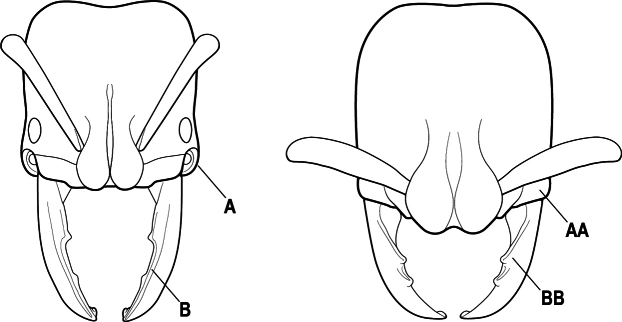		
9(7)	Basal portion of mandible with a distinct circular or near-circular pit or fovea dorsolaterally (A)	***Fisheropone* (part, *F. hartwigi*)**
–	Basal portion of mandible without a dorsolateral pit or fovea (AA)	**10**
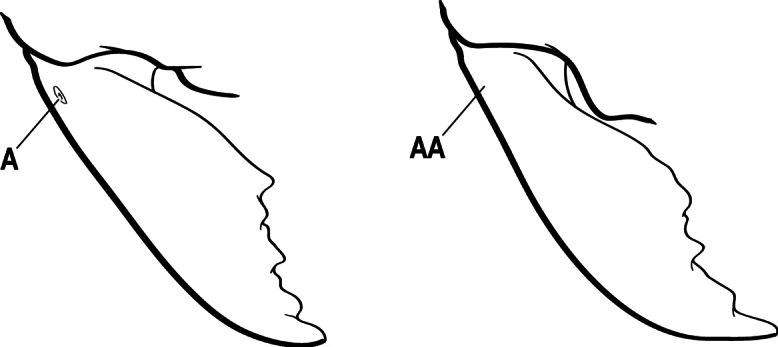		
10(9)	Gaster (A3–A7) in profile and in dorsal view without an impression between the presclerites and postsclerites of the second gastral segment (A4); gaster without a girdling constriction (A)	*** Asphinctopone ***
–	Gaster (A3–A7) in profile and in dorsal view with a distinct impression between the presclerites and postsclerites of the second gastral segment (A4) that appears as a girdling constriction of the gaster (AA)	**11**
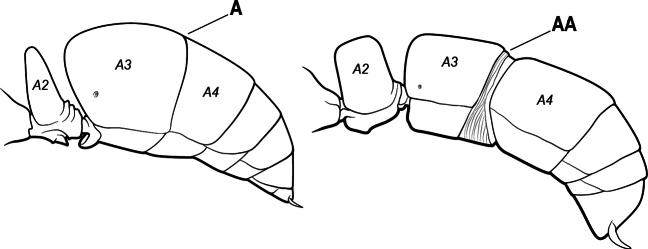		
11(10)	Mandible elongate-falcate, with an extremely long apical tooth so that the tips cross over at rest (A); masticatory margin edentate or crenulate; labrum prominent, in full-face view projecting beyond the anterior clypeal margin as a striated lobe (B)	*** Psalidomyrmex ***
–	Mandible short and triangular, lacking an extremely long apical tooth (AA); masticatory margin multidentate. Labrum not projecting beyond clypeus as a striated lobe in full-face view (BB)	**12**
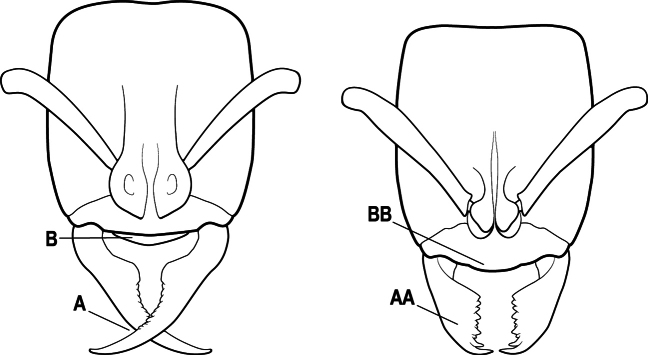		
12(11)	Metafemur mid-dorsally with a longitudinal groove on the basal 1/2 (A); tergite of second gastral segment (A4) with dorsum vaulted, strongly arched and downcurved posteriorly (B); sternite of second gastral segment much reduced and with a bluntly U-shaped outline in profile, very much smaller than the tergite	*** Loboponera ***
–	Metafemur mid-dorsally without a longitudinal groove on the basal 1/2. Tergite of second gastral segment (A4) with dorsum not vaulted, not arched nor strongly downcurved posteriorly (BB); sternite of second gastral segment longitudinal, without a bluntly U-shaped outline in profile, only slightly smaller than the tergite	**13**
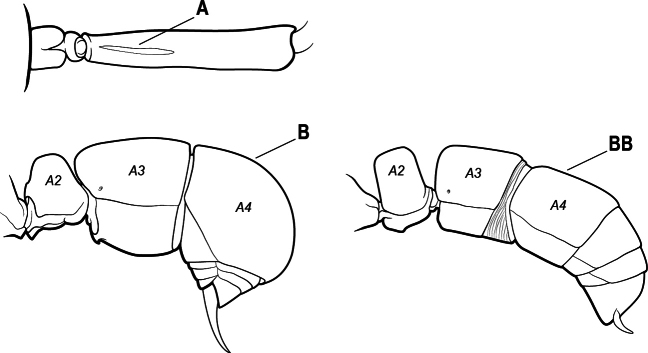		
13(12)	Petiole (A2) in profile subcylindrical, long and low, without an erect scale or node (A); prora absent from first gastral sternite below helcium (B); postsclerites of second gastral segment (A4 posterior to gastral constriction) cylindrical, in profile very much longer than high and much longer than the first segment (C)	*** Dolioponera ***
–	Petiole (A2) in profile an erect scale or node (AA); prora present on first gastral sternite below helcium (BB); postsclerites of second gastral segment (A4 posterior to gastral constriction) not cylindrical, in profile as high as long or nearly so and at most only slightly longer than the first segment (CC)	**14**
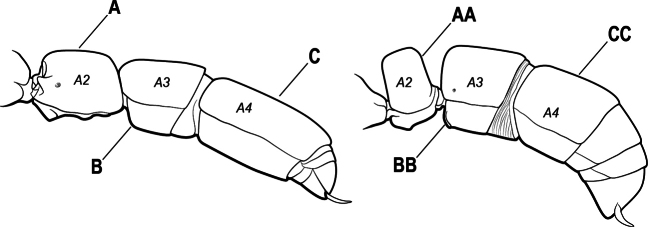		
14(13)	Mesosoma in dorsal view, anterior 1/2 of propodeum strongly constricted from side to side, its dorsum reduced to a mere longitudinal crest, almost obliterated (A); mandible narrowly triangular and slender, with a total of 7 teeth and denticles, or fewer (B); exposed length of closed mandible 0.45 × maximum length of head, or usually more	***Fisheropone* (part, *F. ambigua*)**
–	Mesosoma in dorsal view, anterior 1/2 of propodeum forming a distinctly defined transverse dorsal surface (AA); mandible broadly triangular and stout, usually with a total of more than 8 teeth and denticles (BB); exposed length of closed mandible at most 0.35 × maximum length of head, usually less	**15**
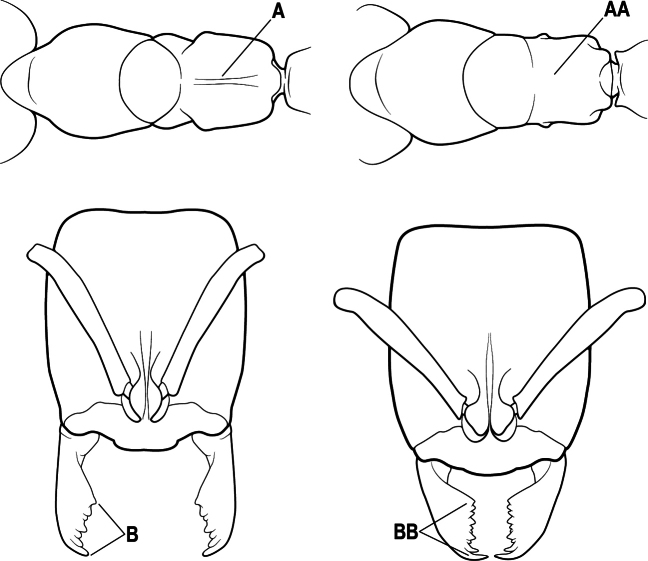		
15(14)	Subpetiolar process in profile with a pair of teeth posteroventrally (A) and with a fenestra or thin-spot anteriorly, which is translucent (B); maxillary palp with 2 segments	*** Ponera ***
–	Subpetiolar process in profile rounded to acutely angulate posteroventrally, but never with a pair of teeth (AA); an anterior fenestra or thin-spot usually absent but present in some species (BB); maxillary palp with 0 or 1 segment	*** Hypoponera ***
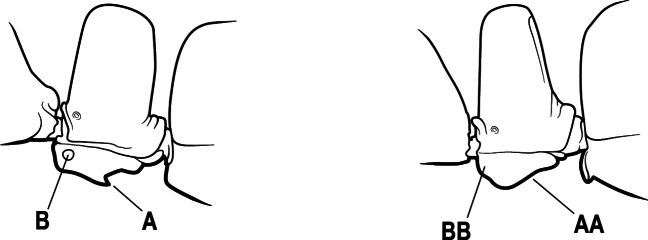		
16(6)	Helcium in profile located approximately at mid-height on the front of the first gastral segment (A3), so that the first gastral segment does not have a long vertical anterior face in profile (A); mesotibia and metatibia each with two pectinate spurs, the anterior spur smaller than the posterior (B)	*** Platythyrea ***
–	Helcium in profile located very low on the front of the first gastral segment (A3) so that the first gastral segment has a long vertical anterior face (AA); mesotibia and metatibia each with one large pectinate spur and one small simple spur (BB)	**17**
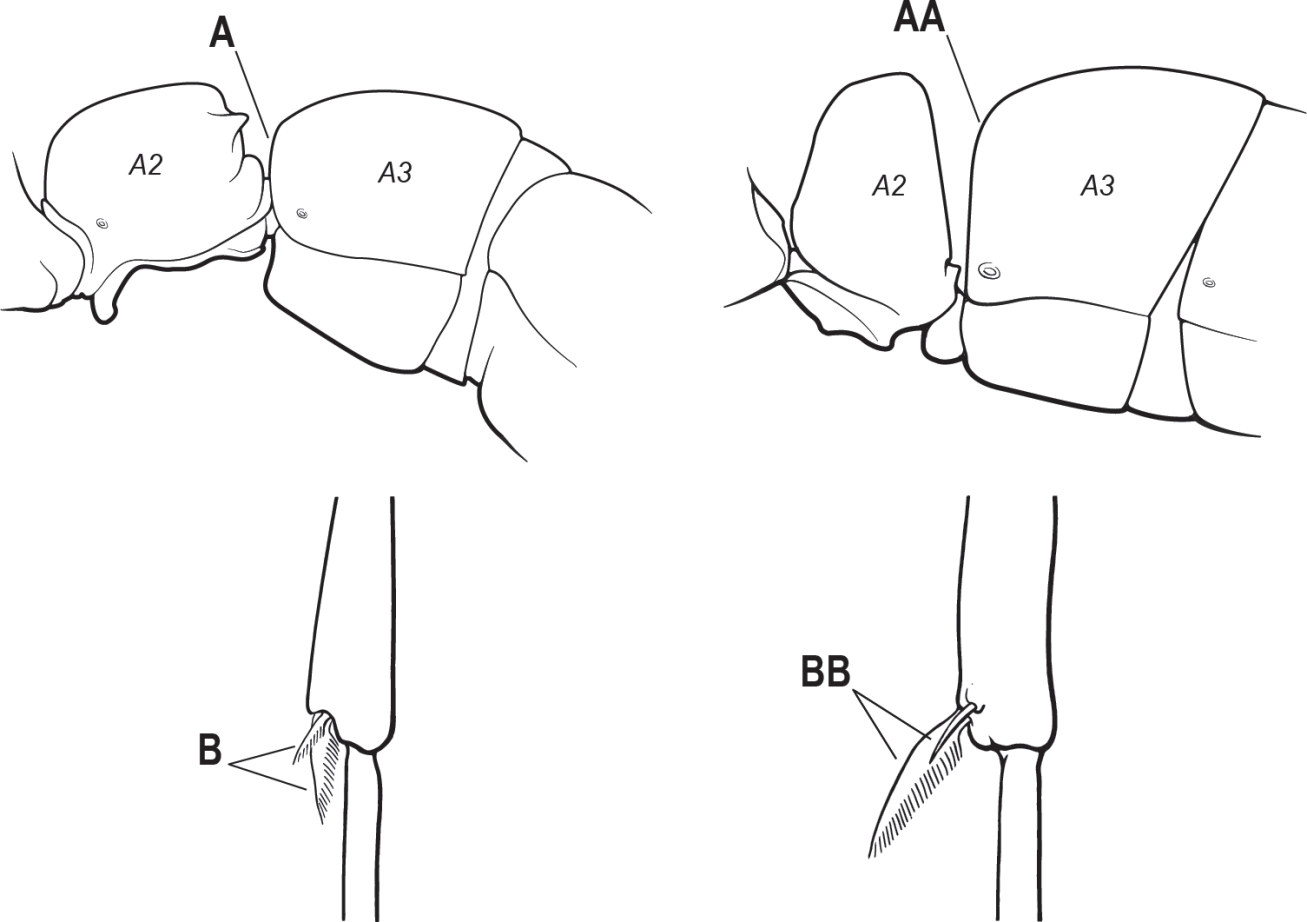		
17(16)	Hypopygium with a series of dorsally directed teeth or spiniform setae on its dorsal margin posteriorly (may be concealed among the regular setae of the hypopygial apex) (A)	**18**
–	Hypopygium unarmed; usually with regular setae on its dorsal margin posteriorly, but without a series of dorsally directed teeth or spiniform setae (AA)	**19**
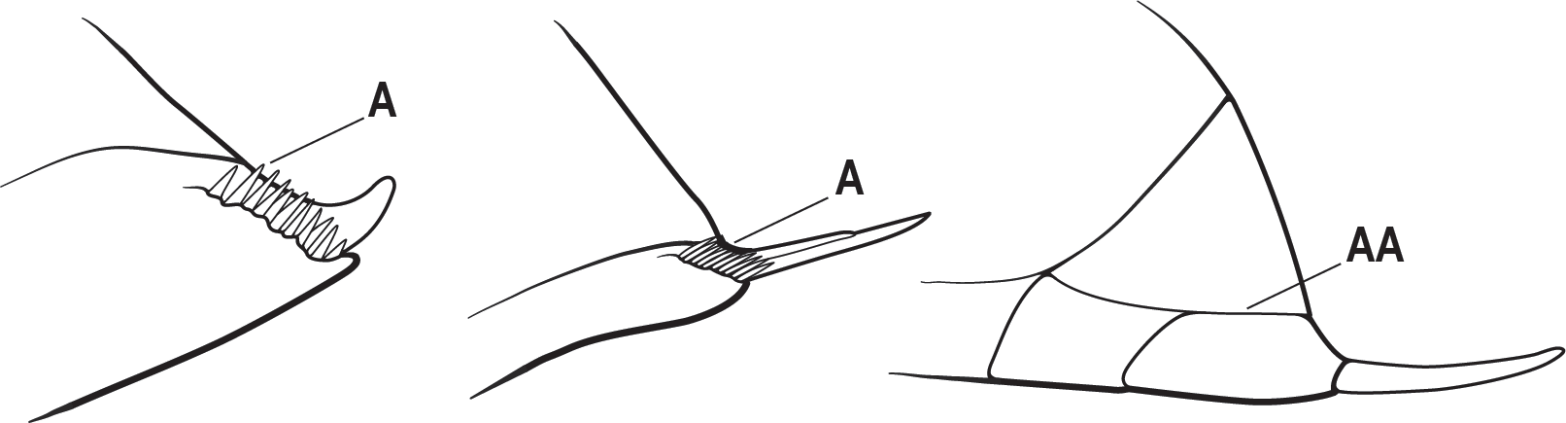		
18(17)	Clypeus with a bluntly rectangular, conspicuously projecting median lobe, the dorsal surface of which is transversely concave (A); metapleural gland orifice followed by a vertical cuticular flange. In dorsal view, base of the first gastral tergite (A3) weakly marginate and distinctly angulate on either side (B); in full-face view, eye located in front of the midlength of the head capsule (C); mandible with basal groove present (D)	*** Paltothyreus ***
–	Clypeus simple, its anterior margin convex, without a rectangular projecting median lobe (AA); metapleural gland orifice not followed by a vertical cuticular flange. In dorsal view, base of the first gastral tergite (A3) not marginate, evenly rounded on either side (BB); in full-face view, eye large, located at or behind the midlength of the head capsule (CC); mandible with basal groove absent (DD)	*** Ophthalmopone ***
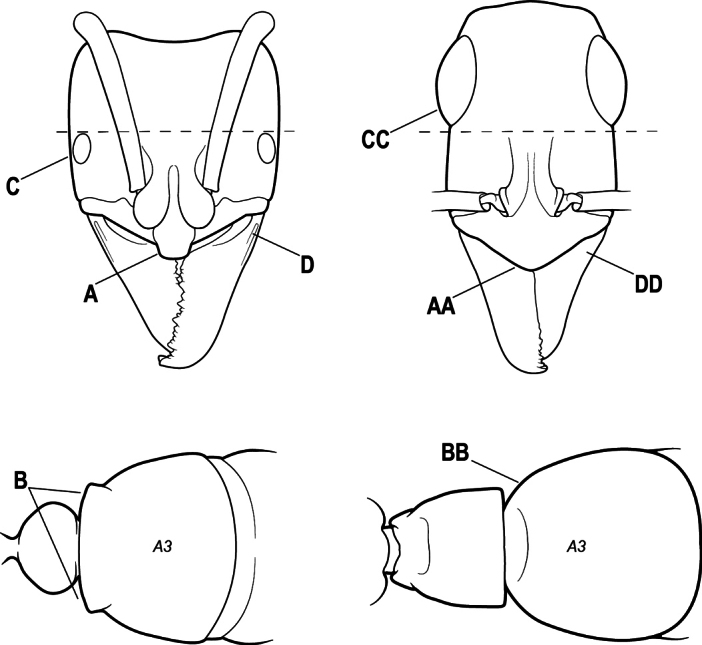		
19(17)	Pretarsal claw of metatarsus usually pectinate, extremely rarely with only 1 or 2 small teeth behind the apex (A); mandible with only 1–3 teeth (usually 2) (B), and frontal lobe distinctly failing to cover the entire antennal socket in full-face view (C)	*** Leptogenys ***
–	Pretarsal claw of metatarsus never pectinate, the claw simple (AA) or at most with teeth confined to the basal 1/3 or less (AAA); if preapical teeth present on claw, then mandible with 4 or more teeth (BB), and frontal lobe fully concealing the antennal socket in full-face view (CC)	**20**
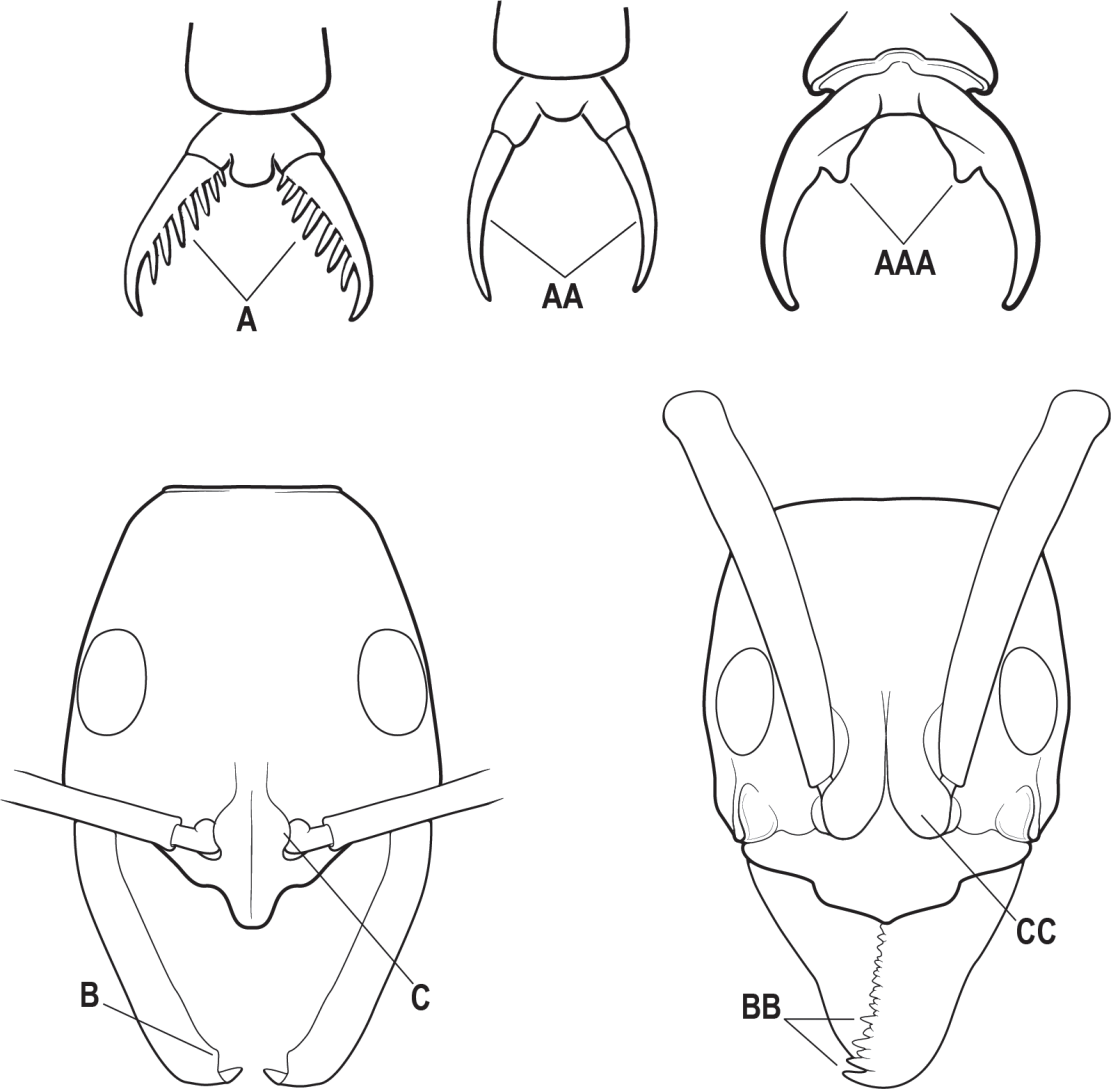		
20(19)	Head with a distinct carina on each side that extends from eye to the clypeal margin (A); polymorphic species, with antennal scape conspicuously flattened (B); posterior portion of clypeus broadly inserted between the frontal lobes (C)	*** Megaponera ***
–	Head without a carina on each side that extends from the anterior margin of the eye to the clypeal margin (AA); monomorphic species, with antennal scape not flattened (BB). Posterior clypeus narrowly inserted between the frontal lobes (CC)	**21**
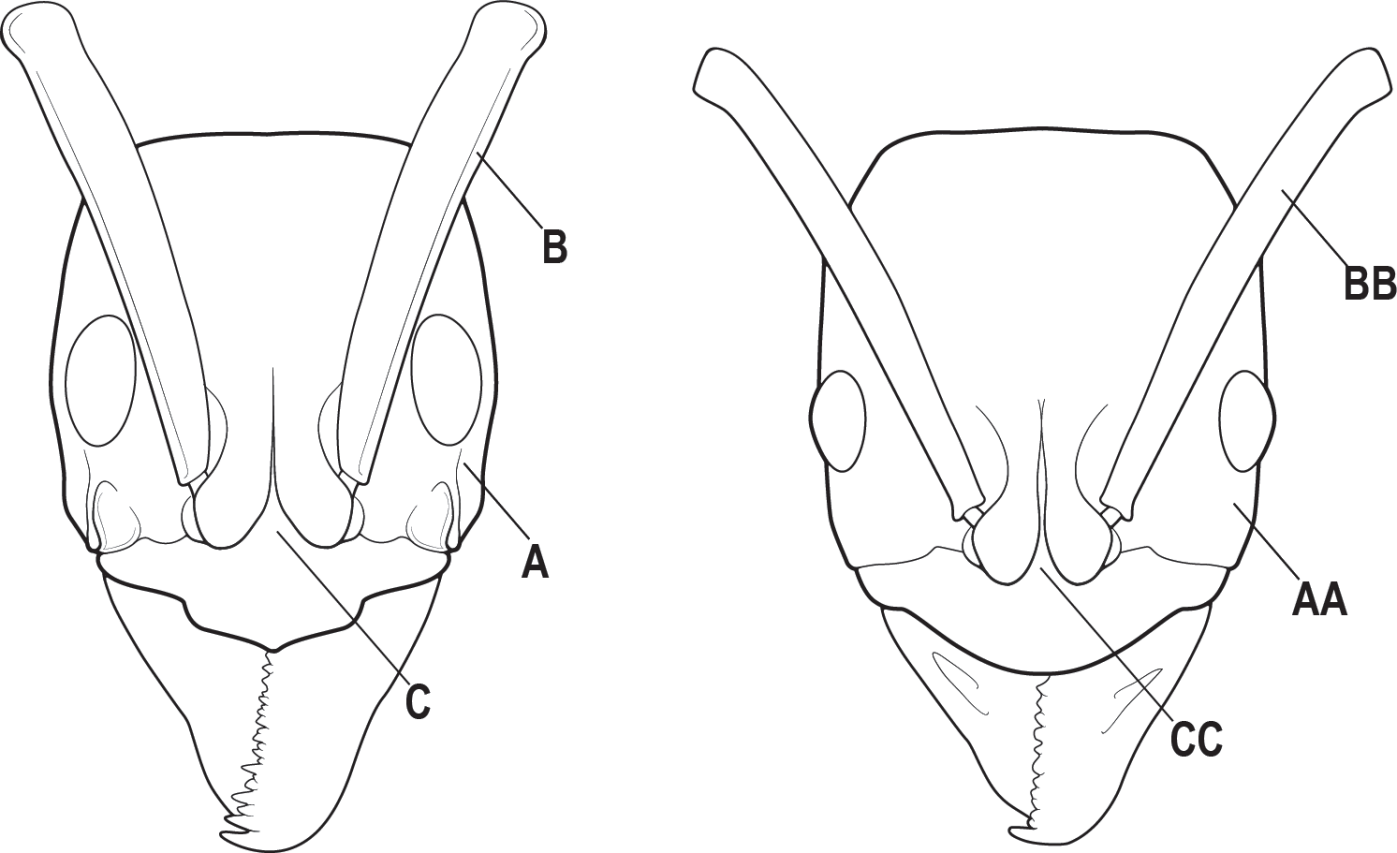		
21(20)	Petiole (A2) dorsally with a comb of five long spines which curve backwards over the base of the first gastral segment (A3) (A); propodeum with a pair of stout spines (B)	*** Phrynoponera ***
–	Petiole (A2) dorsally without a comb of five spines (AA); propodeum unarmed or at most with a pair of small teeth (BB)	**22**
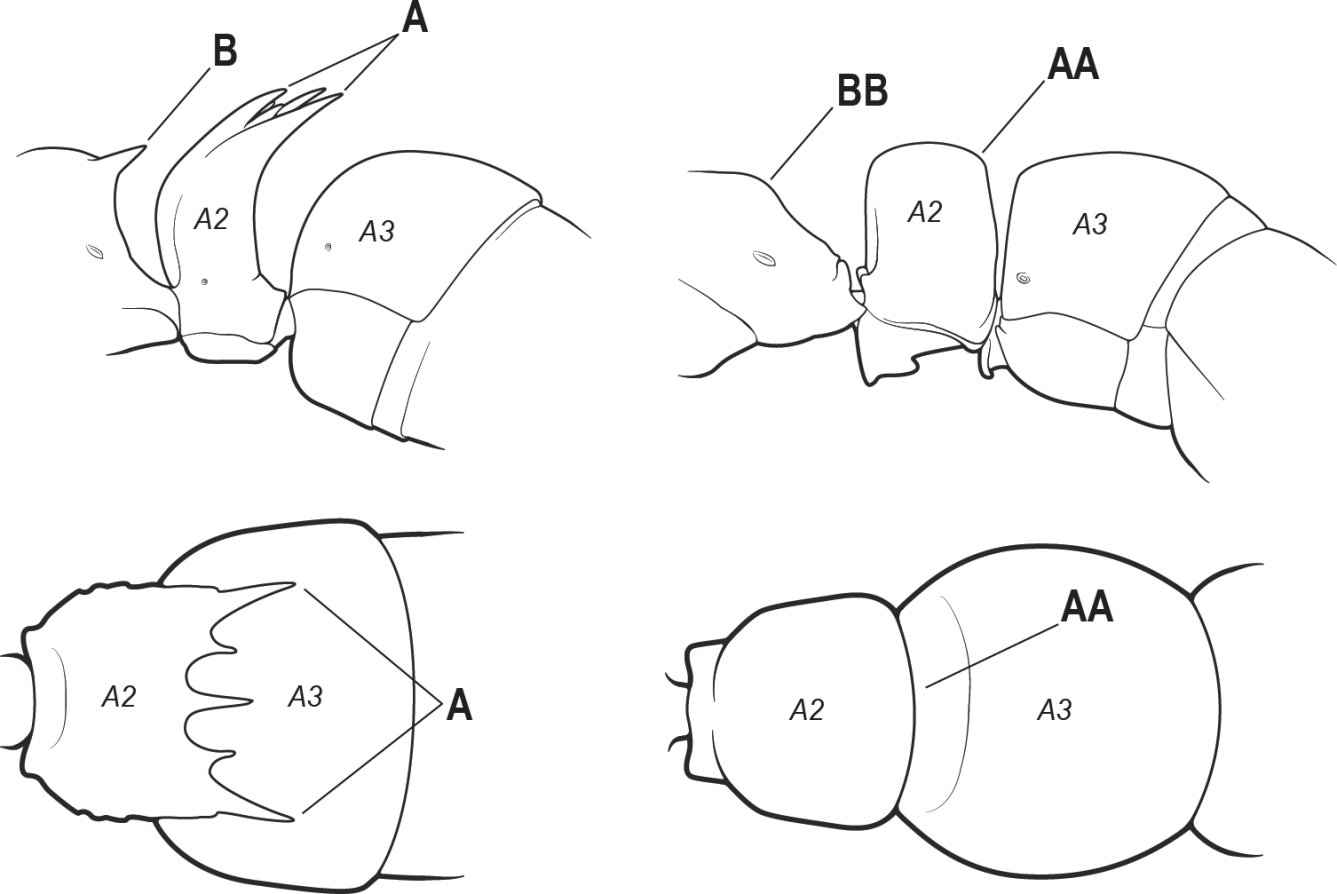		
22(21)	Petiole (A2) with its sides convergent dorsally into a sharp longitudinal crest that runs the length of the segment (A); posterolateral margins of petiole also sharply angulate in the dorsal 1/2, these sharp angles meeting the dorsal crest at its posterior end; anterior clypeal margin broadly concave, the concavity terminating at each side in a prominent angle or tooth-like projection (B); propodeum with a pair of short teeth (C)	*** Streblognathus ***
–	Petiole (A2) scale-like to nodiform but without a sharp longitudinal crest that runs the length of the dorsum (AA); clypeus usually prominent, but if concave, never terminating in prominent angles or teeth (BB); propodeum unarmed (CC)	**23**
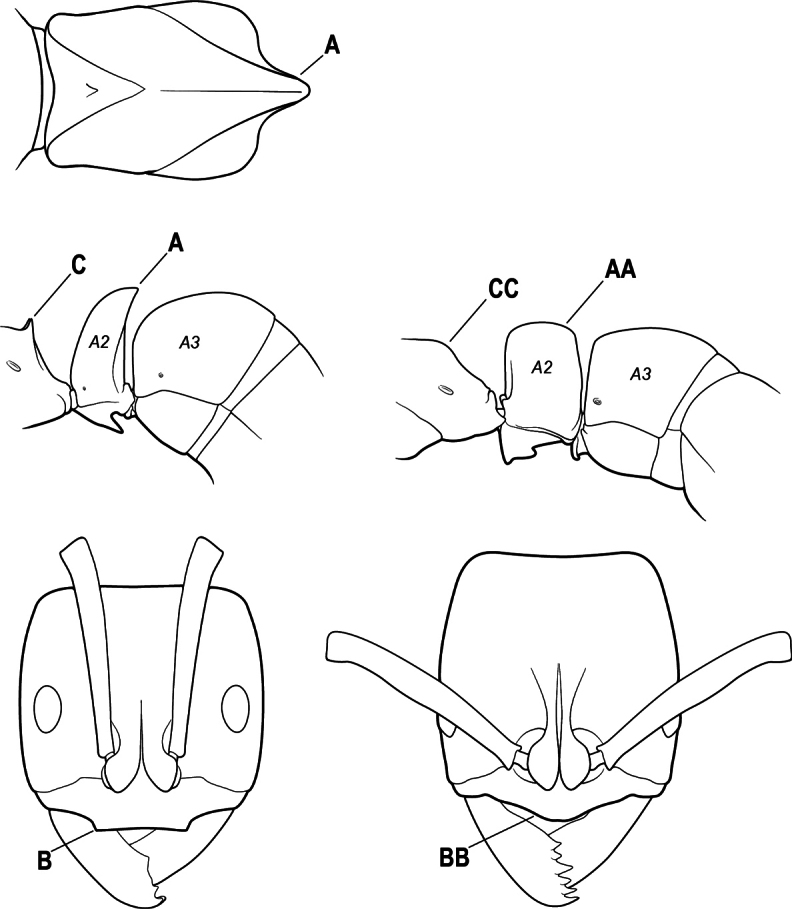		
23(22)	Pretarsal claw of metatarsus with a single, conspicuous preapical tooth on the inner margin (A). Pronotum marginate laterally (B)	*** Hagensia ***
–	Pretarsal claw of metatarsus unarmed (AA), or at most with an inconspicuous basal angle on the inner margin. Pronotum not marginate laterally (BB)	**24**
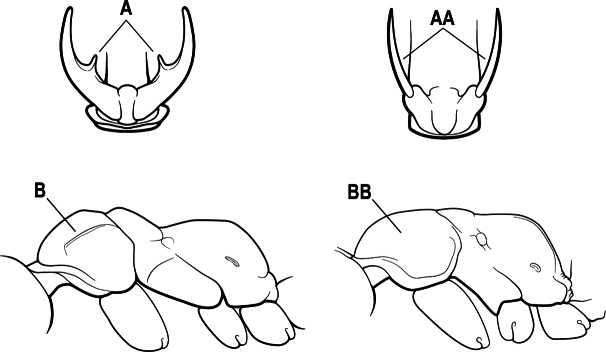		
24(23)	Basal portion of mandible, either with an oblique dorsal groove (A) or with a small circular or near-circular pit or fovea dorsolaterally (AA)	**25**
–	Basal portion of mandible without an oblique dorsal groove or a dorsolateral pit or fovea (AAA)	**27**
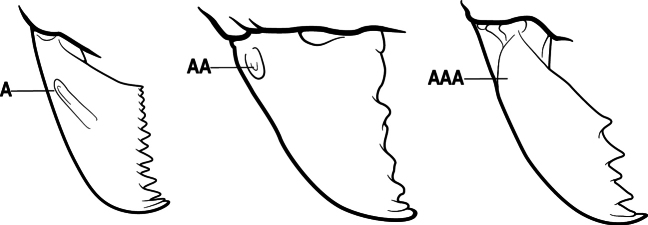		
25(24)	Prora absent from anteroventral angle of first gastral sternite (A3) below the helcium (A). Metanotal groove conspicuous across the dorsal mesosoma (B); in profile, the propodeal dorsum considerably depressed below the level of the mesonotal dorsum (C)	*** Brachyponera ***
–	Prora present at anteroventral angle of first gastral sternite (A3) below the helcium (AA); metanotal groove vestigial to absent across the dorsal mesosoma (BB); in profile, the propodeal dorsum continuing more or less at the same the level as the mesonotal dorsum (CC)	**26**
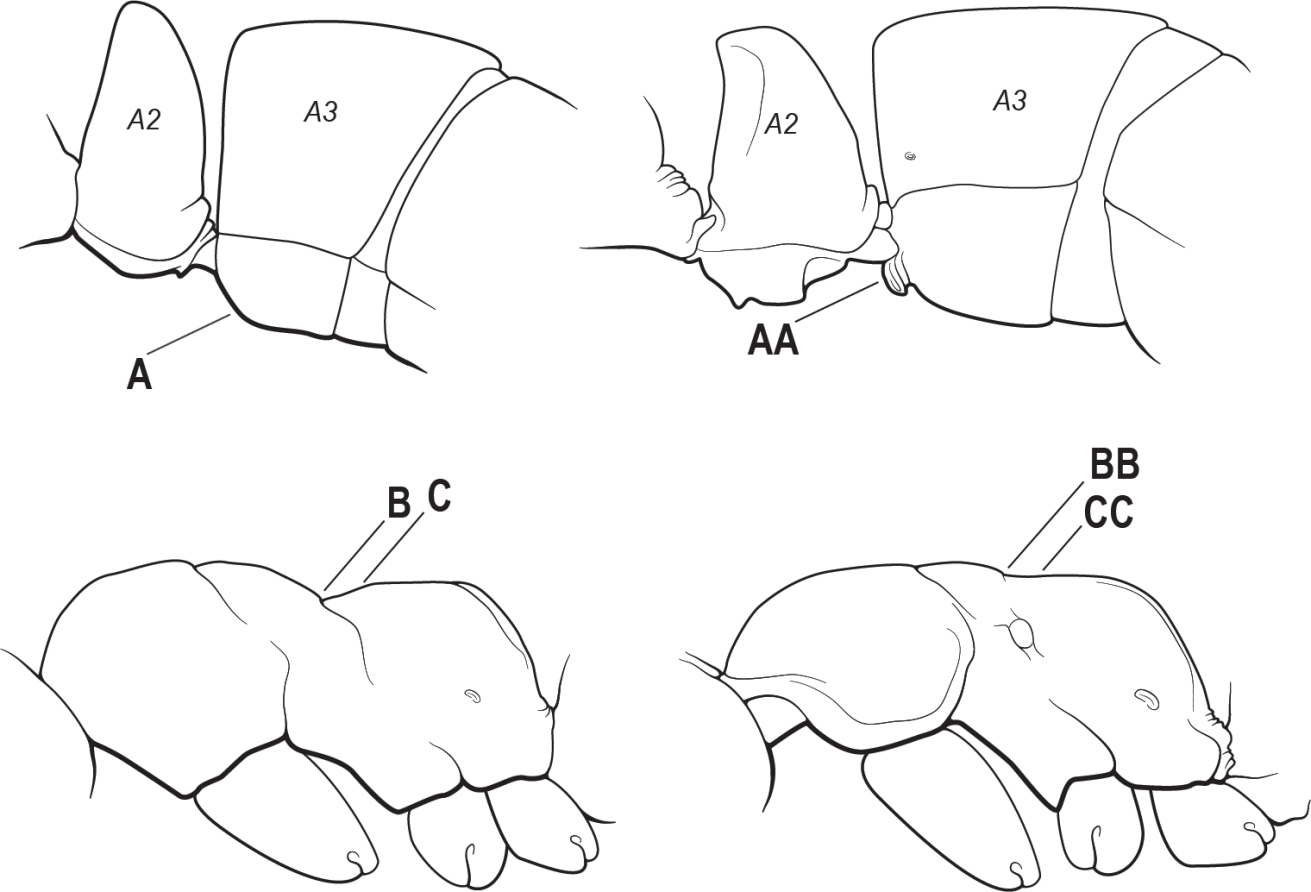		
26(25)	Mesobasitarsus without stout, spine-like setae on outer face (A); metatibial gland present (Africa) or variable (Madagascar); mandible with distinct dorsolateral pit or fovea near the base (B), but without an oblique dorsal groove. (Africa and Madagascar)	*** Euponera ***
–	Mesobasitarsus with stout, spine-like setae on outer face (AA); metatibial gland absent; mandible with an oblique dorsal groove (BB), but without a dorsolateral pit or fovea near the base. (Africa only; absent from Madagascar)	***Fisheropone* (part)**
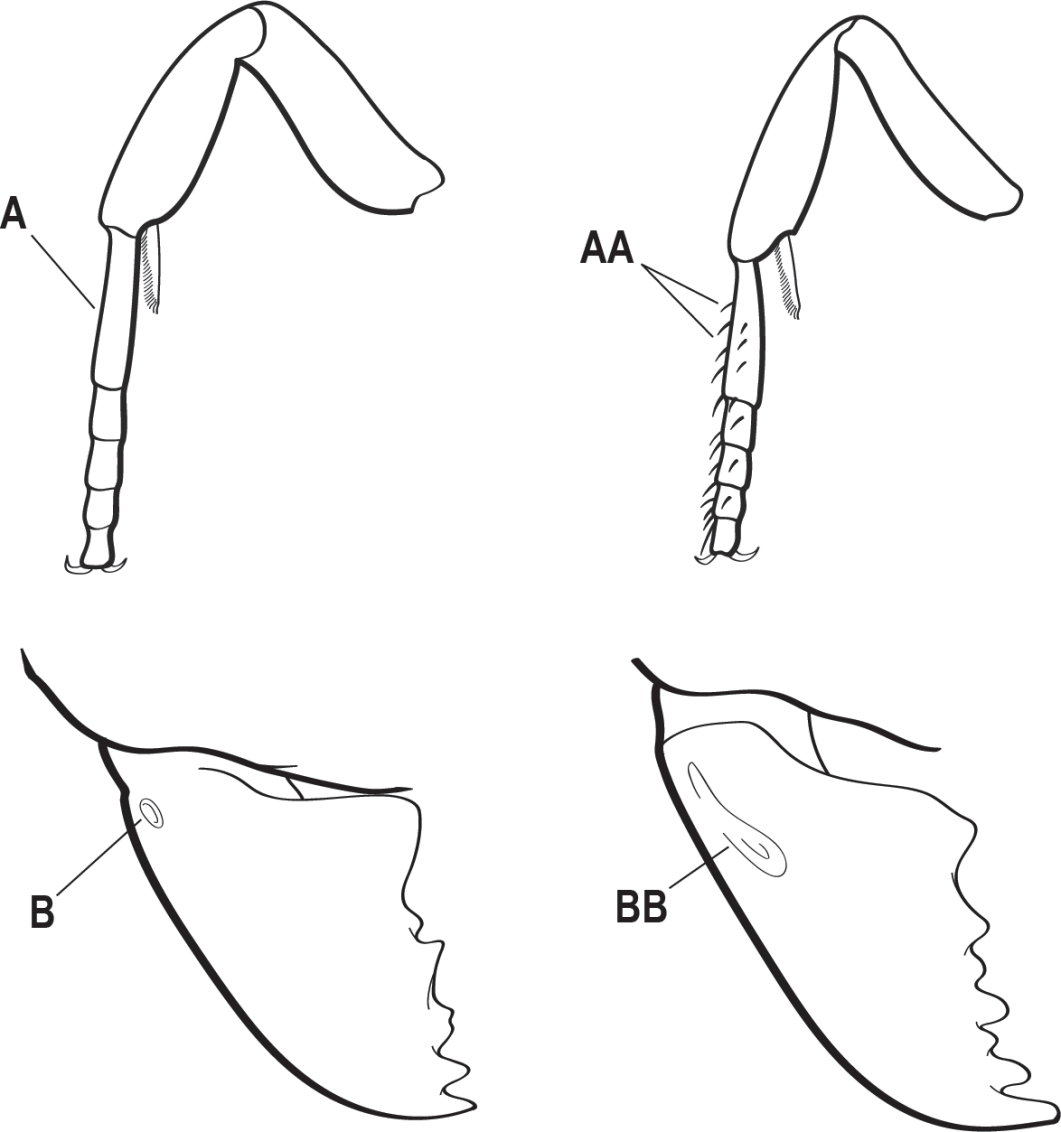		
27(24)	Petiole (A2) block-like in profile (A); metanotal groove usually entirely absent across dorsum of mesosoma (B)	**28**
–	Petiole (A2) generally scale-like (AA); metanotal groove conspicuous across dorsum of mesosoma (BB)	**29**
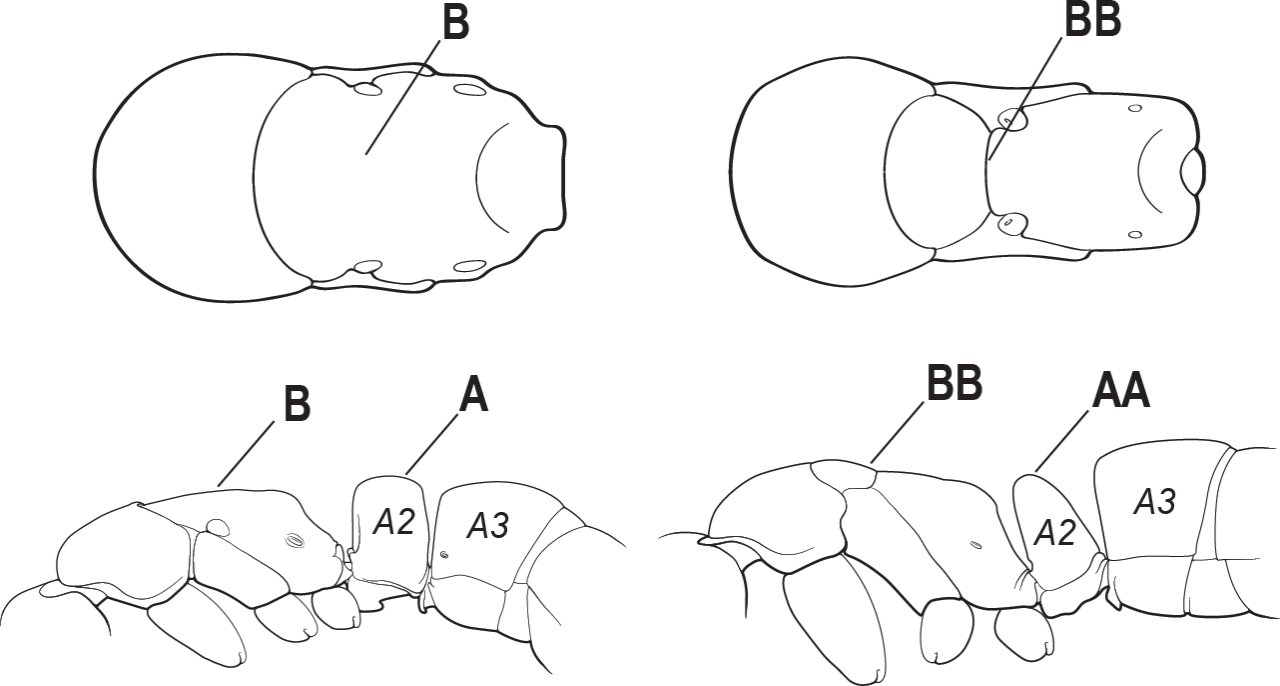		
28(27)	Scape extending at least length of first funicular segment past posterior lateral corner of head (A); mandible with dorsolateral sulcus (B); lower margin of anterior medial area of clypeus convex; frontal lobe subquadrate (C); metatibial gland present (D); tarsal claw simple without basal tooth; abdominal pretergite IV without stridulitrum (E)	*** Boltonopone ***
–	Scape shorter, barely reaching or extending past posterior lateral corner of head by less than length of first funicular segment (AA); mandible without dorsolateral sulcus (BB); lower margin of anterior medial area of clypeus convex, straight, or slightly concave; frontal lobe rounded (CC); metatibial gland absent (DD); tarsal claw with basal tooth; abdominal pretergite IV with stridulitrum (EE)	*** Bothroponera ***
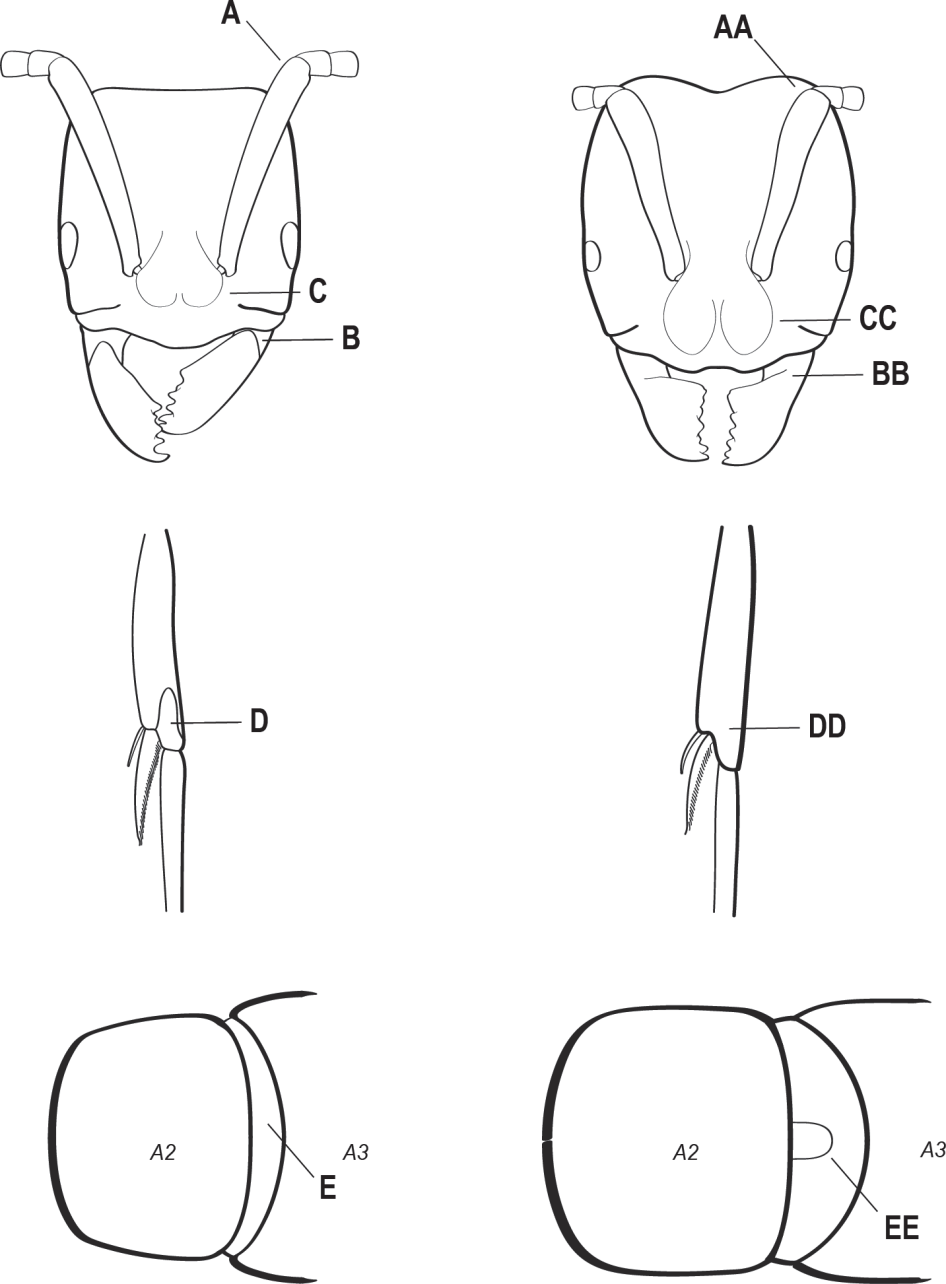		
29(27)	Eye usually absent (A), very rarely a single ommatidium present	**30**
–	Eye always present (AA), with more than three ommatidia	**31**
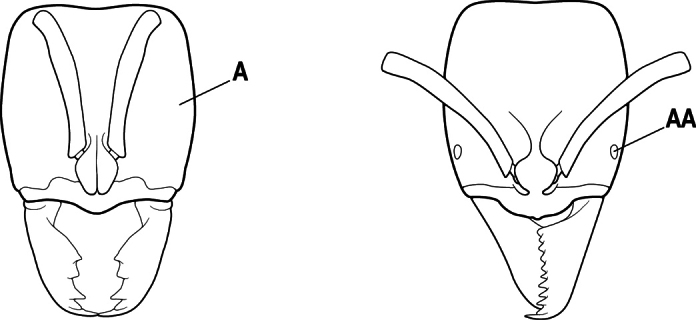		
30(29)	Propodeal spiracle opening slit-shaped (A)	*** Parvaponera ***
–	Propodeal spiracle opening round to oval (AA)	*** Sritoponera ***
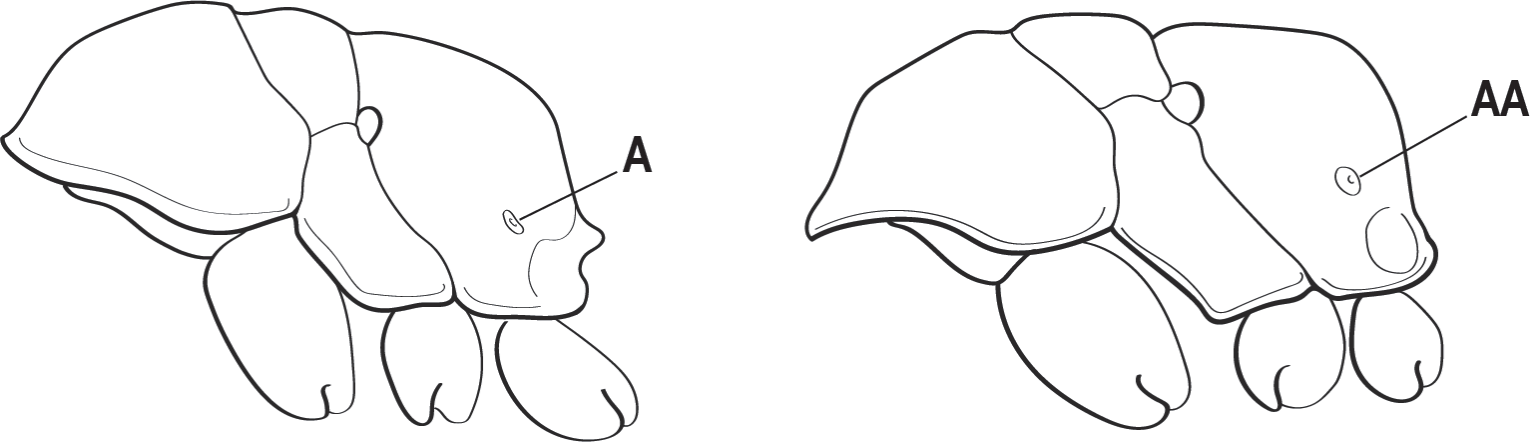		
31(29)	Propodeal spiracle opening slit-shaped (A)	**32**
–	Propodeal spiracle opening round to oval (AA)	**33**
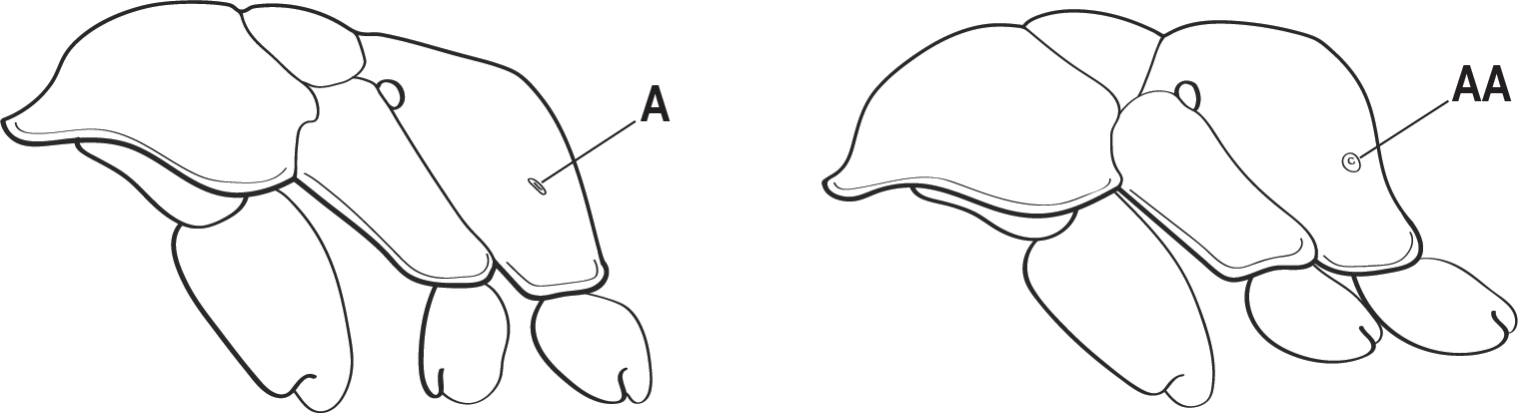		
32(31)	Mandible with teeth along entire masticatory margin (A), mandible without dorsolateral sulcus (B). Antennal scape reaching but not surpassing posterior margin of head (C); mesopleuron without sulcus, not divided into anepisternum and katepisternum (D). Metatibial gland present (E), apical 1/2 of ventral surface of metatibia with an elongate impression or area of extremely thin translucent cuticle; metabasitarsus with stout, spine-like setae on outer face (F); abdominal segment IV with constriction between presclerites and postsclerites	*** Makebapone ***
–	Mandible with basal 1/3 of masticatory margin edentate (AA) and teeth restricted to apical portion, weak dorsolateral sulcus at the base of the mandible (BB); antennal scape distinctly surpassing posterior lateral margin of head (CC); mesopleuron divided into anepisternum and katepisternum (DD). Metatibial gland absent (EE); metabasitarsus without stout, spine-like setae on outer face (FF); abdominal segment IV without constriction between presclerites and postsclerites	*** Subiridopone ***
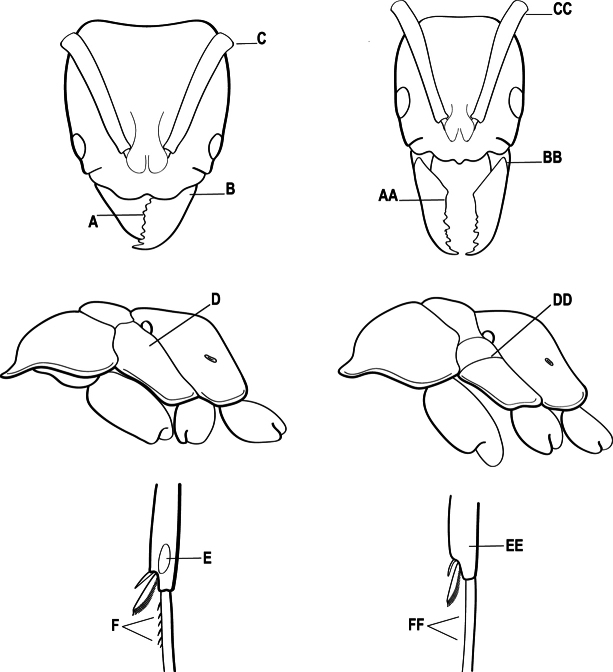		
33(31)	Length of first funicular segment distinctly longer than the second (A); anterior margin of clypeus usually bearing a tooth-like projection at the midpoint (B)	*** Xiphopelta ***
–	Length of first funicular segment shorter than the second funicular (AA); anterior margin of clypeus without tooth-like projection at midpoint (BB) (Seychelles)	***Mesoponera* (*M. melanaria*)**
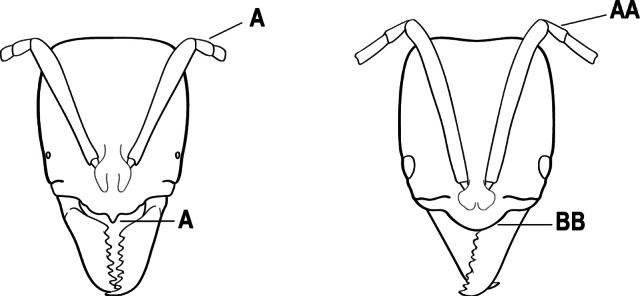		

#### Key to Palearctic–Indomalaya–Australasia ponerine ant genera (workers) including all of Europe, Asia, Australia, Melanesia, and Polynesia

**Table:** 

1	Clypeus broadly inserted between frontal lobes, which appear flattened in frontal view (A), the antennal sockets widely separated (B); metatibia with two pectinate spurs (C); tarsal claw usually armed with a single preapical tooth (rarely unarmed); petiole usually attached at approximately mid-height of anterior face of first gastral segment (D) (attached low on the anterior face in Australian species); sculpturing usually uniformly pruinose (less pronounced in Australian species)	*** Platythyrea ***
–	Clypeus narrowly inserted between frontal lobes (AA), antennal sockets closely approximated (BB); metatibia with either one or two spurs; if the latter, then either one spur is pectinate and the other simple (CC) and tarsal claw unarmed, or the second spur is also pectinate and the tarsal claw is pectinate; petiole usually attached low on the anterior face of the first gastral segment (DD); sculpturing rarely uniformly pruinose	**2**
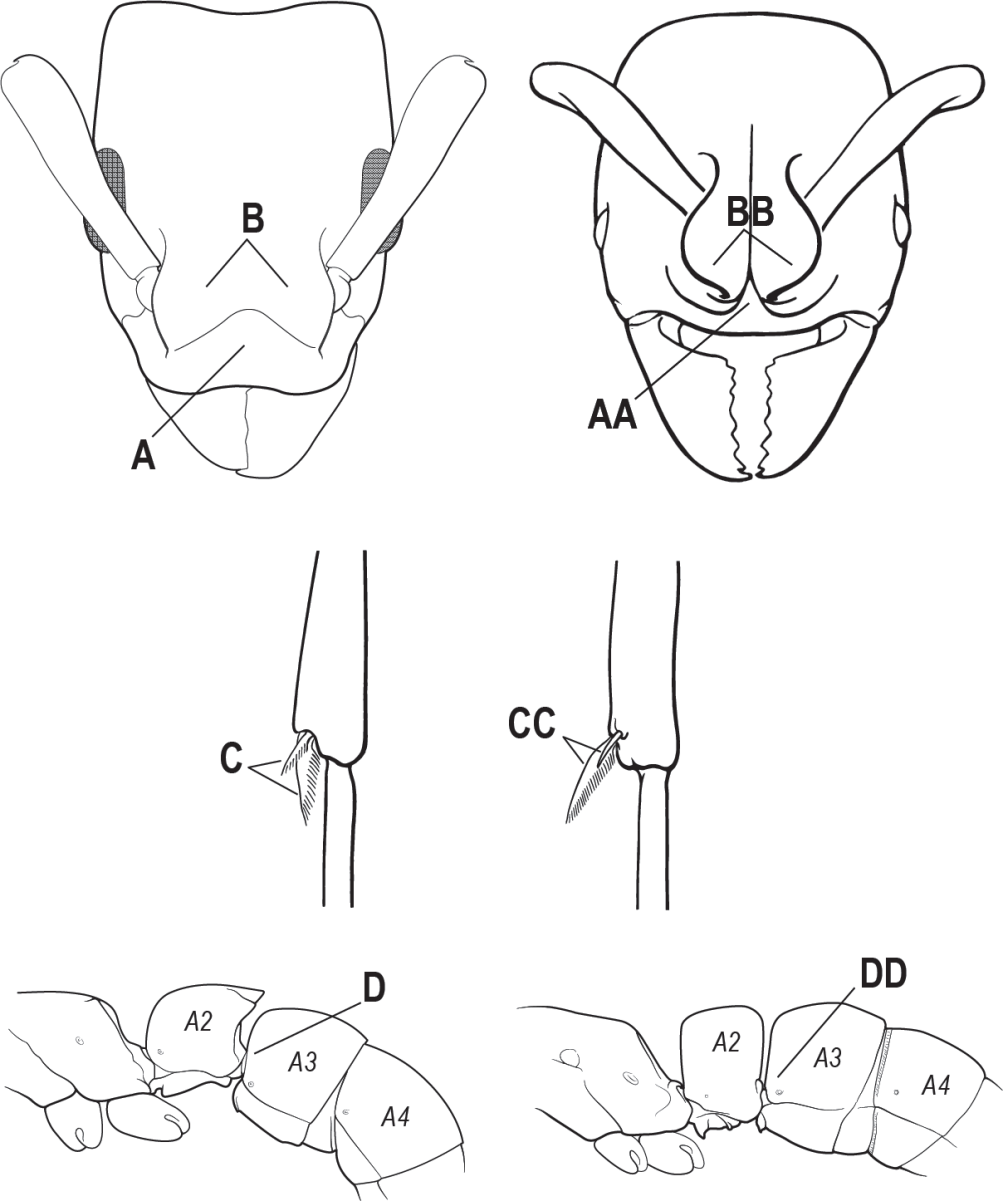		
2(1)	Mandible long and linear, inserted medially on the front of the head (A); head with well-developed ocular prominences (B)	**3**
–	Mandible inserted toward the sides of the front of the head (AA); head without well-developed ocular prominences (BB)	**4**
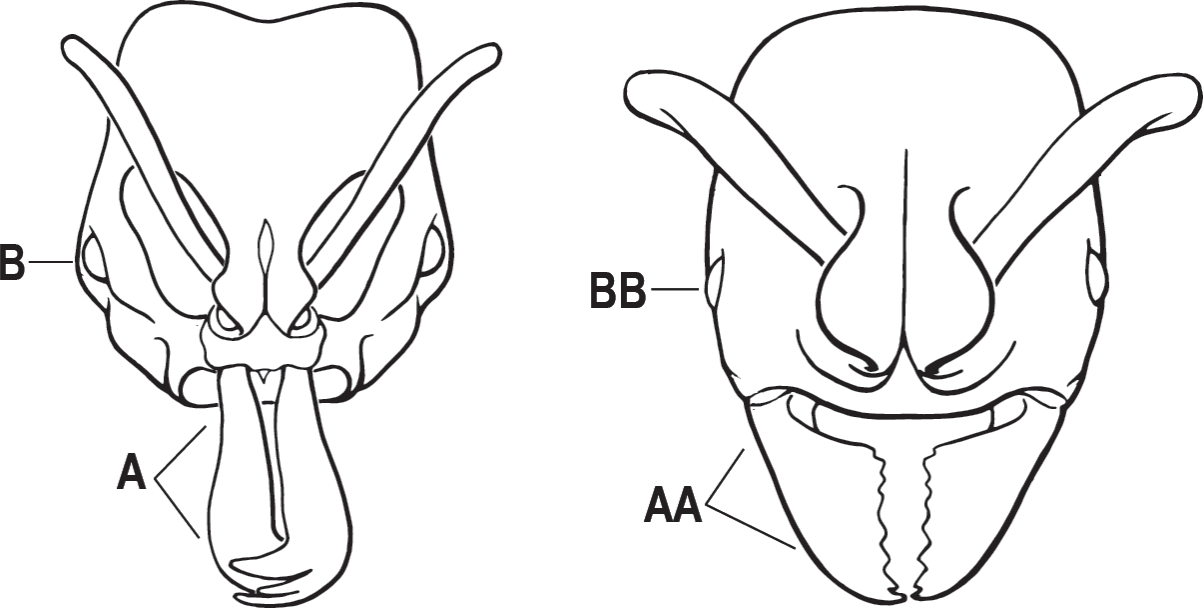		
3(2)	Nuchal carina (which separates dorsal from posterior surfaces of head) converging in a V-shape at the midline (A) and also receiving a pair of prominent dark posterior apophyseal lines that converge to form the sharp median-dorsal groove of the vertex (B); dorsalmost tooth of apical mandibular series truncated (C)	*** Odontomachus ***
–	Nuchal carina forming a broad, uninterrupted curve across the posterodorsal extremity of the head (AA); posterior surface without paired dark apophyseal lines; on vertex, the median groove absent or ill-defined and shallow (BB); dorsalmost tooth of apical mandibular series acute (CC)	*** Anochetus ***
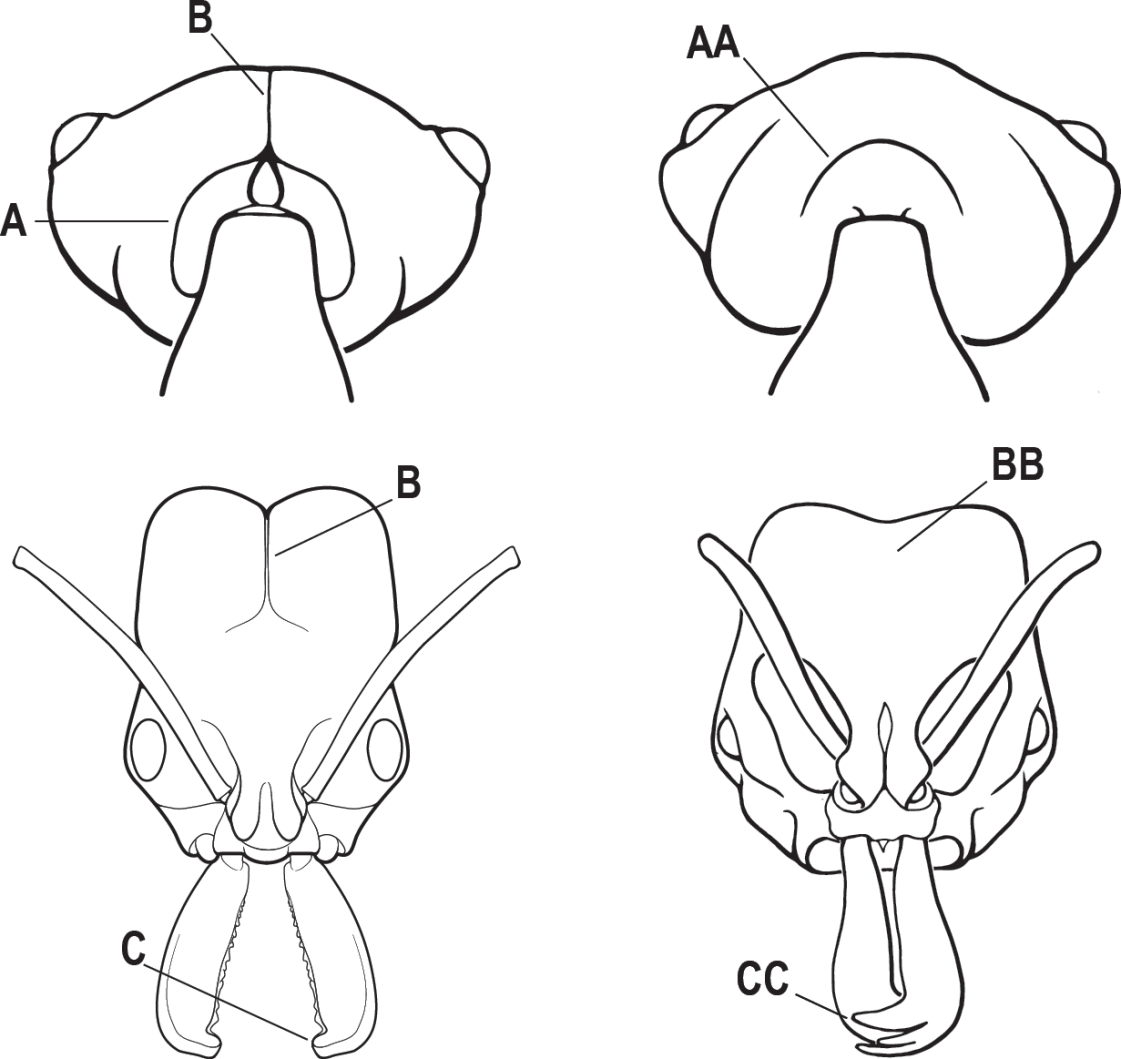		
4(2)	Mesotibia dorsally with abundant stout traction setae (A)	**5**
–	Mesotibia dorsally without abundant stout traction setae (a few stout setae sometimes present near tarsus but never extending along length of tibia (AA)	**6**
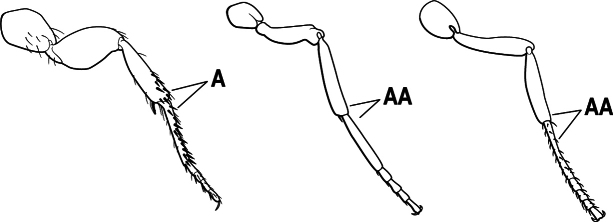		
5(4)	Head and body without a dense pubescence; mandible with a lateral longitudinal groove (A) and without a basal pit; metapleural gland orifice opening laterally (B)	*** Centromyrmex ***
–	Head and body covered by a dense pubescence; mandible with a basal pit (AA) and without a lateral longitudinal groove; metapleural gland orifice opening posteriorly at the posteroventral corner of the metapleuron (BB)	***Cryptopone* (part)**
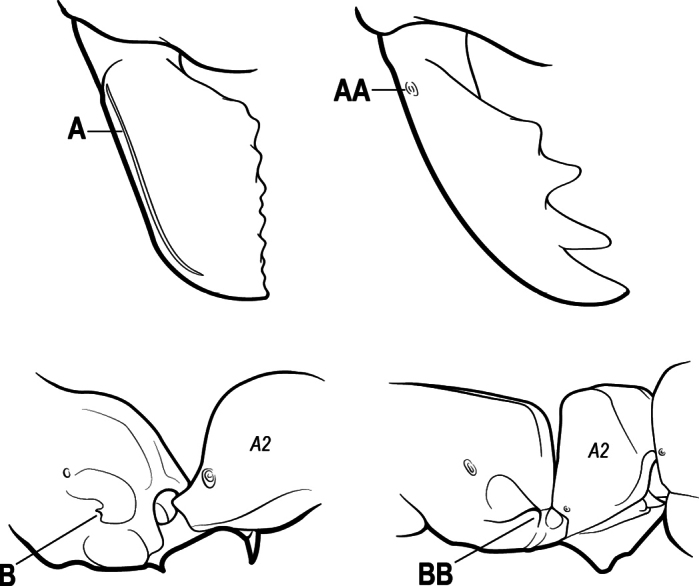		
6(4)	Ventral apex of the metatibia with a single spur, which is pectinate (A)	**7**
–	Ventral apex of the metatibia with both a large pectinate spur and a smaller simple (AA) or shallowly pectinate spur	**10**
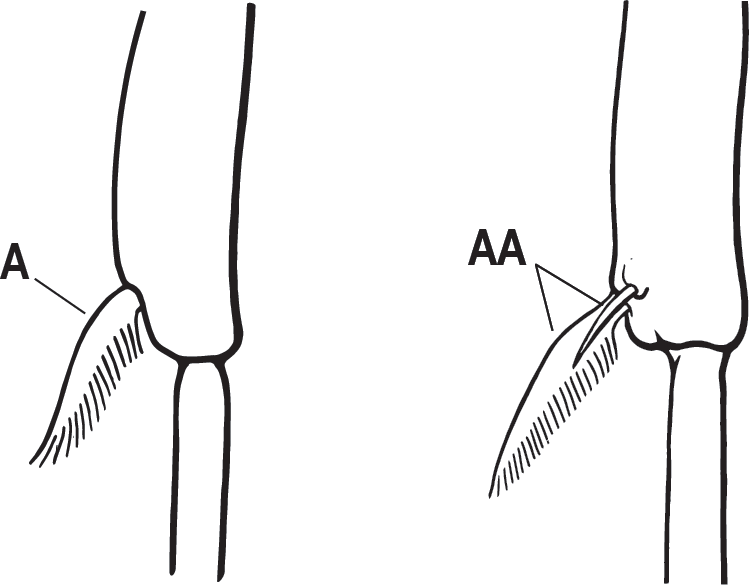		
7(6)	Eye absent (A); mandible short and narrow (B); if mandible triangular, then with dorsal lateral pit (C)	***Cryptopone* (part)**
–	Eye present (AA) (although sometimes very small); mandible generally triangular but occasionally elongate (BB); mandible without dorsal lateral pit (CC)	**8**
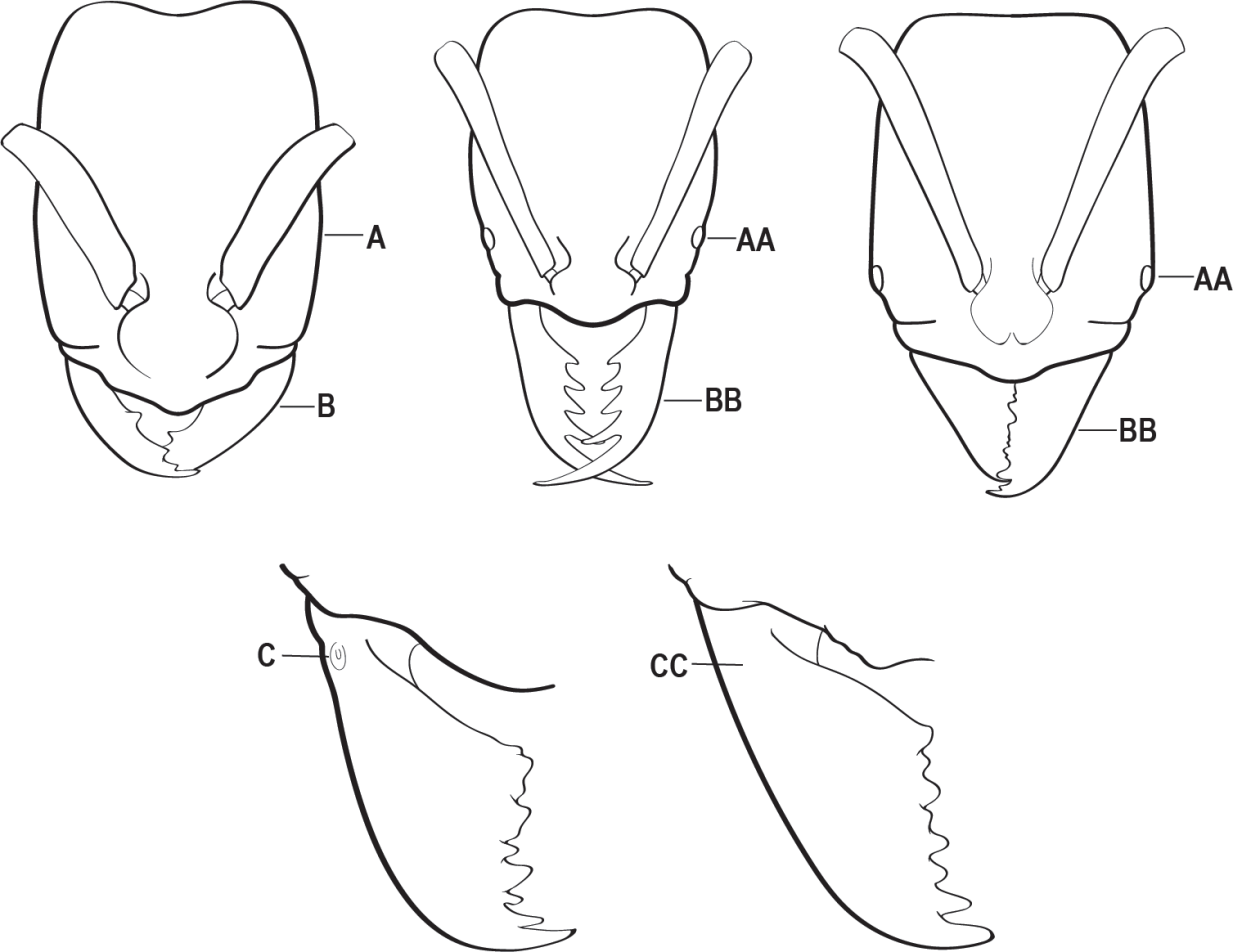		
8(7)	Mandible thin, with long attenuated teeth (A)	*** Emeryopone ***
–	Mandible linear or subtriangular or triangular, the teeth not long and not attenuated (AA)	**9**
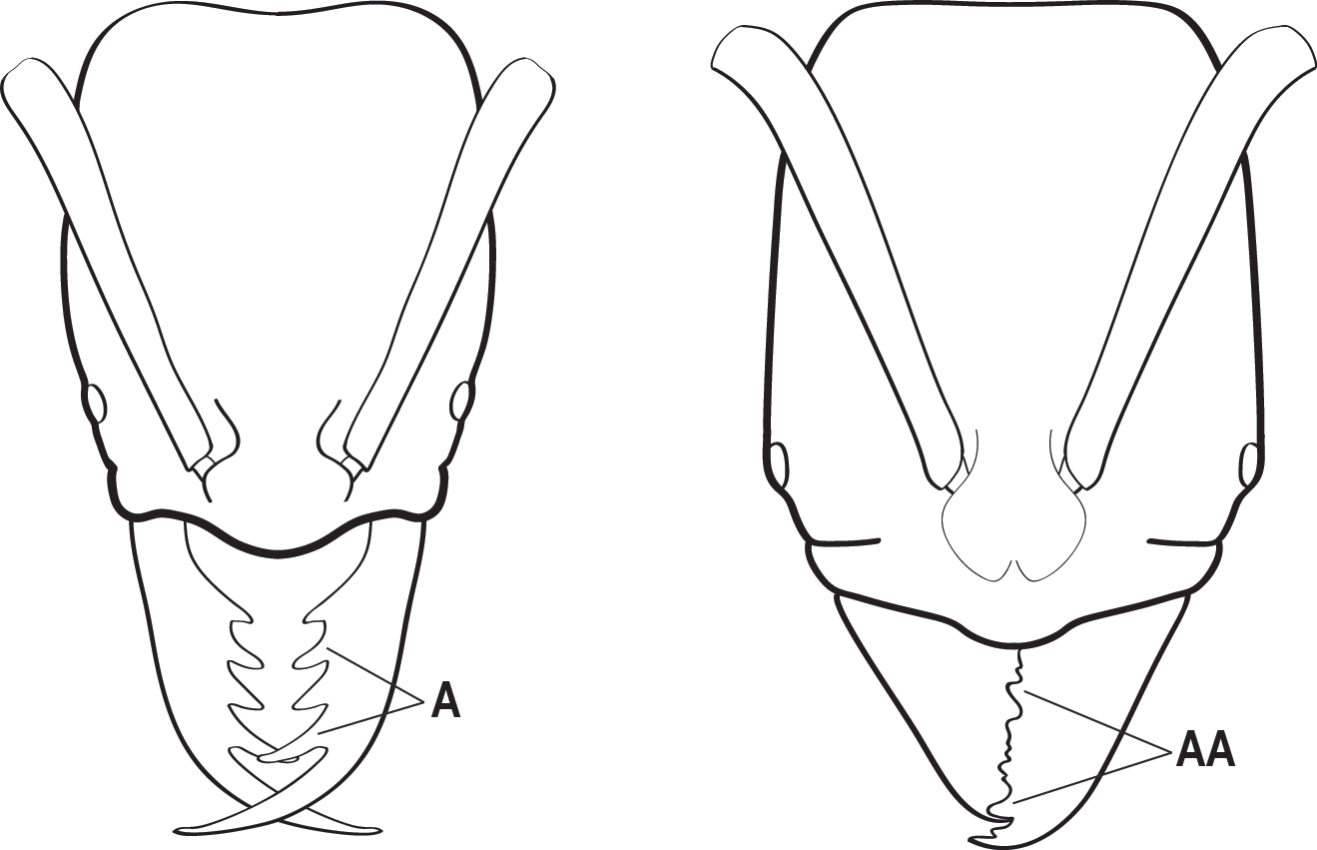		
9(8)	Subpetiolar process with an anterior fenestra (A) and paired posteroventral teeth (B)	*** Ponera ***
–	Subpetiolar process without an anterior fenestra (AA) and without paired posteroventral teeth (BB)	*** Hypoponera ***
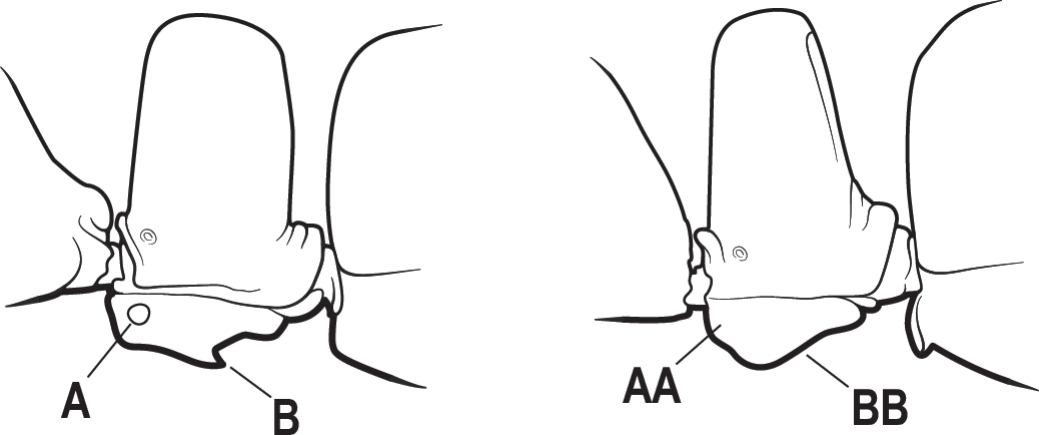		
10(6)	Tarsal claw pectinate or armed with one or two preapical teeth (A)	**11**
–	Tarsal claw unarmed (AA)	**12**
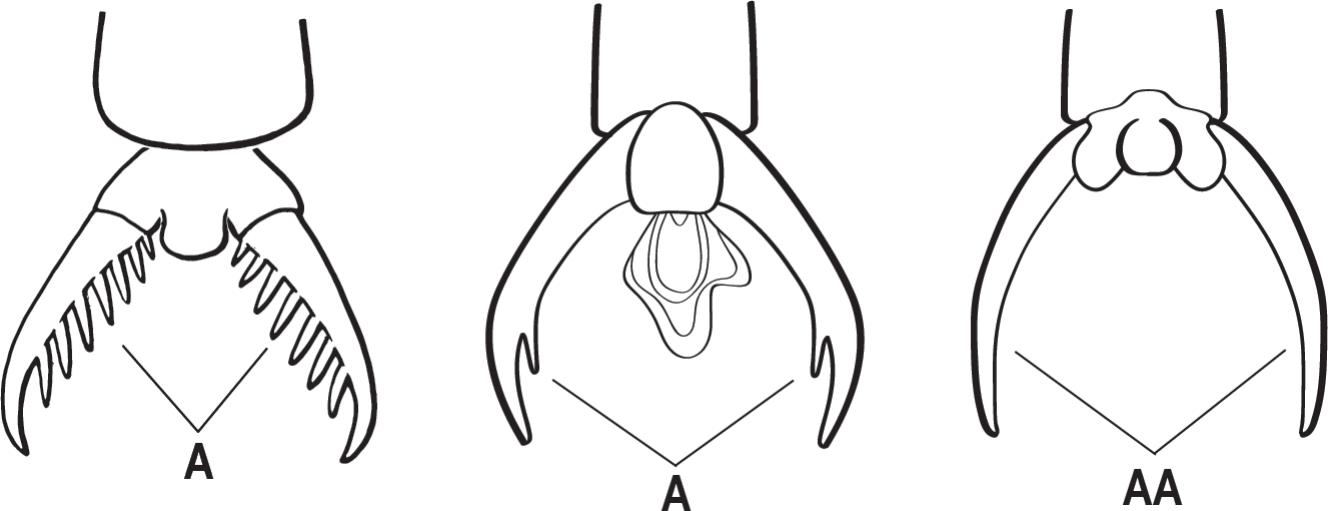		
11(10)	Eye extremely large and located at the extreme anterior end of the head (A); mandible long, narrow, upcurved and parallel, with two rows of small teeth on the masticatory margins (B); tarsal claw armed with a single preapical tooth (C); arolia prominent and white	*** Harpegnathos ***
–	Eye variable in size, but not extremely large, and typically located at or near the midline of the head (AA); mandible triangular or thin and curved (BB); tarsal claw usually pectinate (CC), sometimes with only one to three preapical teeth; arolia not prominent and white	*** Leptogenys ***
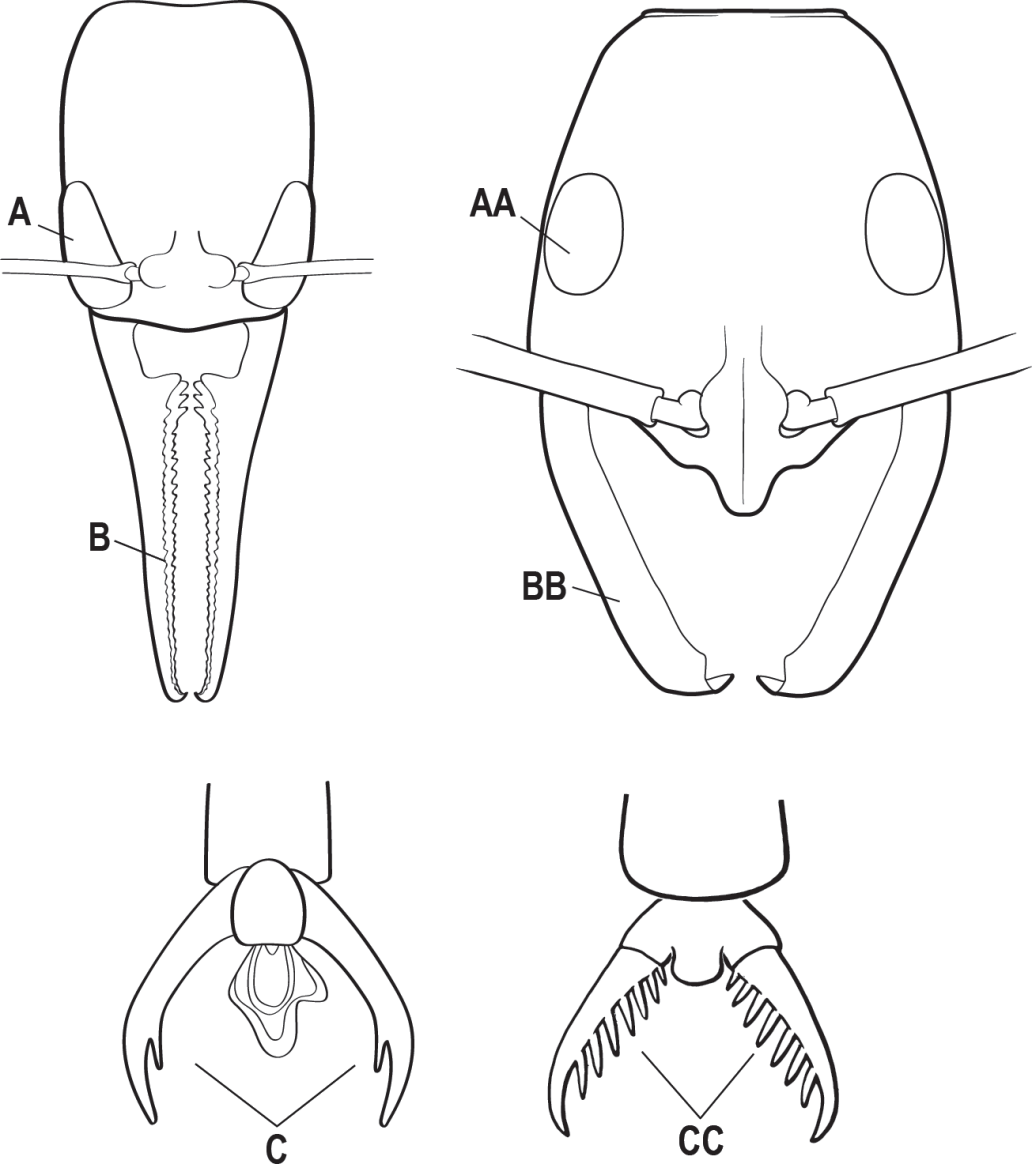		
12(10)	Propodeal spiracle opening slit-shaped (A)	**13**
–	Propodeal spiracle opening round or ovoid (AA)	**20**
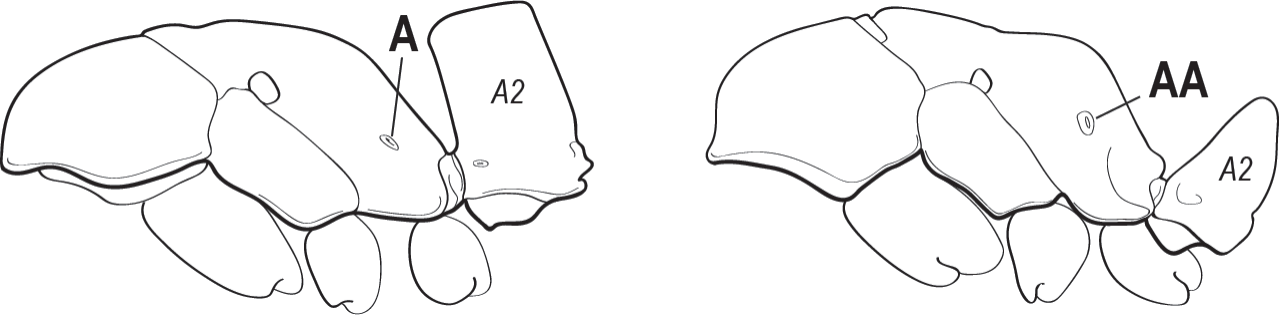		
13(12)	Mesosoma with a deep pit anterior to the metanotal spiracle (A); petiole with a pair of spines on the posterodorsal margin (B); arolia prominent and white	*** Diacamma ***
–	Mesosoma without deep pits laterally (AA); petiole rounded dorsally, without a pair of spines (BB); arolia not prominent and white	**14**
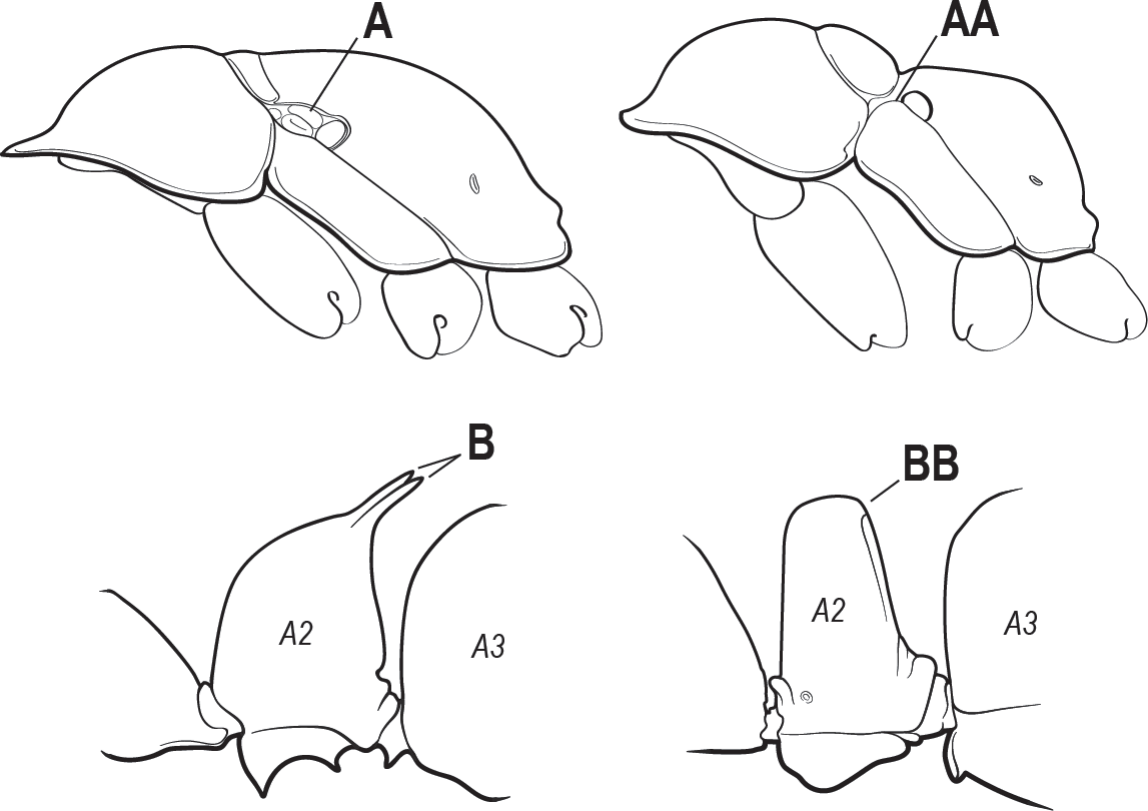		
14(13)	Compound eye small (with 2–4 facets) or absent (A); subpetiolar process a rounded lobe without posterior angle or teeth; petiolar node scale-like	**15**
–	Compound eye usually larger, with numerous facets (AA); subpetiolar process a rounded lobe or with posterior angle or teeth; petiolar node scale-like or subquadrate	**16**
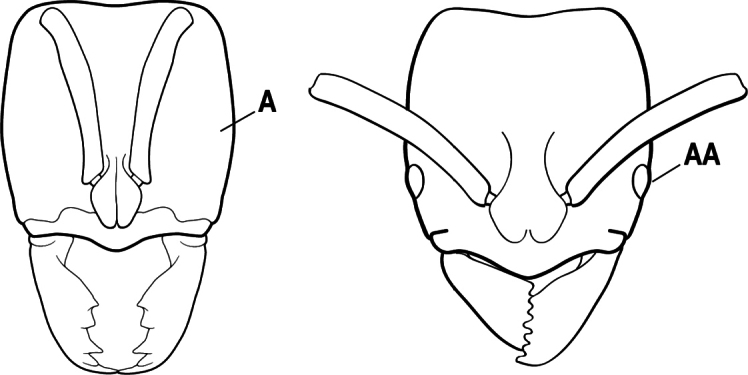		
15(14)	Subpetiolar process without an anterior fenestra (A); color dark brown	*** Fisheropone ***
–	Subpetiolar process with an anterior fenestra (AA); color light yellow brown	*** Parvaponera ***
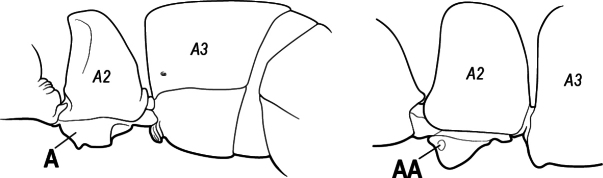		
16(15)	In frontal view, eye located dorsally on head and separated from the lateral margin by a small gap (A); head and body usually with both dense pilosity and dense pubescence (B); propodeum broad dorsally; petiole semicircular in dorsal profile, often with a row of small teeth or denticles on the posterodorsal margin (C) (angled or ridged in some Australian species)	*** Pseudoneoponera ***
–	In frontal view, eye usually located laterally on head and touching or extending beyond the lateral margin (AA); head and body with less pilosity or less pubescence (BB); petiole semicircular in dorsal profile without a row of small teeth or denticles on the posterodorsal margin (CC)	**17**
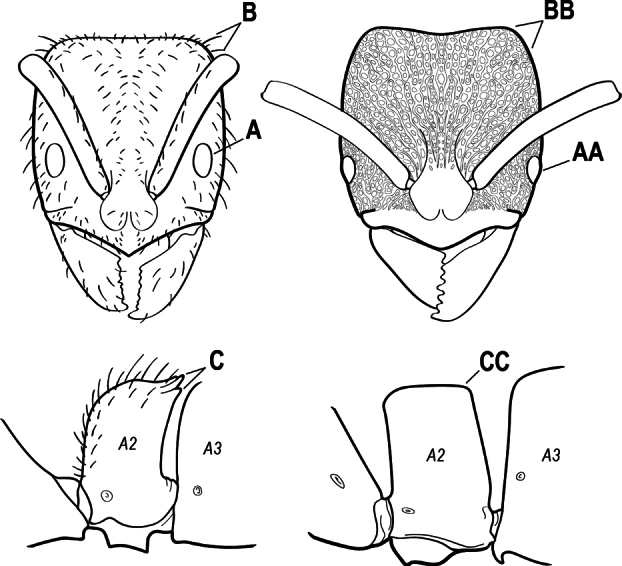		
17(16)	Head and body strongly sculptured (usually striate); posterolateral margins of head angular (A); mesopleuron divided by a transverse groove (B); petiolar node in dorsal view generally convex anteriorly and flat posteriorly (a few exceptions)	*** Ectomomyrmex ***
–	Head and body not strongly sculptured; posterolateral margins of head rounded (AA); mesopleuron not divided (BB); petiolar node scale-like or block-like	**18**
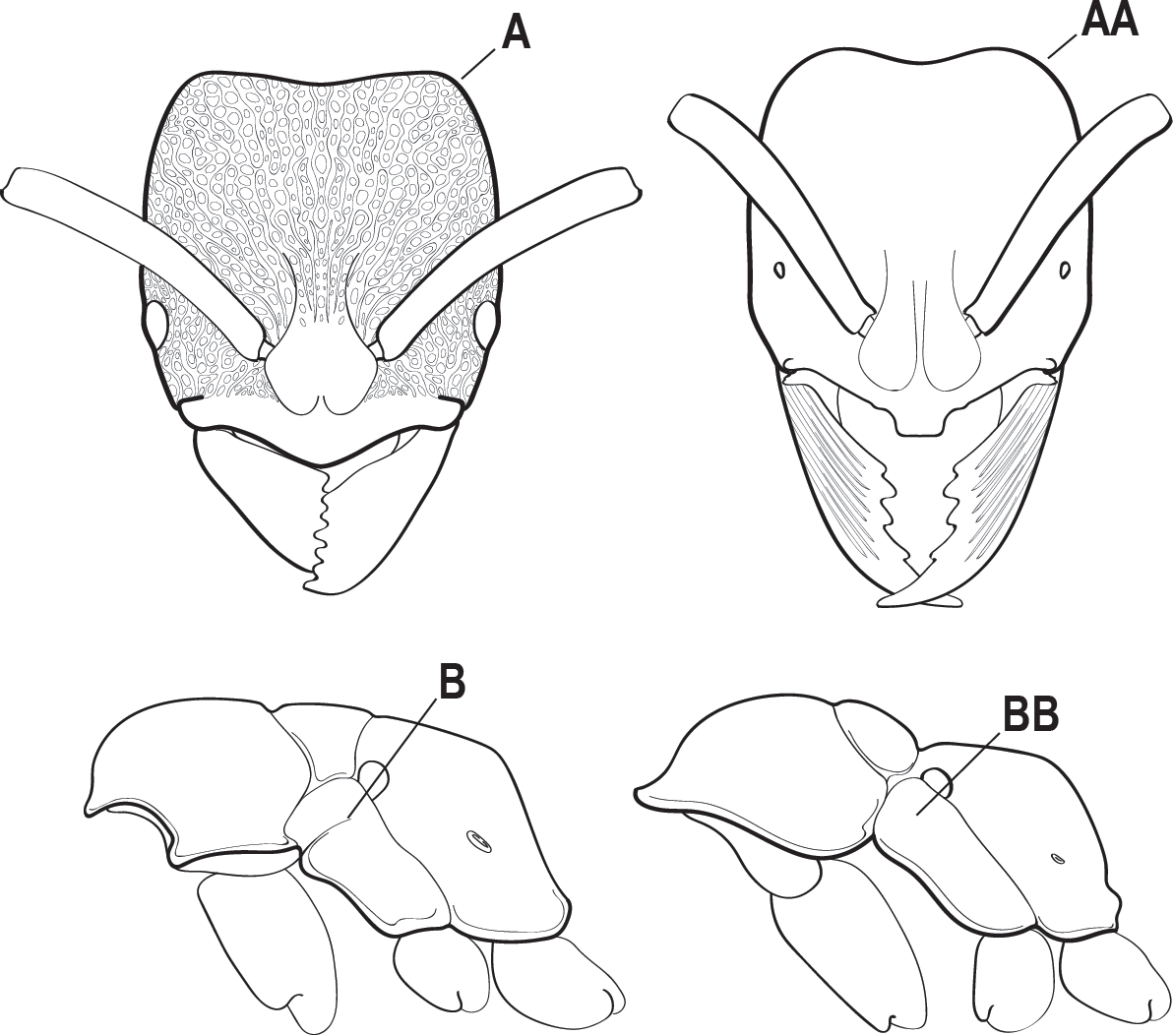		
18(17)	Mesosoma without constriction at the anterior portion of the propodeum, propodeum more or less block-like in dorsal view (A); metanotal groove absent or essentially absent (B); petiole generally block-like in profile	*** Boltonopone ***
–	Mesosoma with constriction at the anterior portion of the propodeum, dorsal surface of propodeum rectangular in dorsal view, visible (AA); metanotal groove present as a shallow impression (BB); petiole generally scale-like or block-like	**19**
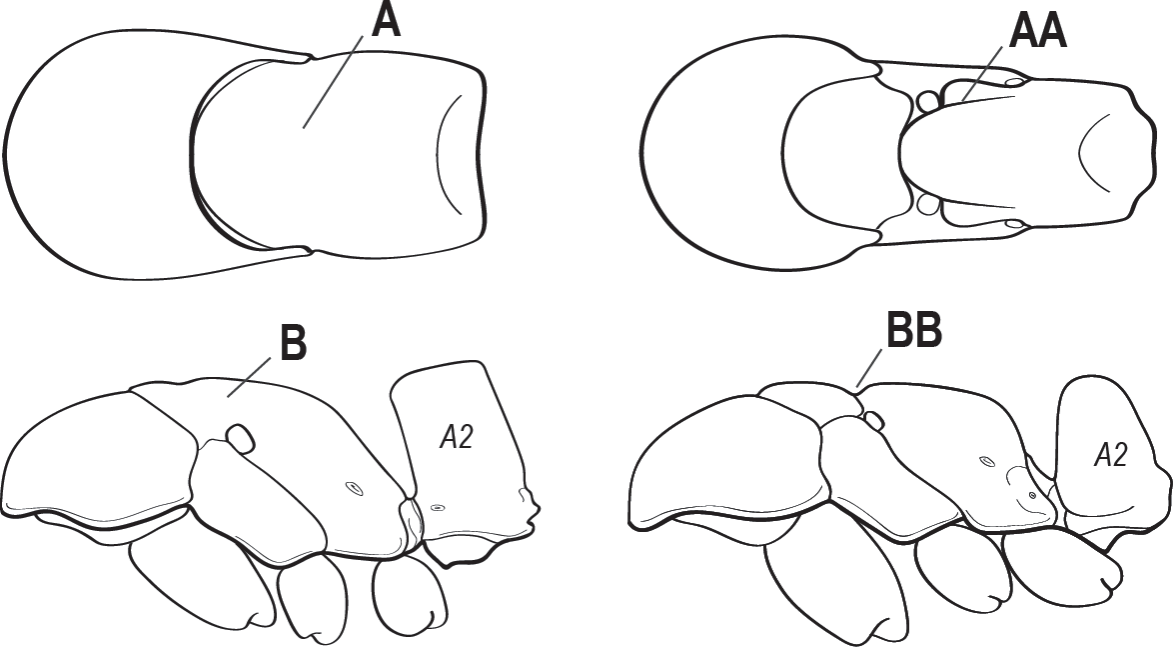		
19(18)	Petiole (A2) block-like in profile (A); subpetiolar process with sharply angulate posterior margin (B)	***Austroponera* (part, *A. pachynoda*)**
–	Petiole (A2) generally scale-like (AA); subpetiolar process rounded posteriorly (BB)	*** Pseudoponera ***
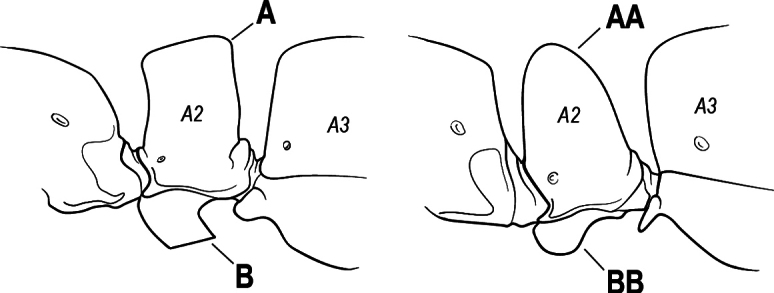		
20(12)	Mandible linear or subtriangular (A); clypeus with a bluntly rectangular, conspicuously projecting median lobe (B), the dorsal surface of which is transversely concave	**21**
–	Mandible triangular (AA); clypeus simple, its anterior margin convex, without a rectangular projecting median lobe (BB)	**22**
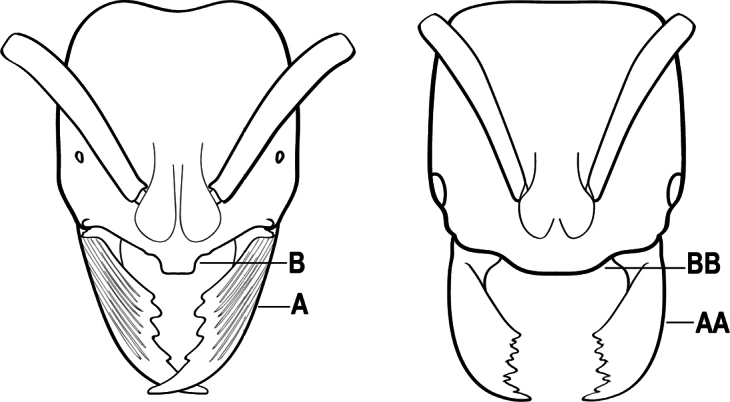		
21(20)	Eye extremely small (A); mandible subtriangular with distinct longitudinal sculpture (B); metanotal suture obsolete dorsally (C); petiole a thick scale (D); gaster with only a moderate girdling constriction (E)	*** Buniapone ***
–	Eye small to moderate (AA); mandible linear, completely unsculptured (BB); metanotal suture distinct dorsally (CC); petiole with a small but thick node (DD); gaster with a strong girdling constriction (EE)	***Myopias* (part)**
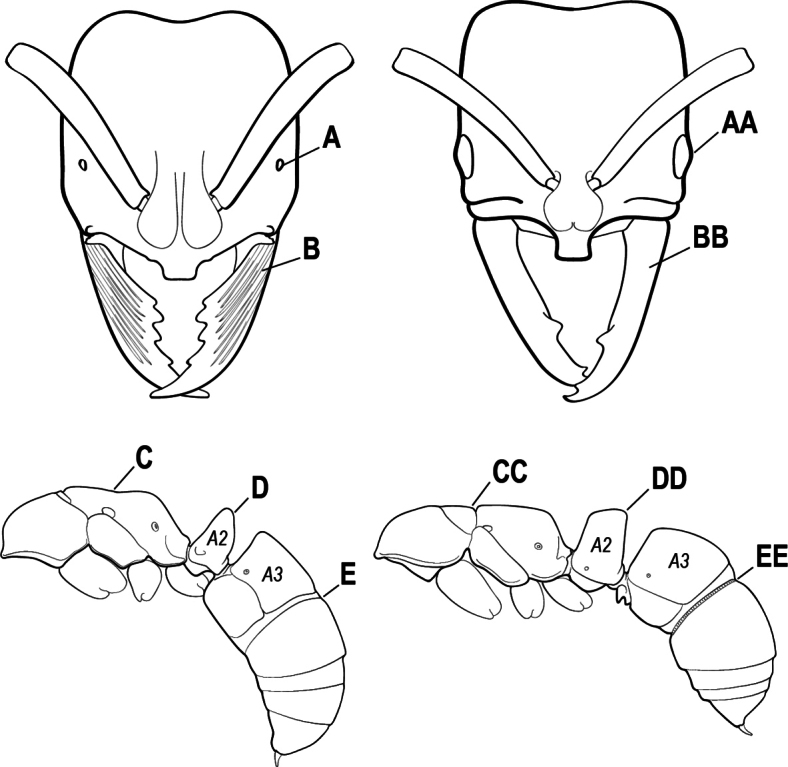		
22(20)	Head and body strongly striate (A); anterior clypeal margin with a row of specialized stout, dentiform setae (B); pronotum with a distinct tooth at each anterodorsal corner; dorsal margin of petiole emarginate-denticulate	*** Odontoponera ***
–	Head and body not strongly striate (AA), though light striations may be present on the sides of the mesosoma; anterior clypeal margin unarmed, without a row of specialized dentiform setae (BB); pronotum without a tooth at each anterodorsal corner; dorsal margin of petiole not emarginate-denticulate	**23**
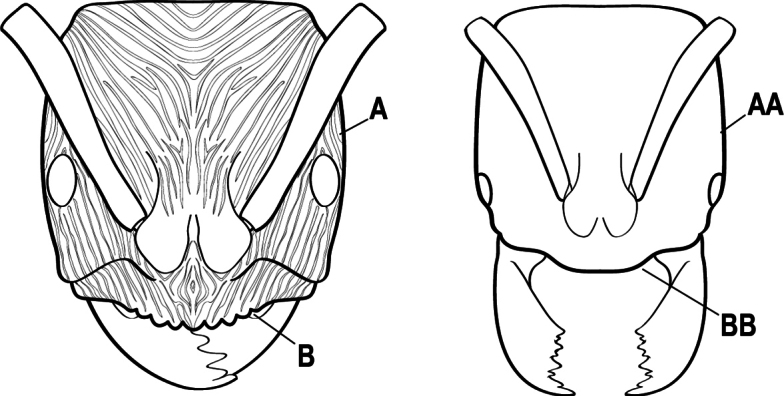		
23(22)	Prora present on anterior margin of first gastral sternite (A); basal portion of mandible without a dorsolateral pit, fovea, or sulcus (B)	**24**
–	Prora absent from anterior margin of first gastral sternite (AA); basal portion of mandible with a small circular or near-circular pit or fovea dorsolaterally (BB) or with a dorsolateral sulcus	**25**
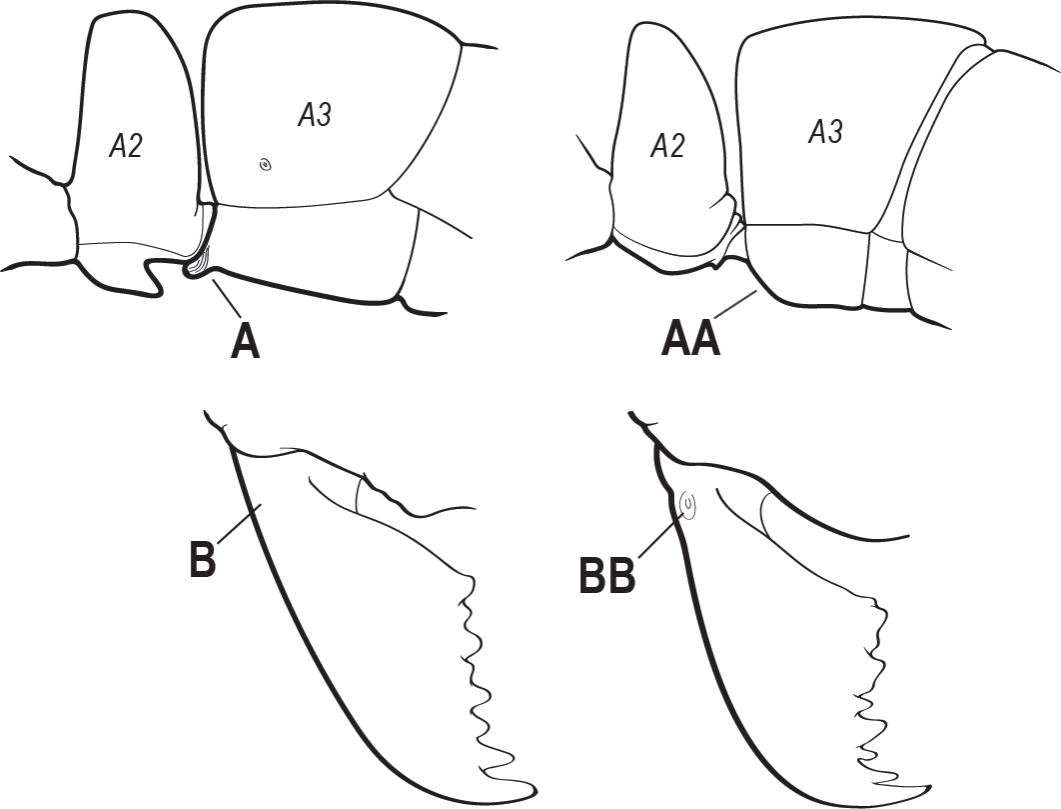		
24(23)	In profile view anterior clypeal margin rounded (A) and located against the mandibles; mandible elongate, their outer margins generally broadly and shallowly concave (B)	*** Mesoponera ***
–	In profile view, anterior clypeal margin angular (AA), with a conspicuous gap between anterior-most point and the mandibles; mandible shorter, their outer margins generally flat (BB) or with separate convex sections separated by a slight medial angle	***Austroponera* (part)**
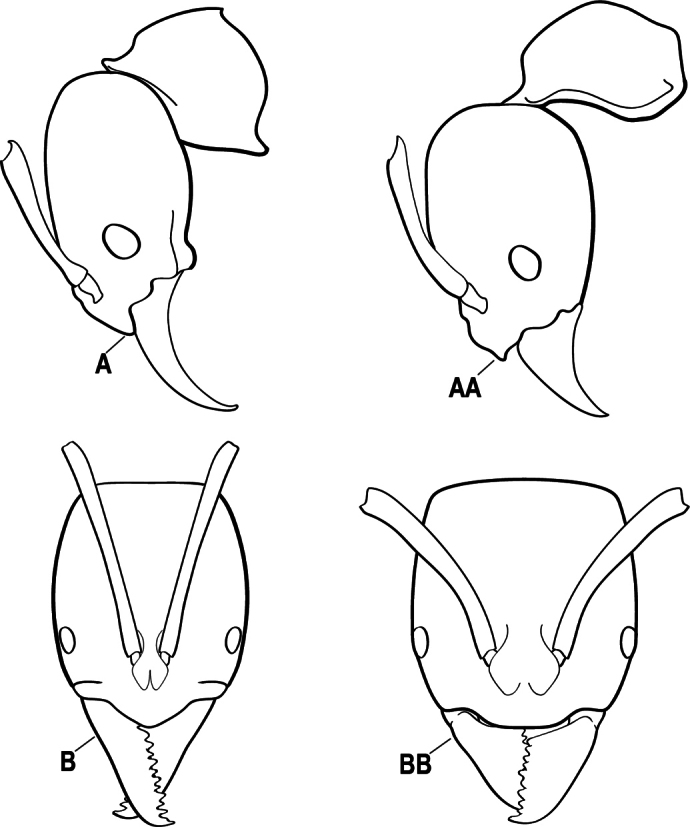		
25(23)	Basal portion of mandible with a small circular or near-circular pit or fovea dorsolaterally (A), sometimes with a short dorsolateral sulcus (AA), never with a sulcus that runs the entire length of mandible; in profile view, with mandible closed, anterior clypeal margin rounded (B)	*** Brachyponera ***
–	Basal portion of mandible without a dorsolateral pit but with a dorsolateral sulcus that runs the length of the mandible (AAA); in profile view, with mandible closed, anterior clypeal margin angular (BB)	***Myopias* (part)**
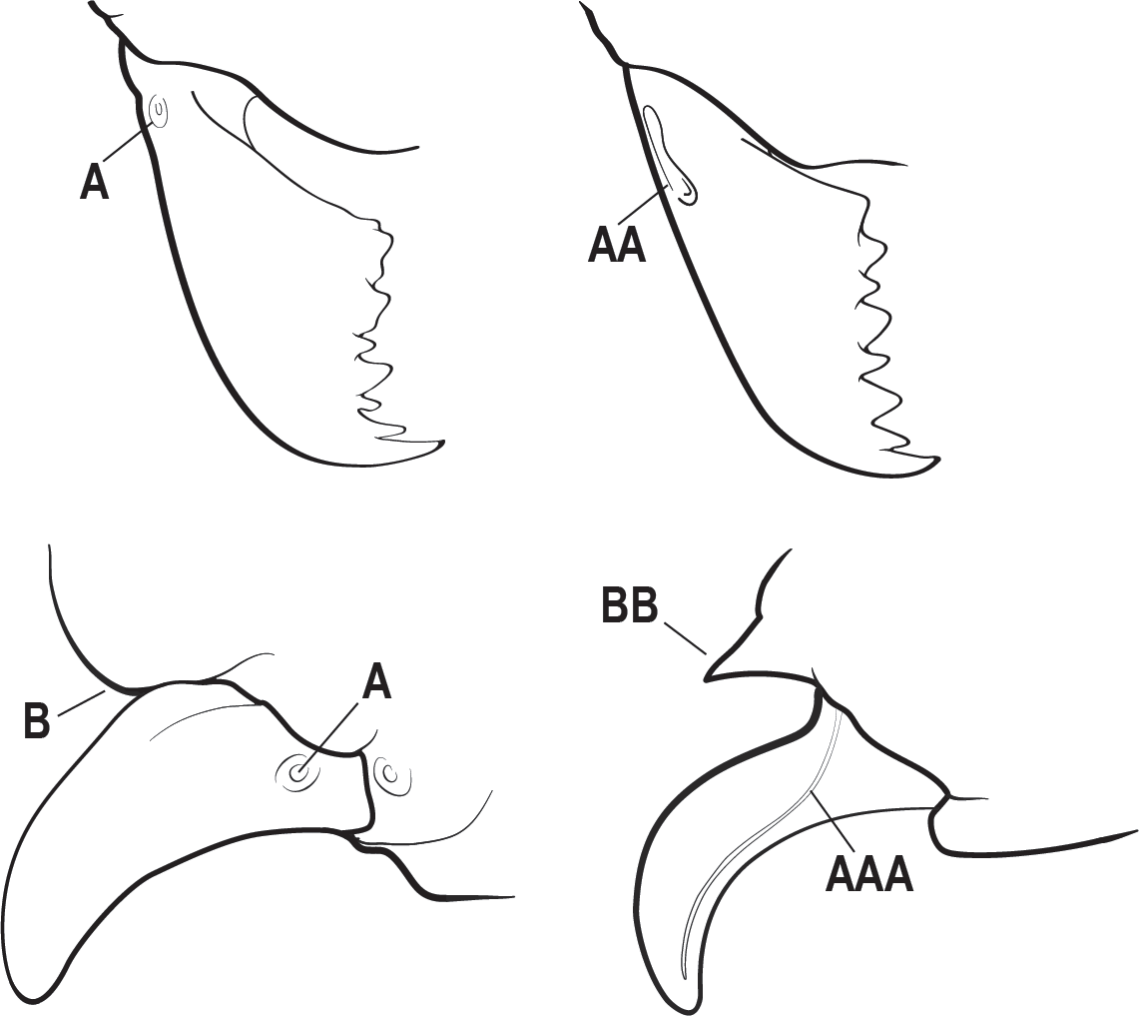		

#### Key to New World ponerine ant genera (workers) modified from Esteves and Fisher (2021)

**Table:** 

1	Mandible long and linear in full-face view, inserted at the middle of the anterior margin of the head; mandible bases closely approximate (A)	**2**
–	Mandible with variable shape, but always inserted at the anterolateral corners of the head; mandible bases conspicuously separated (AA)	**3**
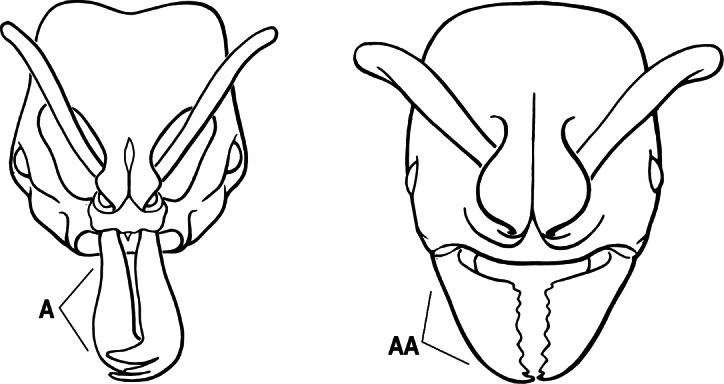		
2(1)	Nuchal carina (separating dorsal from posterior surfaces of head) converging in a V-shape at the midline (A), and also receiving a pair of prominent dark posterior apophyseal lines that converge to form the sharp median-dorsal groove of the vertex (B); dorsalmost tooth of apical mandibular series often truncated, rarely acute (C)	*** Odontomachus ***
–	Nuchal carina forming a broad, uninterrupted curve across the posterodorsal extremity of the head (AA); posterior surface without paired dark apophyseal lines; on vertex, the median groove absent or ill-defined and shallow (BB); dorsalmost tooth of apical mandibular series acute (CC)	*** Anochetus ***
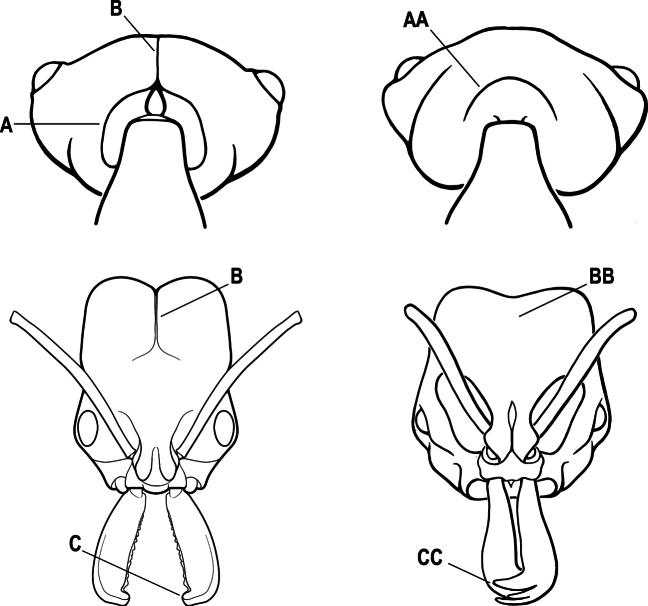		
3(1)	Clypeus broadly inserted between frontal lobes, which appear flattened in frontal view (A), the antennal sockets widely separated (B)	**4**
–	Clypeus narrowly inserted between frontal lobes (AA), antennal sockets closely approximated (BB)	**5**
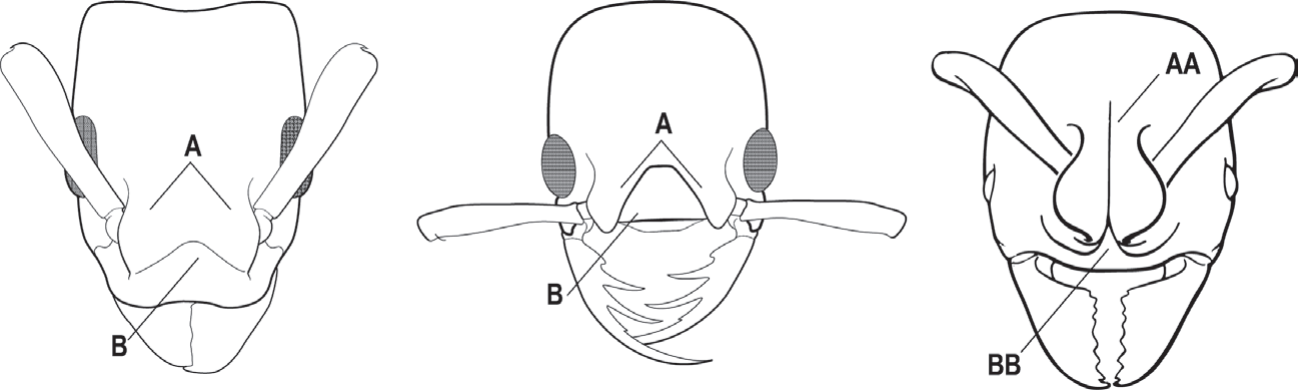		
4(3)	Mandible subtriangular, with numerous short teeth (A)	*** Platythyrea ***
–	Mandible with three long and attenuated teeth (AA)	*** Thaumatomyrmex ***
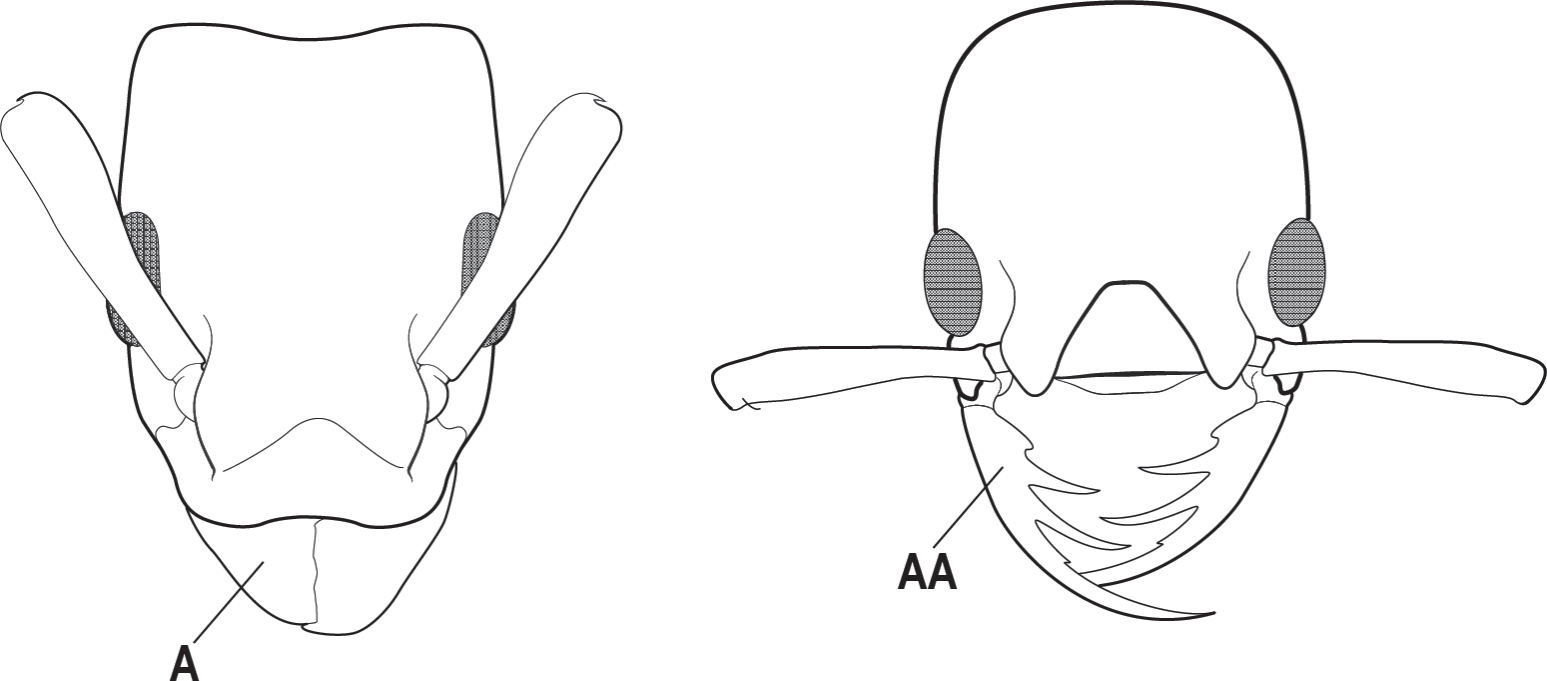		
5(3)	Ventral apex of metatibia, when viewed from in front with the metafemur at right-angles to the body, with a single large pectinate spur; without a second smaller spur in front of the pectinate main spur (A)	**6**
–	Ventral apex of metatibia, when viewed from in front with the metafemur at right-angles to the body, with 2 spurs, consisting of a large pectinate spur and a second smaller spur which is in front of the pectinate main spur (AA)	**9**
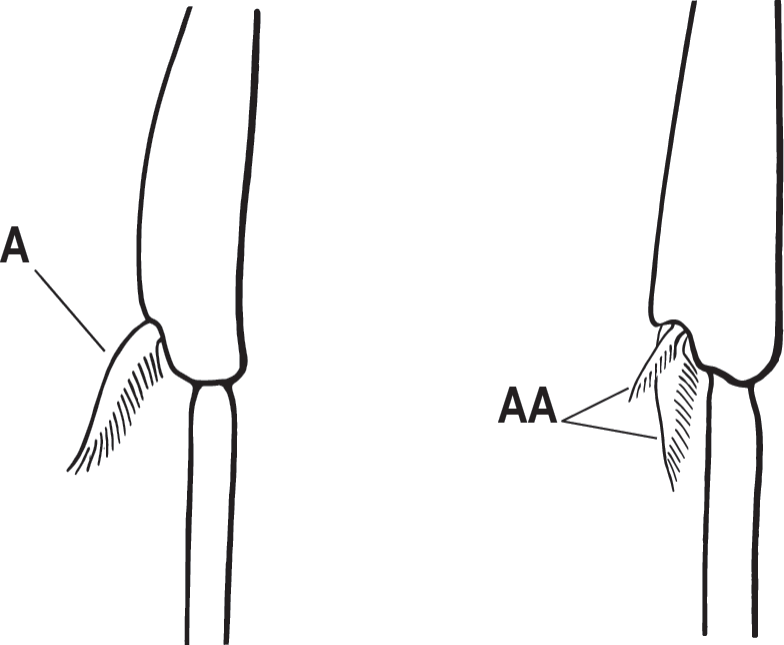		
6(5)	Dorsal (outer) surface of metabasitarsus with strong, thickly spiniform or peg-like traction setae (A), with or without additional simple setae; similar traction setae also always present on mesobasitarsus and mesotibia	*** Centromyrmex ***
–	Dorsal (outer) surface of metabasitarsus with simple setae only, without thickly spiniform or peg-like traction setae (AA); stout, spine-like setae may occur on either mesobasitarsus or mesotibia, together with simple setae, but if traction setae are present on either, then they are absent from metabasitarsus	**7**
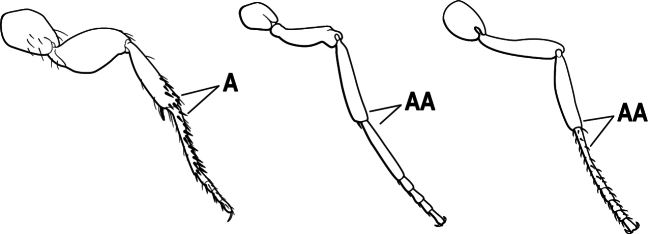		
7(6)	Medial portion of the clypeus projected anteriad, overhanging the anterior clypeal margin in full-face view (A); mandible subtriangular to falcate (B); arolia usually well-developed (C)	*** Simopelta ***
–	Medial portion of the clypeus not overhanging the anterior clypeal margin in full-face view; anterior clypeal margin slightly convex or with shallow concave notch medially (AA); mandible triangular (BB); arolia indistinct (CC)	**8**
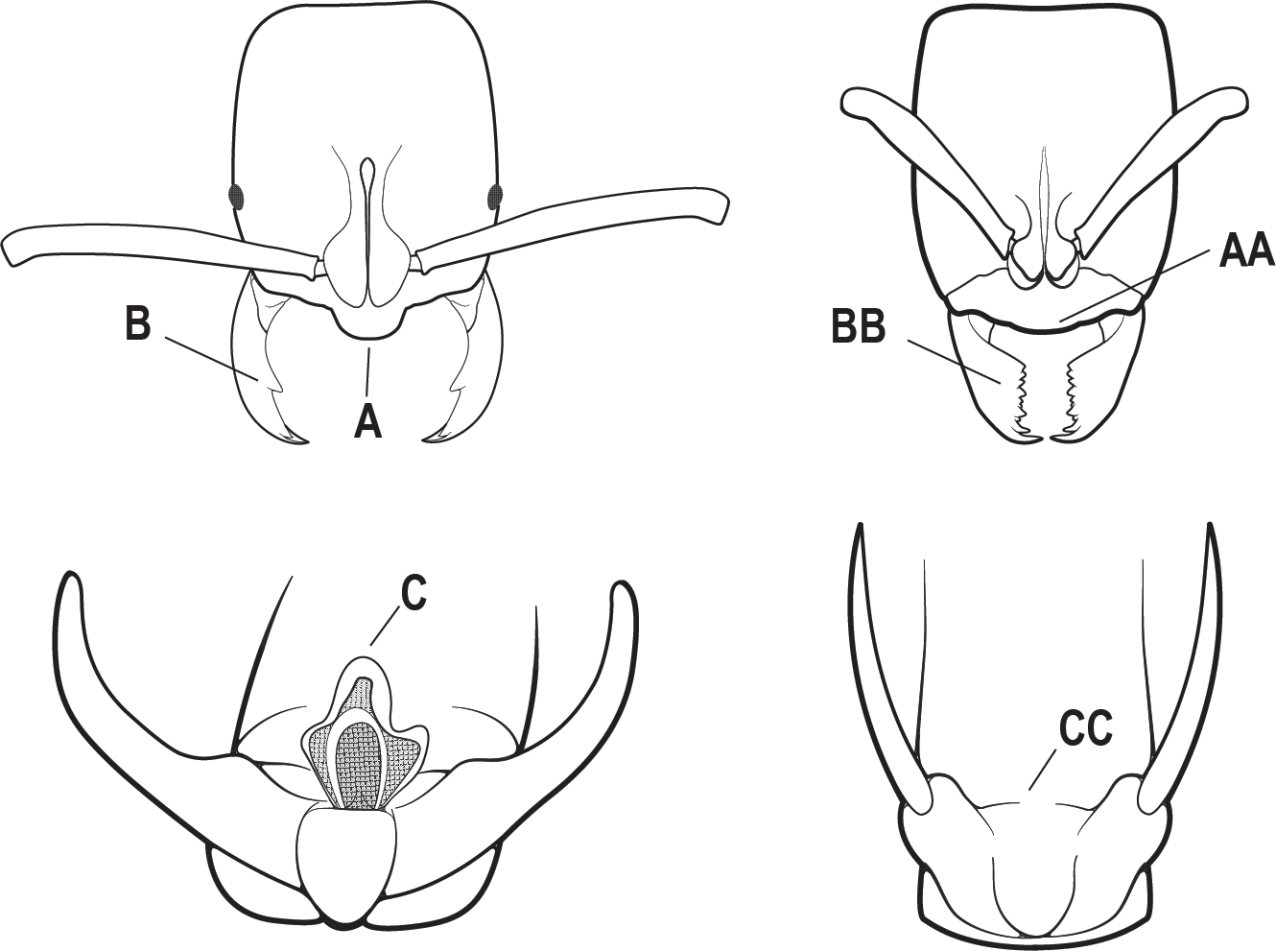		
8(7)	Subpetiolar process in profile with a pair of teeth posteroventrally (A) and with a fenestra or thin-spot anteriorly, which is translucent (B); maxillary palp with 2 segments	*** Ponera ***
–	Subpetiolar process in profile rounded (AA) to acutely angulate posteroventrally, but never with a pair of teeth; an anterior fenestra or thin-spot usually absent (BB), rarely present in some species; maxillary palp with 0 or 1 segment	*** Hypoponera ***
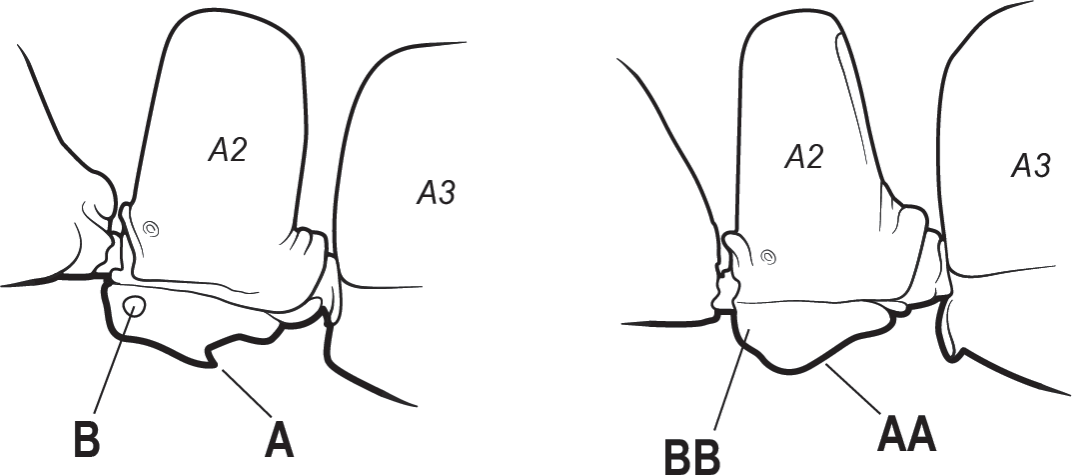		
9(5)	Pretarsal claw of metatarsus usually pectinate, extremely rarely with only 1 or 2 small teeth behind the apex (A); mandible with only 1–3 teeth (usually 2) (B), and frontal lobe not covering the entire antennal socket in full-face view (C)	*** Leptogenys ***
–	Pretarsal claw of metatarsus never pectinate, the claw simple (AA) or at most with teeth confined to the basal 1/3 or less (AAA); if preapical teeth present on claw, then mandible with 4 or more teeth (BB), and the frontal lobe fully concealing the antennal socket in full-face view (CC)	**10**
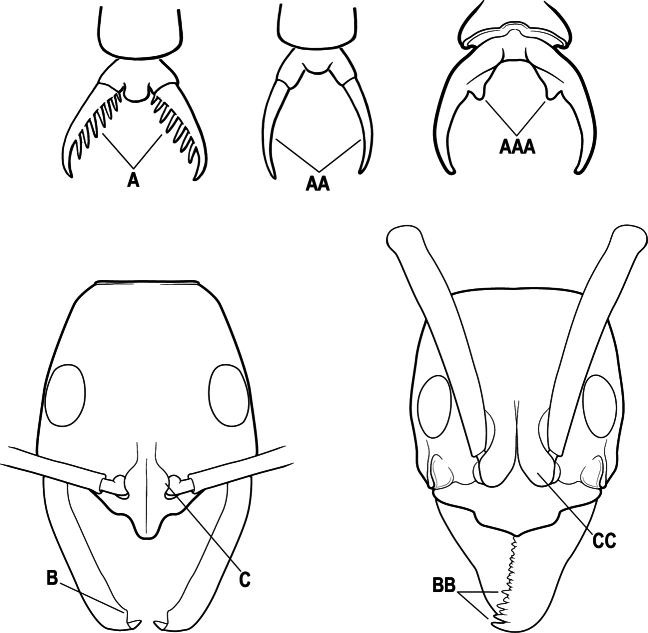		
10(9)	Subpetiolar process with sharply angulate posterior margin (A), so that in profile, the posterior portion of the subpetiolar process presents a long, acute projection strongly directed posteriad	**11**
–	Subpetiolar process rounded posteriorly or angulate; if angulate, then never forming a long, acute projection strongly directed posteriad (AA)	**12**
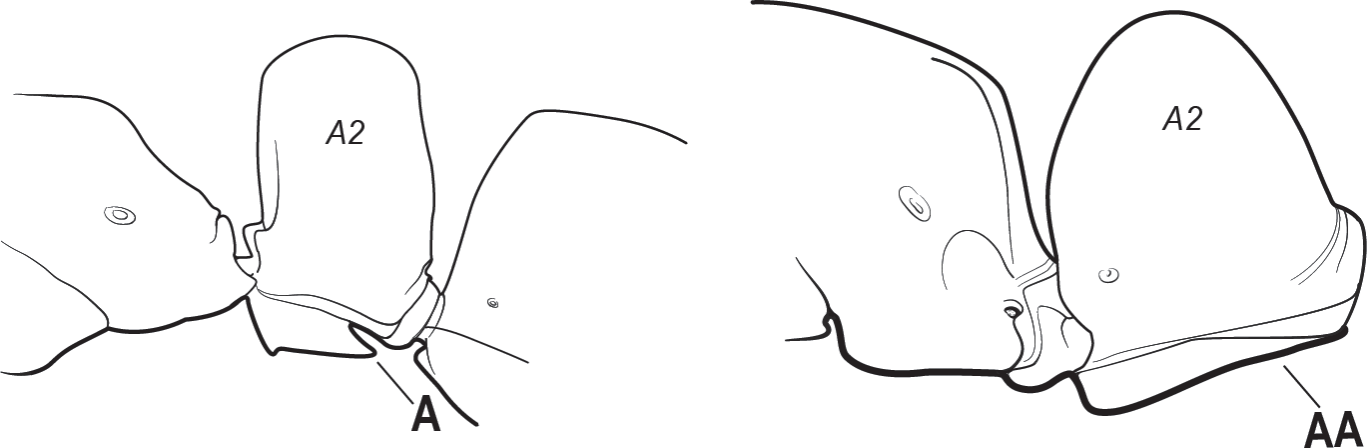		
11(10)	Basal portion of mandible with a dorsolateral large elongate pit (A); dorsal face of propodeum depressed below level of promesonotum in profile (B) (eastern North America)	***Brachyponera* (*B. chinensis*)**
–	Basal portion of mandible without a dorsolateral large elongate pit (AA); mesosomal dorsum forming an even curve, dorsal face of propodeum not depressed below promesonotum (BB) (Neotropics)	*** Rasopone ***
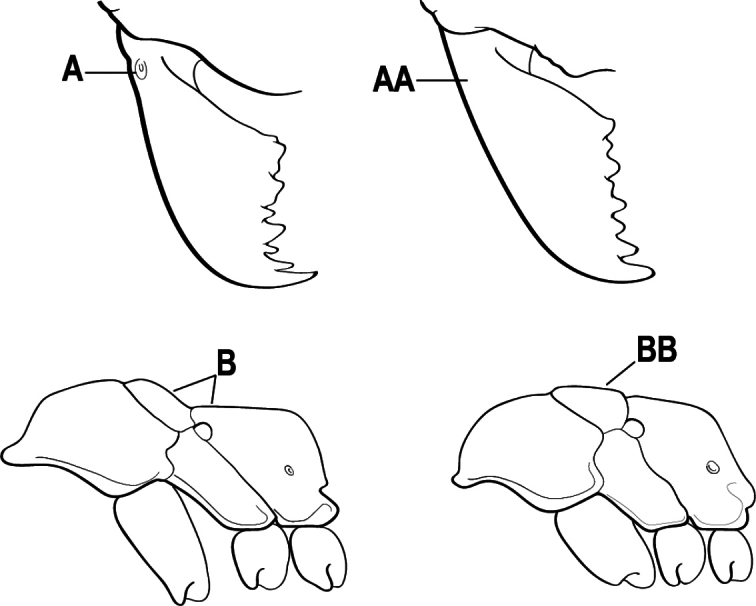		
12(10)	Propodeal spiracle slit-shaped (A); subpetiolar process flat to rounded posteriorly, not angulate (B); eye present and large, composed of many ommatidia, length along longest axis > 0.2 mm; large ants, HW > 1 mm	**13**
–	Differing in one or more of above characters; propodeal spiracle round or slit-shaped (AA); subpetiolar process variable, posterior margin flat, rounded, or angulate (BB); eye large to small or absent; often smaller ants	**17**
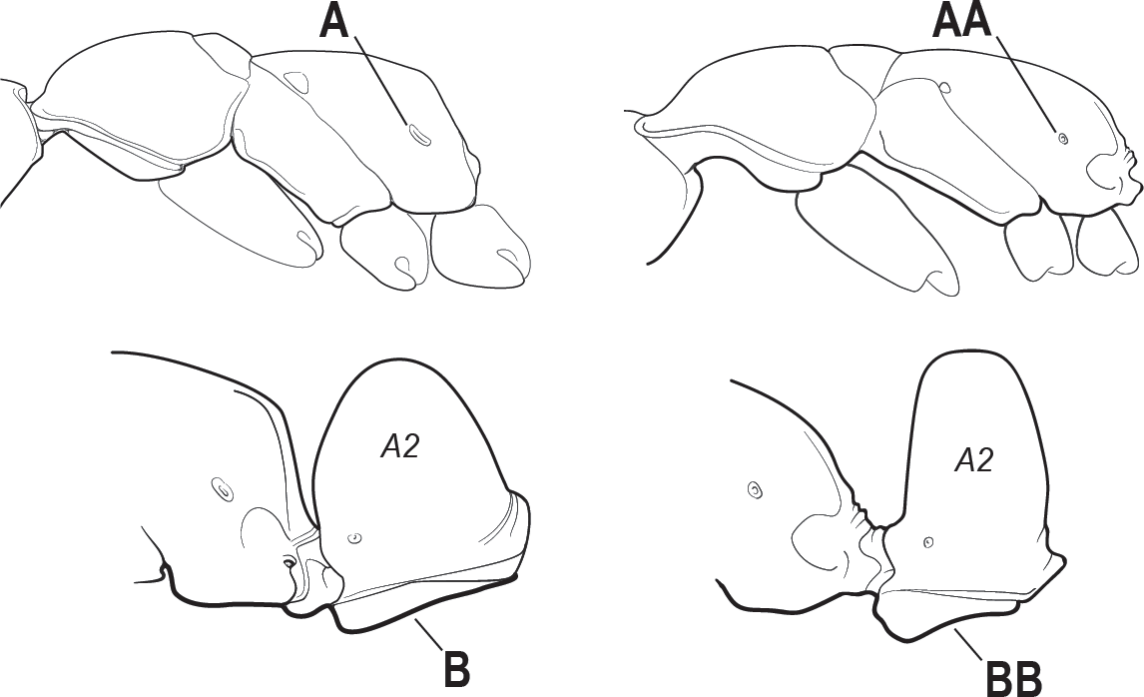		
13(12)	Body covered with coarse costate sculpture, starting on head and ending at petiole (A); mandible apex blunt (B); petiole subcuboidal; body covered with short, somewhat woolly pilosity (rare Amazonian species known from a single queen)	*** Igaponera ***
–	Differing in one or more of above characters; body sculpture usually striate, punctate, smooth, and shiny (AA), or combinations of these, but petiole never with costate sculpture; mandible apex acute (BB); petiole cuboidal to tapering and scale-like; pilosity variable	**14**
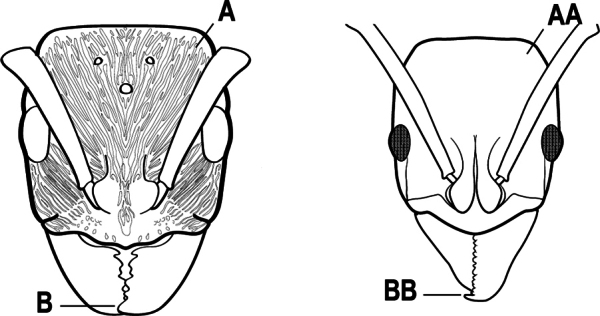		
14(13)	Massive ants (head width > 4.0 mm); anterior clypeal margin with a strong triangular tooth on each side projecting over the base of the mandible (A)	*** Dinoponera ***
–	Smaller ants (head width < 4.0 mm); anterior clypeal margin without teeth projecting over the base of the mandible (AA)	**15**
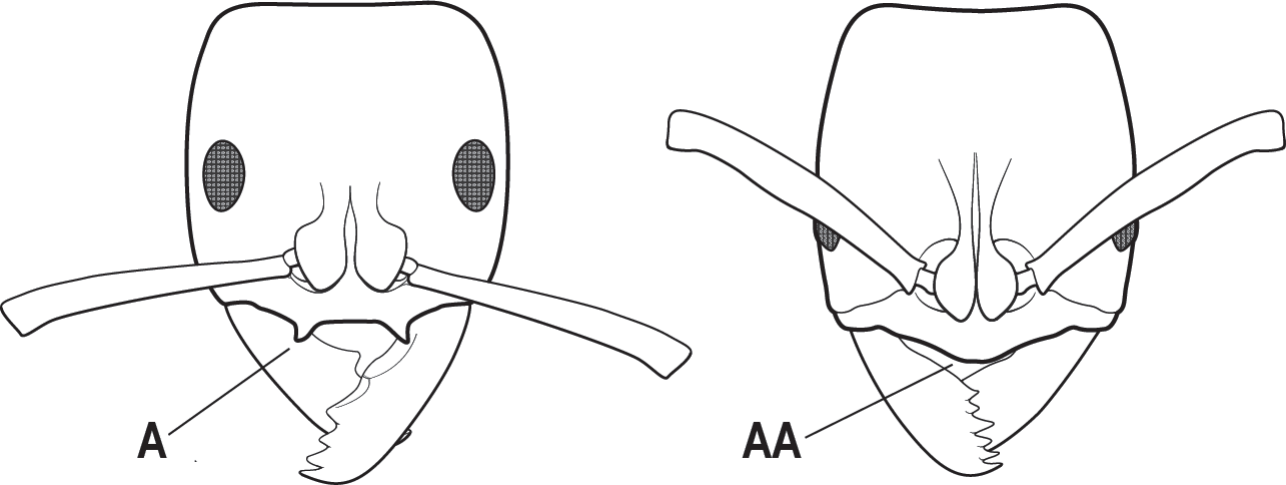		
15(14)	Tarsus with arolium (A); some ground foraging but mostly arboreal ants	***Neoponera* (part; most species)**
–	Tarsus lacks arolium (AA); large ground foraging ants	**16**
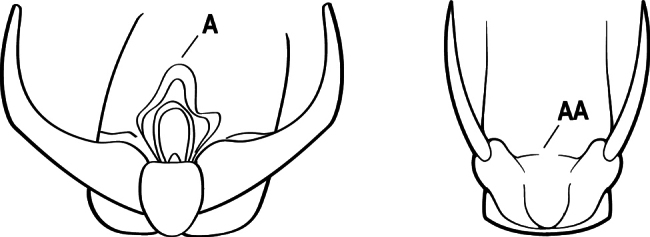		
16(15)	Petiole in profile with evenly rounded anterodorsal surface, meeting posterior face at abrupt right angle (A), posterior face flat (B); first gastral segment (A3) lacking standing setae (C); metapleural gland opening not bordered by cuticular rim or flange	**Unnamed Genus (*Neoponera bucki*)**
–	Petiole in profile cuboidal (AA) to tapering and scale-like; first gastral segment (A3) with standing setae (CC); metapleural gland opening bordered by cuticular rim or flange	*** Pachycondyla ***
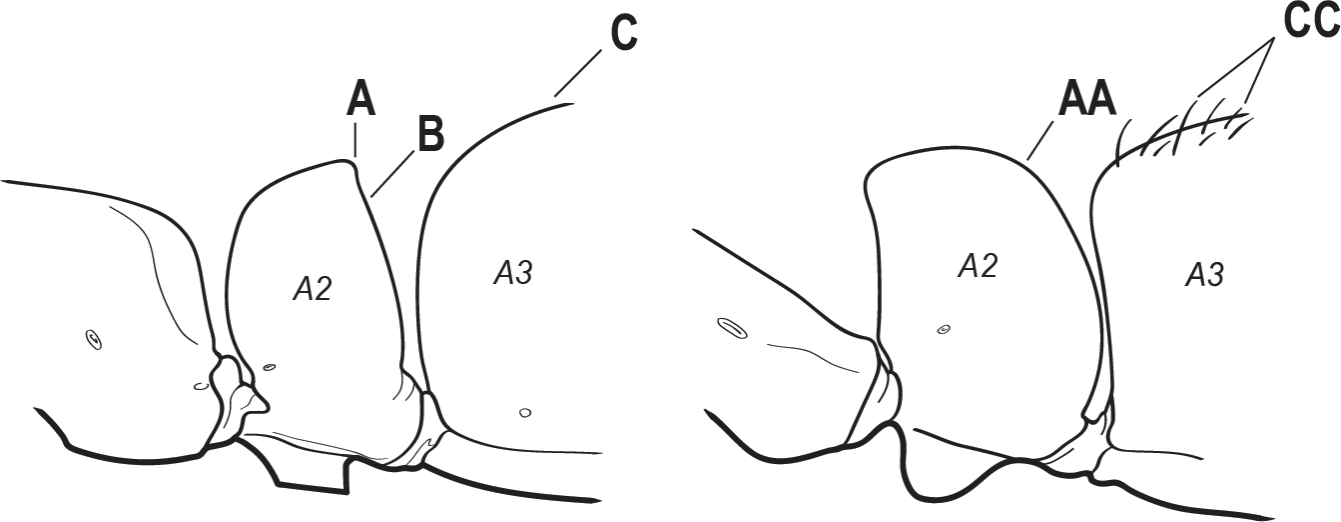		
17(12)	Dorsal surface of base of mandible smooth or with weak sulcus, never with rounded pit; mandible with 6 or 7 teeth/denticles (A); in profile, prora projected ventro-anteriorly as a long, acute prominence projecting ventro-anteriorly (B), in anterior view, prora transversely expanded; eye present but small, longest eye diameter < 0.15 mm	*** Pseudoponera ***
–	Differing in one or more of above characters; base of mandible with or without pit; mandible dentition variable (AA); prora absent or forming a short lobe, tooth, or lip, but never a long, flattened projection (BB); eye size variable	**18**
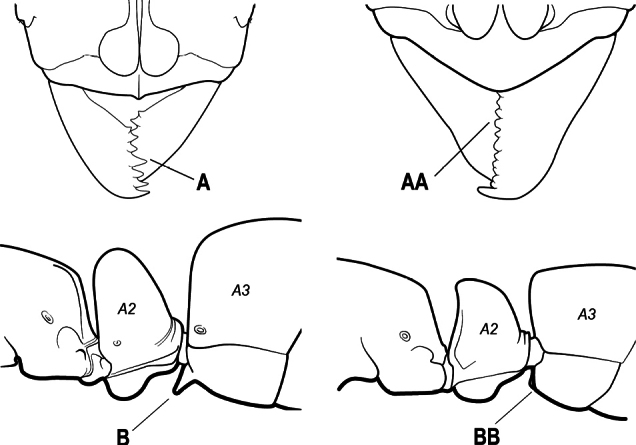		
18(17)	Mesotibia dorsally with abundant stout traction setae (A); eye absent or reduced to at most four partially fused ommatidia (intercaste females may have larger eye)	**19**
–	Mesotibia dorsally without abundant stout traction setae (a few stout setae sometimes present near tarsus but never extending along length of tibia) (AA); eye variable	**20**
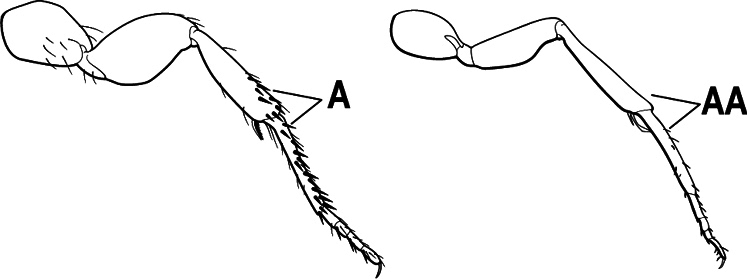		
19(18)	Basal portion of mandible with a dorsolateral pit (A); prora present as a low, blunt protuberance (B)	*** Cryptopone ***
–	Basal portion of mandible without a dorsolateral pit (AA); prora absent (BB)	*** Wadeura ***
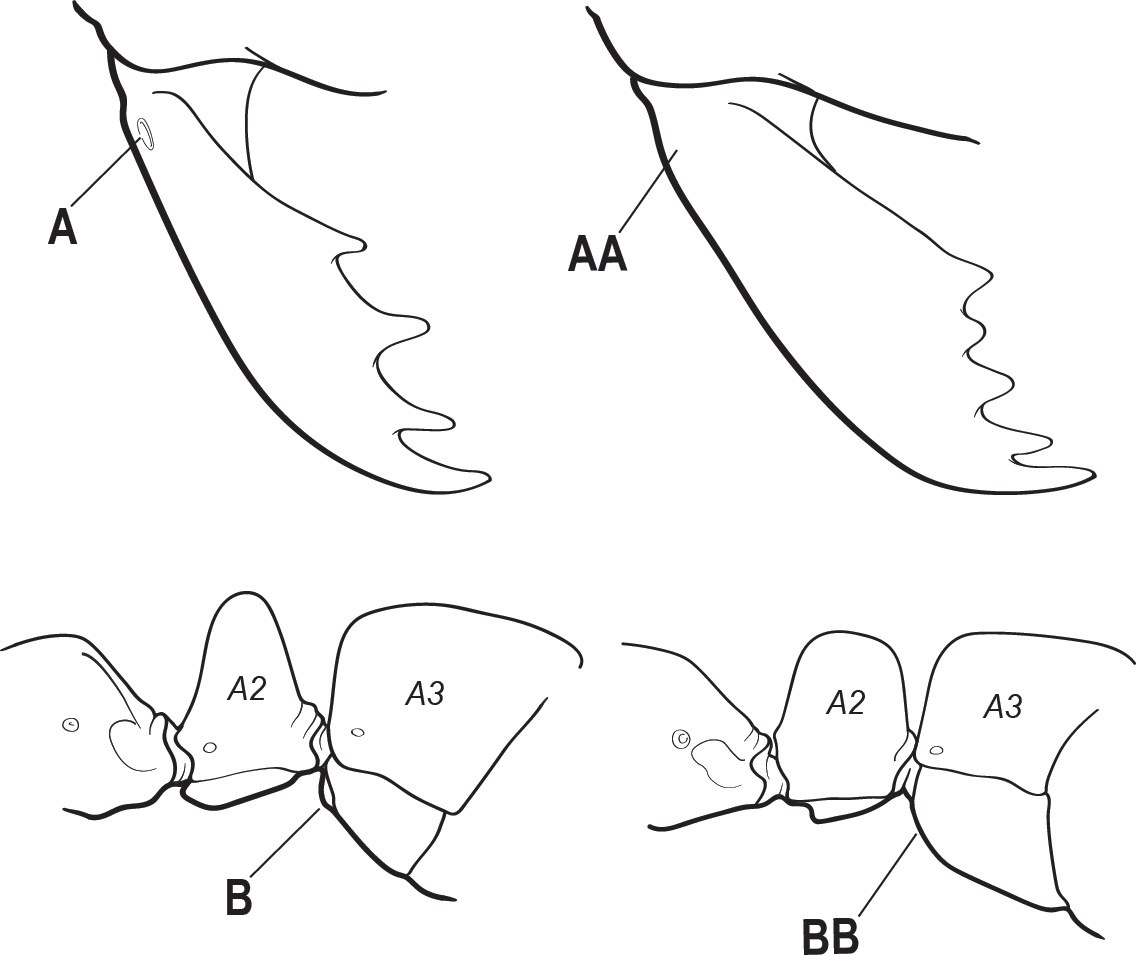		
20(18)	Eye relatively large, longest eye diameter > 0.1 × head width (A); mandible dentate, with > 6 subtriangular teeth (B)	**21**
–	Eye smaller, longest eye diameter < 0.1 × head width (AA); mandible edentate (BB) or with three long spiniform teeth (BBB)	**22**
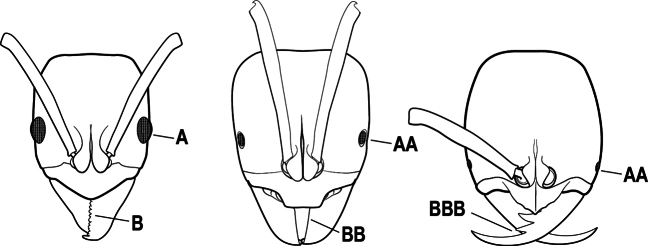		
21(20)	Head without a carina on each side that extends from the anterior margin of the eye to the clypeal margin (A)	*** Mayaponera ***
–	Head with a distinct carina on each side that extends from the eye to the clypeal margin (AA)	***Neoponera* (part, rare species with oval propodeal spiracle)**
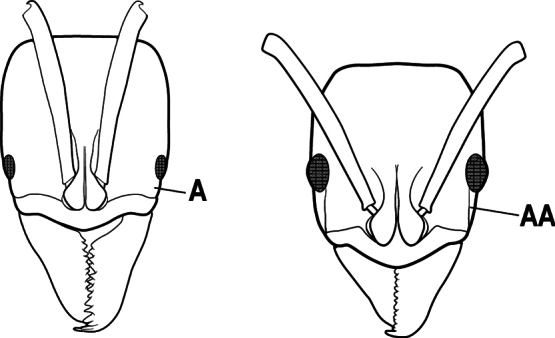		
22(20)	Mandible with three long spiniform teeth (A)	*** Belonopelta ***
–	Mandible edentate (AA)	*** Corrieopone ***
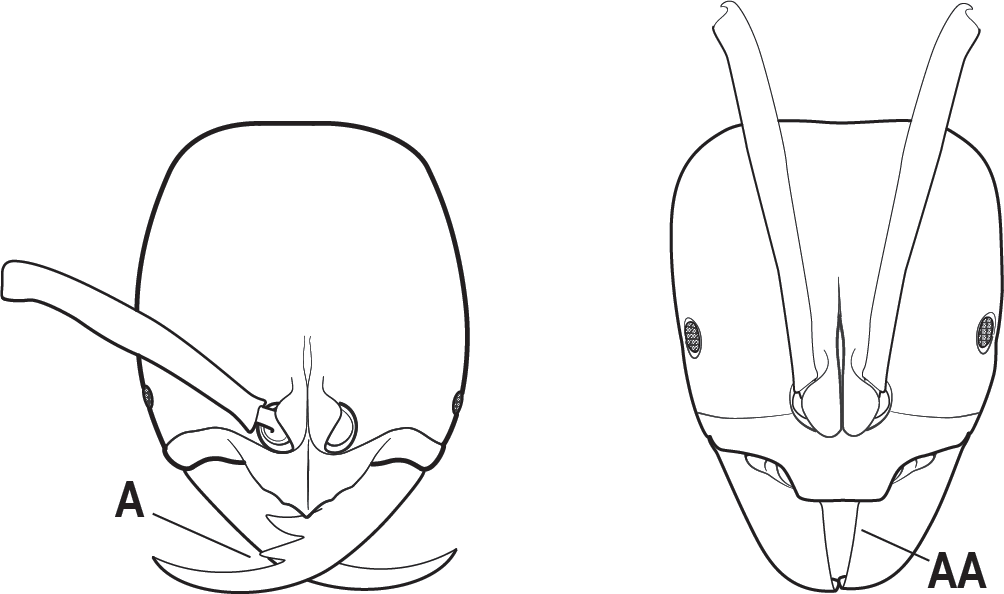		

### Taxonomic changes

#### Austroponera

Taxon classificationAnimalia

Schmidt & Shattuck

212199F6-E572-5DC5-8178-56F09937D3CE

##### Type species.

Our molecular phylogenetic results clearly place the former *Pseudoponera pachynoda* within *Austroponera*. Schmidt and Shattuck (2014) had separated *Pseudoponera* and *Austroponera* primarily based on the shape of the propodeal spiracle—slit-shaped in the former, rounded or oval in the latter—as well as the presence or absence of a stridulitrum on the pretergite of A4. Specifically, *Pseudoponera* was considered to lack a stridulitrum, while *Austroponera* was thought to possess one. The propodeal spiracle of *A. pachynoda* is more slit-shaped than any of the other three species of *Austroponera*, and this must be accepted as a variable character within the genus. Whether a stridulitrum is present in *A. pachynoda* has not been assessed in detail, but it is not evident in cursory examination. The stridulitrum is clearly visible in workers of *Austroponera castanea* and *A. rufonigra*. Other characters that differentiate *A. pachynoda* from *Pseudoponera* species are: (1) the small eye is positioned on the anterior surface of the head in full-face view rather than on the margin; (2) the mandible has more sharply differentiated basal and masticatory margins, rather than merging somewhat obliquely; and (3) the subpetiolar process has a strongly produced, acute posterior projection in profile, rather than forming a rounded lobe.

##### Diagnosis.

The genus *Austroponera*, as construed here, is quite variable morphologically even though well supported as a clade. All four species share the strong acute posterior projection of the subpetiolar process. Other traits show more divergence. The two New Zealand species, *A. castanea* and *A. castaneicolor*, are very similar, with relatively large eye, long scape, a propodeum depressed below an arched promesonotum, and a scale-like petiole. *Austroponera rufonigra* also has a scale-like petiole but shows a somewhat smaller eye, short scape, and a continuous, shallowly convex mesosomal dorsum. *Austroponera pachynoda* is the most divergent member of the genus, with an even smaller eye, short scape, a continuous mesosomal dorsum, a quadrate petiolar node, and a much deeper subpetiolar process than the other three.

##### Geographic distribution.

*Austroponera* is known from mainland Australia and New Zealand. *A. castanea* and *A. castaneicolor* are restricted to New Zealand; *A. rufonigra* and *A. pachynoda* occur on the Australian mainland.

##### Comments.

With *A. pachynoda* removed from *Pseudoponera*, the latter becomes a much more uniform genus, consisting of five very similar species. Its distribution also becomes more cohesive biogeographically, with four strictly Neotropical species and one, *P. stigma*, a pantropical tramp species most likely native to the Neotropics.

###### Species of *Austroponera*

*A. castanea* (Mayr, 1865): New Zealand.

= *A. castanea striata* (Stitz, 1911): New Zealand.

*A. castaneicolor* (Emery, 1893c): New Zealand.

*A. pachynoda* (Clark, 1930): Australia. comb. nov.

*A. rufonigra* (Clark, 1934): Australia.

= *A. clarki* (Wheeler, 1934): Australia.

#### Boltonopone gen. nov.

Taxon classificationAnimalia

3811759B-4DD6-5B78-9B3F-619B32C8DA52

https://zoobank.org/48FD1A50-10D5-40CC-8A63-5F3DBF763CB3

##### Type species.

*Ponera sulcata* Mayr, 1867b: 441; by present designation.

##### Phylogenetic relationships.

*Boltonopone* corresponds to the *Bothroponera sulcata* group as defined by Schmidt and Shattuck (2014) and further characterized by Joma and Mackay (2017). Its recognition as a distinct genus reflects both molecular phylogenetic evidence (see discussion under *Bothroponera*) and its cohesive set of morphological traits, which separate it from related *Bothroponera* species.

##### Diagnosis.

Members of *Boltonopone* possess the suite of characters attributed to the *Bothroponera sulcata* group in Schmidt and Shattuck (2014) and Joma and Mackay (2017). These include the presence of a metatibial gland; a subquadrate head slightly narrowed anteriorly with a concave posterior border; mandible shorter than head length bearing 6–9 teeth that alternate in size and are smooth or finely striate; and a broadly convex anterior clypeal border with a raised median area, sometimes showing longitudinal striae or a shallow groove. The frontal lobes are separated by a frontal furrow and are subquadrate anteriorly; the scape usually just surpasses the posterior lateral corners of the head. The eye is relatively small to moderate in size. The pronotal humerus is rounded, and the meso-metapleural suture is well developed. The mesonotum and propodeum are fused, with the propodeum posteriorly angulate or rounded and bearing an elongate propodeal spiracle. The petiole is large, with a visible spiracle and a developed sternopetiolar process. The metapleura are generally convex, though compressed in some species. Sculpture on the head and mesosoma is rough, sometimes punctate, and the body is variably colored (black, brown, or red), with moderately short to long erect golden or silver hairs, or in some cases nearly hairless. Males, where known, show a suborbiculate head with large eye, reduced notauli, and a small petiole relative to the propodeum. Full diagnostic discussion is provided under the treatment of *Bothroponera*.

##### Geographic distribution.

Species of *Boltonopone* are known from tropical Africa, India, Myanmar, and the Philippines.

##### Etymology.

The genus name *Boltonopone* is dedicated to Barry Bolton, whose extensive contributions to ant taxonomy, including his foundational global catalogues and revisions of the Ponerinae, have profoundly shaped modern myrmecology. The suffix -*pone* is derived from *Ponera*, the type genus of the subfamily, and is commonly used in Ponerinae generic names. The gender is feminine.

###### Species of *Boltonopone*

*B. ancilla* (Emery, 1899a): Congo. comb. nov.

*B. crassa* (Emery, 1877): Ethiopia. comb. nov.

*B. escherichi* (Forel, 1910b): Eritrea. comb. nov.

*B. glabripes* Emery, 1893b: Philippines. comb. nov.

*B. ilgii* (Forel, 1910b): Ethiopia. comb. nov.

= *B. crassior* Santschi, 1930: Kenya. comb. nov.

= *B. gamzea* (Özdikmen, 2010). comb. nov.

*B. kruegeri* (Forel, 1910a): South Africa. comb. nov.

= *B. asina* (Santschi, 1912): Kenya. comb. nov.

= *B. rhodesiana* (Forel, 1913a): Zimbabwe. comb. nov.

*B. notaula* Joma & Mackay, 2017: Tanzania. comb. nov.

*B. picardi* (Forel, 1901a): Angola. comb. nov.

*B. pilosuperficia* Joma & Mackay, 2017: Gabon. comb. nov.

*B. rubiginosa* (Emery, 1889): Myanmar. comb. nov.

*B. ryderae* Joma & Mackay, 2017: Guinea. comb. nov.

*B. silvestrii* (Santschi, 1914b): Ghana. comb. nov.

= *B. kenyensis* Santschi, 1937a: Kenya. comb. nov.

= *B. nimba* Bernard, 1953: Guinea. comb. nov.

*B. soror* (Emery, 1899a): Cameroon. comb. nov.

= *B. lamottei* Bernard, 1953: Guinea. comb. nov.

= *B. suturalis* (Forel, 1907b): Ethiopia. comb. nov.

*B. sulcata* (Mayr, 1867b): “Vaterland unbekannt.” comb. nov.

*B. sulcata fossulata* (Forel, 1900b): India. comb. nov.

*B. sulcata sulcatotesserinoda* (Forel, 1900b): India. comb. nov.

*B. tesseronoda* (Emery, 1877): India. comb. nov.

*B. williamsi* Wheeler, W.M. & Chapman, 1925: Philippines. comb. nov.

#### Bothroponera

Taxon classificationAnimalia

Mayr

02867173-26EA-5E24-AC96-92F05C9C327D

##### Type species.

Schmidt and Shattuck (2014) divided *Bothroponera* into two groups: *Bothroponera* (*sensu stricto*) and the *B. sulcata* group. Our phylogenetic results now corroborate that these two groups represent distinct and widely separated clades. We describe a new genus for the *B. sulcata* group herein. The newly circumscribed *Bothroponera* therefore corresponds to *Bothroponera* (*sensu stricto*) of Schmidt and Shattuck (2014), with its distinctive features confirmed by our molecular results.

##### Diagnosis.

Schmidt and Shattuck (2014) listed the diagnostic features of *Bothroponera* as follows: body without a long dense golden pilosity; mesopleuron usually not divided by a transverse groove; metanotal groove obsolete; propodeal dorsum without spines or teeth; propodeal spiracle slit-shaped; petiole nodiform (not semicircular in dorsal view) and without posterodorsal spines or teeth; tergite of abdominal segment III (A3) without strong longitudinal striations; gaster with a strong constriction between A3 and A4; and metatibia with two spurs. Within *Bothroponera* (s.s.), further distinguishing features include strong sculpturing, large cordate frontal lobe, a broad propodeal dorsum, and a U-shaped cuticular lip posterior to the metapleural gland orifice. These traits are lacking in the *B. sulcata* group. Additionally, *Bothroponera* (s.s.) has a stridulitrum on the pretergite of A4, whereas the *B. sulcata* group lacks it. The large cordate frontal lobe is a particularly distinctive and highly visible feature for identifying the genus in its revised circumscription.

##### Comments.

In their taxonomic discussion, Schmidt and Shattuck (2014) already noted that the combination of the two groups (*Bothroponera* (s.s.) and *B. sulcata* group) might be artificial and could represent separate lineages. Subsequent revisions by Joma and Mackay (2015, 2017, 2020) on the Afrotropical members of the *B. pumicosa*, *B. sulcata*, and *B. talpa* groups supported this view, applying largely the same characters as those of Schmidt and Shattuck to distinguish these lineages. Based on our phylogenomic results, we now formally separate the *B. sulcata* group into its own genus, described herein, and restrict *Bothroponera* to *Bothroponera* (s.s.), occurring only in the Afrotropical region, including Madagascar.

##### Geographic distribution.

*Bothroponera*, in its revised sense, is distributed in the Afrotropical region, including Madagascar.

###### Species of *Bothroponera*

*B. aspera* Arnold, 1962: South Africa.

*B. berthoudi* (Forel, 1901c): South Africa.

= *B. variolosa* Arnold, 1947: South Africa.

*B. cambouei* Forel, 1891: Madagascar.

= *B. jonesii* (Forel, 1891): Madagascar. syn. nov.

= *B. kipyatkovi* (Dubovikoff, 2013): Madagascar.

*B. cariosa* Emery, 1895b: Mozambique.

*B. cavernosa* (Roger, 1860): South Africa.

*B. comorensis* (André, 1887): Madagascar.

*B. cribrata* (Santschi, 1910): Congo.

*B. fugax* (Forel, 1907a): Tanzania.

*B. granosa* (Roger, 1860): South Africa.

*B. henryi* (Donisthorpe, 1942): India.

*B. laevissima* (Arnold, 1915): South Africa.

*B. masoala* (Rakotonirina & Fisher, 2013a): Madagascar.

*B. montivaga* Arnold, 1947: South Africa.

*B. pachyderma* (Emery, 1901): Cameroon.

= *B. attenata* (Santschi, 1920): Democratic Republic of the Congo.

= *B. funerea* Wheeler, 1922a: Democratic Republic of the Congo.

= *B. postsquamosa* (Santschi, 1920): Congo.

*B. perroti* Forel, 1891: Madagascar.

= *B. admista* Forel, 1892: Madagascar.

*B. planicornis* (Rakotonirina & Fisher, 2013a): Madagascar.

*B. pumicosa* (Roger, 1860): South Africa.

*B. rubescens* Santschi, 1937a: Democratic Republic of the Congo.

*B. sanguinea* (Santschi, 1920): Democratic Republic of the Congo.

*B. sculpturata* (Santschi, 1912): Zimbabwe.

= *B. mlanjiensis* Arnold, 1946: Malawi.

*B. strigulosa* Emery, 1895b: South Africa.

*B. talpa* André, 1890: Sierra Leone.

= *B. clavicornis* (Bernard, 1953): Guinea.

*B. talpa variolata* (Santschi, 1912): Congo.

*B. tavaratra* (Rakotonirina & Fisher, 2013a): Madagascar.

*B. umgodikulula* Joma & Mackay, 2015: South Africa.

*B. vazimba* (Rakotonirina & Fisher, 2013a): Madagascar.

*B. wasmannii* Forel, 1887: Madagascar.

*B. zumpti* Santschi, 1937b: Cameroon.

#### Bothroponera cambouei

Taxon classificationAnimalia

Forel, 1891

F9E37748-02A5-5550-A438-308B924F7219

Bothroponera cambouei Forel, 1891: 133. Lectotype worker: Madagascar: Imerina, Antananarivo (P. Camboué). [MHNG, CASENT0101770] (examined). Paralectotypes: 1 worker, 3 queens, same data as lectotype [MHNG].
Lobopelta jonesii Forel, 1891: 219. Holotype male: Madagascar: Forêt d’Andrangoloaka (F. Sikora) [MHNG, CASENT0101709] (examined). syn. nov.

##### Comments.

Forel (1891) described *Bothroponera cambouei* from workers and queens collected near Antananarivo, and *Lobopelta jonesii* from a single male from Forêt d’Andrangoloaka in the same region. The holotype male of *L. jonesii* is morphologically indistinguishable from males collected in colony series of *B. cambouei* from around Antananarivo (including body size, sculpture, coloration, and genital morphology), and no consistent characters separate them. We therefore treat *Lobopelta jonesii* Forel, 1891 (syn. nov.) as a junior synonym of *Bothroponera cambouei* Forel, 1891.

#### Brachyponera

Taxon classificationAnimalia

Emery

DBC26842-757E-52CD-94B5-963044F4F482

##### Type species.

An isolated male was described by Wheeler as a new genus and species, *Myrmapatetes filicornis*. This species was later transferred to *Anochetus* by Brown. Our molecular phylogenetic results place *Myrmapatetes filicornis* firmly within *Brachyponera*. This placement necessitates the synonymization of *Myrmapatetes* syn. nov. under *Brachyponera*.

#### Brachyponera filicornis

Taxon classificationAnimalia

(Wheeler, 1929a) comb. nov.

4A79C240-348A-5573-A03A-ABBA59487AE6

Myrmapatetes filicornis Wheeler, 1929a: 6, fig. 3. Holotype male: Indonesia: Tanimbar Is, Larat I., xii.1907 (F. Muir) [MCZC].
Anochetus filicornis : Brown, 1953: 2.

##### Note.

The sequenced male specimen, CASENT0842733 (UCDC collection), was identified as *Myrmapatetes filicornis* by Brendon Boudinot and Zach Griebenow based on close correspondence with the original description.

#### Cryptopone

Taxon classificationAnimalia

Emery

E10870BC-D0F9-5784-BFC6-EB7F87DB1D47

Iroponera Schmidt & Shattuck, 2014: 196. syn. nov.

##### Type species.

Schmidt and Shattuck (2014) described *Iroponera odax* from southern Australia as a highly distinctive ponerine, noting its unique combination of characters but leaving it unplaced due to limited data. A relationship to *Cryptopone* was already anticipated by W.L. Brown (unpubl. ms.; see discussion in Schmidt and Shattuck 2014) but this hypothesis was rejected by Schmidt and Shattuck because *Iroponera* lacked a mandibular pit and traction setae. Our molecular results now confirm Brown’s intuition, placing *Iroponera odax* well within *Cryptopone*, where it is sister to *Cryptopone rotundiceps*, a species from Australia and New Caledonia. It shows the general habitus of *Cryptopone* but apparently has lost the mandibular pit and traction setae.

##### Diagnosis.

Species of *Cryptopone* are generally small, cryptobiotic ponerines with triangular or falcate mandible, sometimes lacking a differentiated basal margin, and usually possessing a basal mandibular pit (although secondarily lost in *I. odax*). The mesotibiae typically bear stout traction setae, but these are lacking in *I. odax* and also in some small, undescribed species of Australian *Cryptopone*. The metatibia usually has two spurs, though in *I. odax* only a single spur is present. As noted, transitions from triangular to falcate mandible have evolved repeatedly in small cryptobiotic ponerines, as also seen in *Wadeura* and *Sritoponera* gen. nov.

##### Comments.

The synonymization of *Iroponera* within *Cryptopone* reflects its deep nesting within this lineage and demonstrates the morphological lability of mandible form and tibial structures in cryptobiotic ponerines. No apparent morphological synapomorphies currently unite *Iroponera*, the undescribed Australian species lacking mesotibial traction setae, and other *Cryptopone*; the synonymy is supported primarily by molecular phylogenetic evidence. This reinterpretation broadens the concept of *Cryptopone* to accommodate such secondary losses and morphological variability.

##### Geographic distribution.

*Cryptopone* is widely distributed in tropical and subtropical regions, with *C. odax* now recognized from southern Australia.

#### Cryptopone odax

Taxon classificationAnimalia

(Schmidt & Shattuck, 2014) comb. nov.

5CEDEEEF-FAA2-550F-858A-6CE5E0B6A3CE

Iroponera odax Schmidt & Shattuck, 2014: 198, fig. 46. Holotype worker: Australia: Tasmania, Pioneer State Forest, approx. 41°05'S, 147°56'E, 14.i.1992, sassafras gulley, in soil bank (B.B. Lowery & L. Gregson) [ANIC].

#### Euponera

Taxon classificationAnimalia

Forel

3F49369F-A7E9-5A65-B4CD-C5C9A63F5BC4

##### Type species.

Our newly restricted definition of *Euponera* (see discussion under *Fisheropone*) contains a single African species, *E. sjostedti*, together with a radiation of fourteen species from Madagascar.

##### Diagnosis.

Members of this clade share the presence of a basal mandibular pit, somewhat cordate frontal lobe, and a petiolar node that is relatively quadrate with a defined dorsal face.

##### Geographic distribution.

*Euponera* (sensu stricto) is distributed in tropical Africa and Madagascar.

###### Species of *Euponera*

*E. agnivo* (Rakotonirina & Fisher, 2013b): Madagascar.

*E. antsiraka* (Rakotonirina & Fisher, 2013b): Madagascar.

*E. daraina* (Rakotonirina & Fisher, 2013b): Madagascar.

*E. gorogota* (Rakotonirina & Fisher, 2013b): Madagascar.

*E. haratsingy* (Rakotonirina & Fisher, 2013b): Madagascar.

*E. ivolo* (Rakotonirina & Fisher, 2013b): Madagascar.

*E. maeva* (Rakotonirina & Fisher, 2013b): Madagascar.

*E. mialy* (Rakotonirina & Fisher, 2013b): Madagascar.

*E. nosy* (Rakotonirina & Fisher, 2013b): Madagascar.

*E. rovana* (Rakotonirina & Fisher, 2013b): Madagascar.

*E. sikorae* (Forel, 1891): Madagascar.

*E. sjostedti* (Mayr, 1896): Cameroon.

*E. tahary* (Rakotonirina & Fisher, 2013b): Madagascar.

*E. vohitravo* (Rakotonirina & Fisher, 2013b): Madagascar.

*E. zoro* (Rakotonirina & Fisher, 2013b): Madagascar.

#### Fisheropone

Taxon classificationAnimalia

Schmidt & Shattuck

3BCBBE16-09F7-53C9-95B4-227398A55E48

##### Type species.

Our molecular results revealed that several species previously assigned to *Euponera* form a paraphyletic assemblage with respect to *Fisheropone*. We considered either expanding the concept of *Fisheropone* to include them or describing multiple new genera. We chose the former, resulting in an expanded *Fisheropone* that includes these species. Consequently, *Fisheropone* cannot be readily diagnosed by morphology alone. The clade shows a progression from larger, darker species with a distinct compound eye to tiny yellow species that are eyeless and exhibit various degrees of character reduction, likely associated with transitions to a subterranean lifestyle. All species of *Fisheropone* as newly defined have a more scale-like petiole than species of true *Euponera*, which possess a relatively more quadrate petiole.

##### Diagnosis.

Because of the broadened circumscription, *Fisheropone* cannot be defined by a single set of morphological synapomorphies. However, a scale-like petiole is shared across the group, and no member has the quadrate petiole typical of *Euponera* (sensu stricto).

##### Morphological and phylogenetic structure.

The clade as redefined comprises several subgroupings:

The ***wroughtonii clade***, found in Africa, includes *Fisheropone wroughtonii* and *F. fossigera*, relatively large, dark ants with well-developed compound eye, a distinct mandibular pit, a slit-shaped propodeal spiracle, and two spurs on the metatibia.

The ***brunoi/sharpi clade***, occurring in Africa and Asia, consists of smaller, dark-colored ants with much smaller compound eye and a mandibular pit that is nearly or completely absent.

The ***ambigua>/>hartwigi clade***, restricted to Africa, comprises even smaller species with eye reduced to one or two ommatidia or completely absent, an oval or round propodeal spiracle, lighter coloration, and a single spur on the metatibia. *Fisheropone ambigua* lacks a mandibular pit, while *F. hartwigi* retains a distinct mandibular pit and has stout traction setae on the mesotibia—features that led to its original, but incorrect, placement in *Cryptopone*.

##### Geographic distribution.

*Fisheropone*, in its revised concept, is distributed throughout Africa and Asia, including Southeast Asia and the Indonesian region.

##### Taxonomic changes.

All of the Asian *Euponera* species (*grandis*, *malayana*, *pilosior*, *sakishimensis*, and *sharpi*) are very similar and are transferred here to *Fisheropone*. *Euponera pallidipennis* (Smith, 1860) based on a male from Indonesia is incertae sedis, but is moved to *Fisheropone* because true *Euponera* is unlikely to occur in Asia. Based on its description and illustration, *Euponera aenigmatica* from South Africa (small eye, dark coloration, scale-like petiole) is probably a member of the *brunoi/sharpi* clade and is here transferred to *Fisheropone*.

###### Species of *Fisheropone*

*F. aenigmatica* (Arnold, 1949): South Africa. comb. nov.

*F. ambigua* (Weber, 1942): South Sudan.

= *F. gulera* (Özdikmen, 2010).

*F. brunoi* (Forel, 1913c): Zimbabwe. comb. nov.

= *F. bayoni* (Menozzi, 1932): Uganda (Ssese Is).

= *F. dentis* (Weber, 1942): South Sudan.

= *F. lamottei* (Bernard, 1953): Guinea.

= *F. nigeriensis* (Santschi, 1914b): Nigeria.

= *F. nigeriensis katangana* (Santschi, 1933): Democratic Republic of the Congo.

*F. fossigera* (Mayr, 1901): South Africa. comb. nov.

*F. hartwigi* (Arnold, 1948): South Africa.

*F. grandis* (Donisthorpe, 1947): Vietnam. comb. nov.

*F. malayana* (Wheeler, 1929b): West Malaysia (Penang I.). comb. nov.

*F. pallidipennis* (Smith, 1860): Indonesia (Sulawesi). comb. nov.

*F. pilosior* (Wheeler, 1928): Japan. comb. nov.

= *F. chosonensis* (Teranishi, 1940): Japan.

*F. sakishimensis* (Terayama, 1999): Japan. comb. nov.

*F. sharpi* (Forel, 1901b): Singapore. comb. nov.

*F. wroughtonii* (Forel, 1901c): South Africa. comb. nov.

*F. wroughtonii crudelis* (Forel, 1901c): South Africa. comb. nov.

#### Makebapone gen. nov.

Taxon classificationAnimalia

6A97E70F-2695-5B25-BEED-12771A78428F

https://zoobank.org/E6E0B63B-E3BD-45B9-88B9-7D05FEFA6AB5

##### Type species.

*Ponera caffraria* Smith, 1858: 91; by present designation.

##### Diagnosis.

*Makebapone* shares the general characters of *Mesoponera* but differs in the following features: the masticatory margin of the mandible is short with low teeth; the clypeus is broadly rounded and somewhat inflated along the anterior margin; the propodeal spiracle is slit-shaped; the subpetiolar process forms an evenly convex lobe; and the prora is short and downturned.

##### Geographic distribution.

Species of *Makebapone* are found in tropical Africa.

##### Etymology.

The genus name *Makebapone* is derived from “Makeba”, honoring the renowned South African singer and activist Miriam Makeba (1932–2008), whose cultural influence and advocacy for African identity and justice resonate globally. The suffix -*pone* follows the convention established in many Ponerinae genera, derived from the type genus *Ponera*. The name thus links African heritage with the lineage of Ponerinae ants. The gender is feminine.

###### Species of *Makebapone*

*M. caffraria* (Smith, F. 1858): South Africa. comb. nov.

= *M. guineensis* (André, 1890): Sierra Leone. comb. nov.

*M. caffraria affinis* (Santschi, 1935): Congo. comb. nov.

*M. caffraria caffra* (Santschi, 1935): Guinea. comb. nov.

*M. ingesta* (Wheeler, 1922a): Democratic Republic of the Congo. comb. nov.

#### Mesoponera

Taxon classificationAnimalia

Emery

644D7A61-0157-588D-B45C-57D44A03DC50

##### Type species.

*Mesoponera* has been poorly defined since its original description by Emery (1900b). Schmidt and Shattuck (2014) provided a morphological diagnosis but acknowledged that the genus was artificial and would ultimately require partitioning. Molecular phylogenetic analysis now clearly reveals that *Mesoponera*, as previously constituted, comprises four distinct lineages. *Mesoponera* (sensu stricto) is restricted to Asia, Australasia, and the Seychelles, and is recovered as sister to a large clade containing *Myopias* and *Leptogenys*. A second clade, containing *M. ambigua*, represents an African lineage sister to *Brachyponera*; this clade has an available genus name, *Xiphopelta*, which is resurrected herein. A third clade containing *M. caffraria* is embedded within a mostly Afrotropical group, while a fourth clade containing *M. subiridescens* is part of a lineage with the African *Asphinctopone* and the Neotropical *Corrieopone*. The *M. caffraria* and *M. subiridescens* clades are described herein as new genera, *Makebapone* gen. nov. and *Subiridopone* gen. nov., respectively.

##### Diagnosis.

Schmidt and Shattuck (2014) characterized *Mesoponera* based on a relatively uniform habitus, which might be considered a ground-plan morphology for the *Odontomachus* genus group, with many characters appearing plesiomorphic. Esteves and Fisher (2021) described the features held in common across species previously assigned to *Mesoponera*, which include: the anterior portion of the head lacking a dorsolateral carina between the clypeal margin and the anterior margin of the eye; torular lobes closely approximated medially; anterior margin of the eye located at or in front of midlength of the head; mandible triangular, inserted at the anterolateral corners of the head, with masticatory margin bearing four or more teeth and lacking basolateral or dorsal pit (though an oblique dorsolateral sulcus may be present); the dorsoposterior area of the propodeum lacking spine-like or tooth-like projections; the mesotibia lacking stout spine-like setae on its dorsal face; the metatibia with two distinct spurs on its ventroapical face; the helcium positioned ventrad of midlength of the anterior face of abdominal segment IV; and the propodeum tectiform (roof-shaped), with lateral surfaces diverging and sloping ventrally from a noticeably narrow dorsum.

##### Comments.

Characters that unite the four phylogenetically recovered clades are probably all plesiomorphic, explaining why *Mesoponera* historically appeared cohesive morphologically despite its polyphyly. The present revision resolves this artificial grouping by restricting *Mesoponera* to its true core lineage and assigning the other lineages to appropriate genus-level names, either resurrected or newly described.

##### Geographic distribution.

*Mesoponera* (sensu stricto) is known primarily from Asia and Australasia, with *M. melanaria* (sensu lato) also occurring in the Seychelles.

###### Species of *Mesoponera*

*M. australis* (Forel, 1900a): Australia.

*M. javana* (Forel, 1905): Indonesia.

= *M. rubra minirubra* (Özdikmen, 2010).

*M. manni* (Viehmeyer, 1924): Solomon Islands.

= *M. robiginosa* (Donisthorpe, 1941): New Guinea (Indonesia).

*M. melanaria* (Emery, 1893b): Sri Lanka.

*M. melanaria macra* (Emery, 1894): Seychelles.

*M. papuana* (Viehmeyer, 1914): New Guinea (Papua New Guinea).

= *M. pulchella* (Donisthorpe, 1941): New Guinea (Indonesia).

= *M. viehmeyeri* (Donisthorpe, 1948b): New Guinea (Indonesia).

*M. rubra* (Smith, 1857): Singapore.

#### Parvaponera

Taxon classificationAnimalia

Schmidt & Shattuck

091C8A93-FCA7-5280-8506-6E2E5ED2D4D7

##### Type species.

Our molecular phylogeny revealed that *Parvaponera* sensu Schmidt and Shattuck (2014) is paraphyletic with respect to the Neotropical genus *Wadeura*. However, *Parvaponera* resolves into two morphologically distinct clades. *Parvaponera* (sensu stricto) contains *P. darwinii* (the type species) and *P. sheldoni*, and is sister to *Wadeura*. *Parvaponera* (s.s.) and *Wadeura* together are sister to a clade of African species that contains *P. suspecta* and related forms. We describe a new genus for the *P. suspecta* clade herein.

##### Diagnosis.

*Parvaponera* is now a more narrowly circumscribed genus. The diagnosis of Schmidt and Shattuck (2014) can be modified as follows: eye small (2–4 facets) or absent; mandible short, without a basal pit or groove; propodeal spiracle elongated or slit-like; ventral apex of the metatibia with one pectinate and one simple spur; subpetiolar process triangular with an anterior fenestra and lacking a pair of posterior teeth. *Sritoponera* gen. nov., the new genus containing *P. suspecta* and relatives, differs in having a rounded or oval propodeal spiracle and a subpetiolar process that is quadrate to trapezoidal with a posterior angle or teeth.

##### Morphology and variation.

Historically, *Parvaponera* has been known primarily from alate queens collected at lights. These queens have a broad geographic distribution throughout the Old World tropics. In spite of their wide range, very few workers are known, suggesting a cryptobiotic or subterranean lifestyle. There is evidence for two species that differ in size and in the shape of the anterior clypeal margin. *Parvaponera darwinii* is larger, with queen head width 0.86–1.13 mm and worker head width 0.83–0.85 mm; the anterior clypeal margin has at most a short tubercle. *Parvaponera sheldoni* is smaller, with queen head width 0.63–0.75 mm and worker head width 0.65–0.73 mm, and the anterior clypeal margin bears a distinct medial spine.

##### Geographic distribution.

*Parvaponera darwinii* is widespread in the Old World tropics and subtropics, extending northward to the United Arab Emirates. *Parvaponera sheldoni* occurs in Southeast Asia, Oceania, and Australia. The two species are sympatric in northern Australia.

##### Incertae sedis.

*Parvaponera cavimaculata*, from a single site in Hubei Province, China, remains incertae sedis in *Parvaponera*. The original description notes a head width of 0.75 mm with a transverse anterior clypeal margin lacking any tubercle or spine. It is also described as densely punctate and with dense, oblique rugae on the propodeum. The subpetiolar process is described as prominent and subtriangular. These sculptural features make it unlikely that this taxon belongs in *Parvaponera*.

###### Species of *Parvaponera*

*P. cavimaculata* (Wang & Zhao, 2009): China.

*P. darwinii* (Forel, 1893): Australia.

= *P. darwinii africana* (Forel, 1909): Democratic Republic of the Congo. syn. nov.

= *P. darwinii indica* (Emery, 1899b): Myanmar.

= *P. darwinii indica* (Forel, 1900b): India.

= *P. lamarki* (Santschi, 1913): Congo.

= *P. darwinii madecassa* (Emery, 1899b): Madagascar. syn. nov.

= *P. rufotestaceus* (Donisthorpe, 1943a): India.

*P. sheldoni* (Mann, 1919): Solomon Islands.

= *P. myropola* (Menozzi, 1925): Philippines. syn. nov.

#### Parvaponera darwinii

Taxon classificationAnimalia

(Forel)

65448589-4599-5643-B014-593D6CE7C57F

Belonopelta darwinii Forel, 1893: 460. Holotype queen: Australia: Port Darwin (J.J. Walker). [MHNG, CASENT0907290] (image examined).
Belonopelta darwini var. madecassa Emery, 1899b: 268. Syntype queens: Madagascar: [Baie d’Antongil], Maroantsetra (Mocquerys) [MHNG, CASENT0101691, [MSNG, CASENT0102012, CASENT0102013] (images examined). syn. nov.
Euponera (Pseudoponera) darwini var. africana Forel, 1909: 51. Holotype queen: Democratic Republic of the Congo: Luki (A. Jullien) [MHNG] (not examined). syn. nov.

##### Comments.

Emery described the subspecies madecassa from Madagascar, stating it was almost identical to the Australian type, with minor differences in size, color, and petiole shape. This form is now well-collected in Madagascar, with workers and queens. The UCE results place it in the single widespread species *darwinii*. Forel described the subspecies africana, differentiated by minor differences in petiole shape. We were not able to examine the type of *africana*, but assume Forel’s placement was correct and that this is a junior synonym of the widespread *darwinii*.

#### Parvaponera sheldoni

Taxon classificationAnimalia

(Mann)

079A9726-7136-5309-91C6-49046D16D351

Euponera (Trachymesopus) sheldoni Mann, 1919: 292. Holotype worker: Solomon Is: San Cristoval I. (= Makira I.), Wainoni Bay (W.M. Mann). [USNM, USNMENT00532034] (image examined).
Euponera (Trachymesopus) myropola Menozzi, 1925: 444. Holotype queen: Philippines: Luzon I., Laguna, Los Baños (C.F. Baker) [MCZC] (MCZ-ENT00028879, image examined). syn. nov.

##### Comments.

The head width of Menozzi’s *myropola*, based on the MCZ images, is 0.74 mm, within the size range of *sheldoni*. It is difficult to discern from the image, but it may have the clypeal spine of *sheldoni*.

#### Sritoponera gen. nov.

Taxon classificationAnimalia

0CBE4C93-3632-5BA9-B8EF-0FCB5F4869EC

https://zoobank.org/40C6D259-6939-40DE-A15E-31BD7A7C98DF

##### Type species.

Euponera (Trachymesopus) suspecta Santschi, 1914a: 51; by present designation.

##### Phylogenetic relationships.

Schmidt and Shattuck (2014) left *P. suspecta* incertae sedis in *Euponera* without examination. Fisher and Bolton (2016) recognized it within the broader sense of *Parvaponera*. Here we recognize it as a separate lineage of cryptobiotic ponerines, related to *Parvaponera* and *Wadeura* (see further discussion under *Parvaponera*).

##### Diagnosis.

*Sritoponera* species have eyes that are small (2–4 facets) or absent; mandible short, without a basal pit or groove; the propodeal spiracle rounded or oval; ventral apex of the metatibia bearing one pectinate and one simple spur; and the subpetiolar process quadrate to trapezoidal in shape, with a posterior angle or teeth, and the anterior fenestra present or absent.

##### Geographic distribution.

Species of *Sritoponera* are known from tropical Africa.

##### Comments.

*Sritoponera suspecta* is currently the only described species in the genus, although there are additional undescribed African morphospecies that likely belong here.

##### Etymology.

The genus name *Sritoponera* is derived from the Ancient Egyptian word *srit*, meaning “small,” in reference to the minute body size and reduced eye of species in this lineage. It is combined with the suffix -*pone*, derived from *Ponera*, the type genus of the subfamily and a common element in Ponerinae generic names. The gender is feminine.

###### Species of *Sritoponera*

*S. suspecta* (Santschi, 1914a): Tanzania. comb. nov.

#### Subiridopone gen. nov.

Taxon classificationAnimalia

FFDA1855-3515-5A5E-83C6-B26940EDE66C

https://zoobank.org/D30E0BF0-3D20-4ACA-96F4-F3FC2492F194

##### Type species.

Euponera (Mesoponera) subiridescens Wheeler, 1922a: 83; by present designation.

##### Phylogenetic relationships.

Our molecular analyses place *Subiridopone* within a clade that includes *Asphinctopone* (Afrotropical) and *Corrieopone* (Neotropical), separate from the true *Mesoponera* lineage. This position supports its recognition as a distinct genus.

##### Diagnosis.

*Subiridopone* shares many characters with *Mesoponera*, but differs in the following combination of traits: the masticatory margin of the mandible is long, with a proximal portion that is edentate; the clypeus is transverse and somewhat truncate; the katepisternum and anepisternum are separated by a sulcus; the propodeal spiracle is slit-shaped; the subpetiolar process has a bluntly angulate posterior margin; and the prora is a pronounced, thin flange that is broadly shovel-shaped. In comparison, *Mesoponera* (s.s.) has a pronounced prora that narrows anteriorly to a blunt or acute point, *Xiphopelta* has a broadly shovel-shaped but thicker prora, and *Makebapone* has a short, ventrally directed prora that does not strongly project anteriorly.

##### Geographic distribution.

*Subiridopone* is currently monotypic, containing only the type species, and is known from the Afrotropical region.

##### Etymology.

The genus name *Subiridopone* is derived from the epithet of its type species, *subiridescens* Wheeler, 1922. The Latin root *irid*- (“rainbow, iridescence”) refers to a sheen or subtle coloration, while the prefix *sub*- indicates “somewhat” or “slightly.” The name thus preserves the descriptive origin of the species while providing a euphonious and distinctive generic name. The suffix -*pone* is derived from *Ponera*, the type genus of the subfamily and a common element in Ponerinae generic names. The gender is feminine.

###### Species of *Subiridopone*

*S. subiridescens* (Wheeler, 1922a): Democratic Republic of the Congo. comb. nov.

#### Xiphopelta

Taxon classificationAnimalia

Forel stat. rev.

E1058C1E-0970-5DA4-8A07-77C329BEC824

Xiphopelta Forel, 1913a: 108 [as subgenus of Ponera Latreille, 1804]. Type species: Ponera arnoldi Forel, 1913a (now: junior synonym of Xiphopelta elisae rotundata (Emery, 1895b)), by monotypy.
Xiphopelta as subgenus of Euponera: Forel 1917: 237.
Xiphopelta as junior synonym of Pachycondyla: Brown 1973: 185.
Xiphopelta as junior synonym of Mesoponera: Wheeler 1922b: 775; Schmidt and Shattuck 2014: 107.

##### Type species.

Our molecular analyses support the resurrection of *Xiphopelta* as a distinct genus, separate from *Mesoponera* (sensu stricto). Its lineage is clearly recovered as a monophyletic African radiation, sister to *Brachyponera*.

##### Diagnosis.

Species of *Xiphopelta* can be distinguished from *Mesoponera* by the following combination of features: (1) the mandible has a relatively longer masticatory margin, which is dentate; (2) the clypeus bears a small anteromedian clypeal denticle; (3) the propodeal spiracle is positioned relatively more posteroventrally; (4) the subpetiolar process has a strongly projecting posterior angle; and (5) the prora is anteriorly directed and shelf-like.

##### Geographic distribution.

*Xiphopelta* is known from tropical Africa.

###### Species of *Xiphopelta*

*X. ambigua* (André, 1890): Sierra Leone. comb. nov.

*X. elisae* (Forel, 1891): Madagascar. comb. rev.

*X. elisae divaricata* (Emery, 1915): Eritrea. comb. nov.

*X. elisae redbankensis* (Forel, 1913a): Zimbabwe. comb. rev.

*X. elisae rotundata* (Emery, 1895b): South Africa. comb. rev.

= *X. arnoldi* (Forel, 1913a): Zimbabwe. comb. rev.

*X. flavopilosa* (Weber, 1942): South Sudan. comb. nov.

*X. nimba* (Bernard, 1953): Guinea. comb. nov.

= *X. neonimba* (Özdikmen, 2010). comb. nov.

*X. novemdentata* (Bernard, 1953): Guinea. comb. rev.

*X. picea* (Bernard, 1953): Guinea. comb. rev.

*X. scolopax* (Emery, 1899a): Cameroon. comb. nov.

*X. senegalensis* (Santschi, 1914b): Senegal. comb. rev.

*X. testacea* (Bernard, 1953): Guinea. comb. nov.

*X. villiersi* (Bernard, 1953): Guinea. comb. nov.

*X. weberi* (Bernard, 1953): Guinea. comb. nov.

## Supplementary Material

XML Treatment for Austroponera

XML Treatment for Boltonopone

XML Treatment for Bothroponera

XML Treatment for Bothroponera cambouei

XML Treatment for Brachyponera

XML Treatment for Brachyponera filicornis

XML Treatment for Cryptopone

XML Treatment for Cryptopone odax

XML Treatment for Euponera

XML Treatment for Fisheropone

XML Treatment for Makebapone

XML Treatment for Mesoponera

XML Treatment for Parvaponera

XML Treatment for Parvaponera darwinii

XML Treatment for Parvaponera sheldoni

XML Treatment for Sritoponera

XML Treatment for Subiridopone

XML Treatment for Xiphopelta

## Additional information

### Use of AI

No use of AI was reported.

### Author ORCIDs

Brian L. Fisher https://orcid.org/0000-0002-4653-3270

Michael G. Branstetter https://orcid.org/0000-0002-3734-6166

Bonnie B. Blaimer https://orcid.org/0000-0002-8961-9998

Marek L. Borowiec https://orcid.org/0000-0001-7510-4064

Gabriela P. Camacho https://orcid.org/0000-0002-8792-7719

Maël Doré https://orcid.org/0000-0003-3242-9242

Philip S. Ward https://orcid.org/0000-0002-9019-0383

John T. Longino https://orcid.org/0000-0001-5465-0293

### Data availability

Raw reads for newly sequenced samples are available in the NCBI Sequence Read Archive under BioProject# PRJNA1291805. Assembly contig files, phylogenetic matrices, tree files, and analysis log files are available in the Zenodo Digital Repository: https://zenodo.org/records/17885727. COI DNA barcode sequences, serving as molecular vouchers, are available in GenBank under accessions PX697285–PX697579.

## Supplementary materials

Supplementary material 1 Taxonomic changes in Ponerinae proposed in this study Data type xlsx Explanation note Taxonomic changes in Ponerinae proposed in this study. This table presents the nomenclatural acts resulting from our phylogenomic revision of the ant subfamily Ponerinae (Hymenoptera: Formicidae). Changes include new genera (gen. nov.), revalidated taxa (stat. rev.), new synonymies (syn. nov.), and new combinations (comb. nov.). For genera that are split or otherwise redefined, the table also lists taxa that remain unchanged (“no change”) to explicitly confirm their retention in the revised genus concept. Each row provides: the genus combination prior to this study (Prior placement), the original taxon name (Protonym / original combination), author and year, status, revised genus placement based on this work, the nomenclatural act, bioregion, and country of the type specimen. This table complements the main text and ensures transparency of all taxonomic decisions, facilitating integration into databases such as AntCat. https://binary.pensoft.net/file/1496661This dataset is made available under the Open Database License (http://opendatacommons.org/licenses/odbl/1.0/). The Open Database License (ODbL) is a license agreement intended to allow users to freely share, modify, and use this Dataset while maintaining this same freedom for others, provided that the original source and author(s) are credited.Brian L. Fisher, Michael G. Branstetter, Bonnie B. Blaimer, Marek L. Borowiec, Gabriela P. Camacho, Maël Doré, Philip S. Ward, John T. Longino

Supplementary material 2 Sequencing statistics for samples included in the phylogenomic dataset Data type xlsx Explanation note Sequencing statistics for samples included in the phylogenomic dataset. This table lists sequencing, assembly, and voucher information for the 1,170 samples analyzed in this study, comprising 1,167 ingroup terminals representing species of Ponerinae and three outgroup taxa (*Paraponera clavata*, *Amblyopone australis*, and *Proceratium google*). For each sample, the following metadata and sequencing statistics are provided: extraction code, sample name, BioProject ID, BioSample ID, SRA accession, COX1 GenBank accession, reference, number of paired-end reads, number of UCE loci recovered, total assembled length, mean contig length, standard error, minimum length, maximum length, median length, the number of contigs > 1 kb, voucher specimen code, and country. Additional voucher data can be examined on AntWeb (https://www.antweb.org/). https://binary.pensoft.net/file/1496662This dataset is made available under the Open Database License (http://opendatacommons.org/licenses/odbl/1.0/). The Open Database License (ODbL) is a license agreement intended to allow users to freely share, modify, and use this Dataset while maintaining this same freedom for others, provided that the original source and author(s) are credited.Brian L. Fisher, Michael G. Branstetter, Bonnie B. Blaimer, Marek L. Borowiec, Gabriela P. Camacho, Maël Doré, Philip S. Ward, John T. Longino

Supplementary material 3 Extended phylogeny of the ant subfamily Ponerinae Data type pdf Explanation note Extended phylogeny of the ant subfamily Ponerinae. Maximum likelihood phylogeny generated in IQ-TREE v. 2.4.0 from the full 1167-taxon dataset in an unpartitioned analysis employing a GTR+F+G4 model. All branches are 100% supported by ultrafast bootstraps (ufBS) and SH-like approximate likelihood tests (SH-aLRT), except where indicated. The new genus-level classification is indicated with the vertical colored bars; genus groups are indicated for the five groups containing more than one genus. https://binary.pensoft.net/file/1496663This dataset is made available under the Open Database License (http://opendatacommons.org/licenses/odbl/1.0/). The Open Database License (ODbL) is a license agreement intended to allow users to freely share, modify, and use this Dataset while maintaining this same freedom for others, provided that the original source and author(s) are credited.Brian L. Fisher, Michael G. Branstetter, Bonnie B. Blaimer, Marek L. Borowiec, Gabriela P. Camacho, Maël Doré, Philip S. Ward, John T. Longino
