# Supplementary material for: A genus-level classification of the ant subfamily Ponerinae (Hymenoptera, Formicidae)
Source: Zookeys. 2025 Dec 19;1264:281–349. doi: 10.3897/zookeys.1264.173399 (PMC12743250; doi:10.3897/zookeys.1264.173399)
Supplement: Supplementary material 3 — Extended phylogeny of the ant subfamily Ponerinae [file zookeys-1264-281_article-173399__-s003.pdf]

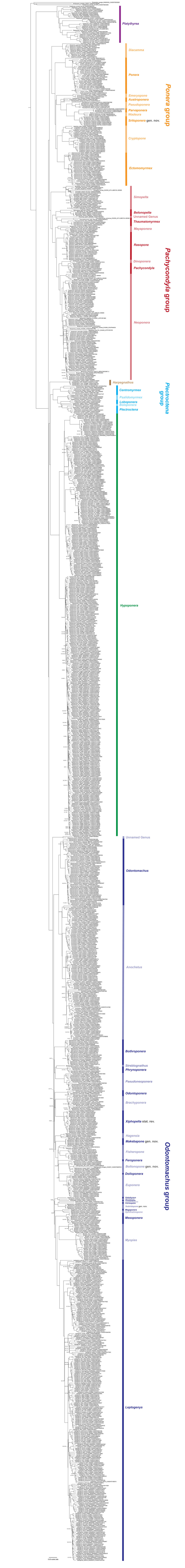
Platythyrea
Diacamma
Ponera
Emeryopone
Austroponera
Pseudoponera
Parvaponera
Wadeura
Sritoponera gen. nov.
Cryptopone
Ectomomyrmex
Ponera group
Simopelta
Belonopelta
Unnamed Genus
Thaumatomyrmex
Mayaponera
Rasopone
Dinoponera
Pachycondyla
Neoponera
Pachycondyla group
Harpegnathos
Centromyrmex
Psalidomyrmex
Loboponera
Boloponera
Plectroctena
Plectroctena group
Hypoponera
Unnamed Genus
Odontomachus
Anochetus
Bothroponera
Streblognathus
Phrynoponera
Pseudoneoponera
Odontoponera
Brachyponera
Xiphopelta stat. rev.
Hagensia
Makebapone gen. nov.
Fisheropone
Feroponera
Boltonopone gen. nov.
Dolioponera
Euponera
Paltothyreus
Buniapone
Promyopias
Asphinctopone
Corrieopone
Subiridopone gen. nov.
Megaponera
Ophthalmopone
Mesoponera
Myopias
Leptogenys
Odontomachus group
